# Integrative analysis and expression profiling of secondary cell wall genes in C_4_ biofuel model *Setaria italica* reveals targets for lignocellulose bioengineering

**DOI:** 10.3389/fpls.2015.00965

**Published:** 2015-11-04

**Authors:** Mehanathan Muthamilarasan, Yusuf Khan, Jananee Jaishankar, Shweta Shweta, Charu Lata, Manoj Prasad

**Affiliations:** ^1^National Institute of Plant Genome ResearchNew Delhi, India; ^2^Division of Plant-Microbe Interactions, CSIR-National Botanical Research InstituteLucknow, India

**Keywords:** foxtail millet (*Setaria italica* L.), secondary cell wall biosynthesis, lignocellulose, bioenergy grasses, genomics, comparative mapping

## Abstract

Several underutilized grasses have excellent potential for use as bioenergy feedstock due to their lignocellulosic biomass. Genomic tools have enabled identification of lignocellulose biosynthesis genes in several sequenced plants. However, the non-availability of whole genome sequence of bioenergy grasses hinders the study on bioenergy genomics and their genomics-assisted crop improvement. Foxtail millet (*Setaria italica* L.; Si) is a model crop for studying systems biology of bioenergy grasses. In the present study, a systematic approach has been used for identification of gene families involved in cellulose (*CesA/Csl*), callose (*Gsl*) and monolignol biosynthesis (*PAL, C4H, 4CL, HCT, C3H, CCoAOMT, F5H, COMT, CCR, CAD*) and construction of physical map of foxtail millet. Sequence alignment and phylogenetic analysis of identified proteins showed that monolignol biosynthesis proteins were highly diverse, whereas CesA/Csl and Gsl proteins were homologous to rice and *Arabidopsis*. Comparative mapping of foxtail millet lignocellulose biosynthesis genes with other C_4_ panicoid genomes revealed maximum homology with switchgrass, followed by sorghum and maize. Expression profiling of candidate lignocellulose genes in response to different abiotic stresses and hormone treatments showed their differential expression pattern, with significant higher expression of *SiGsl12, SiPAL2, SiHCT1, SiF5H2*, and *SiCAD6* genes. Further, due to the evolutionary conservation of grass genomes, the insights gained from the present study could be extrapolated for identifying genes involved in lignocellulose biosynthesis in other biofuel species for further characterization.

## Introduction

Cell wall polymers of living plants constitute a predominant proportion of their biomass, which is formed by fermentable linked sugars. These polymers form a major structural component of plant cell wall and particularly, secondary cell walls provide mechanical strength and rigidity to vascular plants (Wang et al., 2013; Zhong and Ye, 2015). Secondary cell walls are present in tracheary elements, xylem, phloem, extraxylary and interfascicular fibers, sclereids and seed coats, and are made of cellulose, hemicelluloses and lignin. Cellulose, the primary unit, cross-links with hemicelluloses including xylan and glucomannan, and impregnated with phenolic polymer lignin, and altogether, this complex polymeric network forms secondary cell wall. The proportion of cellulose, hemicelluloses, and lignin varies among different plant species and of note, the composition may also vary in response to diverse developmental and environmental conditions (Zhong and Ye, 2015). Being the prime constituents of wood and fiber, secondary cell walls have been extensively studied to understand and exploit their biofuel prospects. Biochemical and genomic methods have identified the genes encoding for enzymes which participate in the biosynthesis of secondary cell wall components.

Pear et al. (1996) was the first to identify *cellulose synthase* (*CesA*) genes in cotton and following this, *CesA* genes in other plants have been identified and their numbers were shown to vary between plant species. In *Arabidopsis*, 10 *CesA* genes have been identified (Richmond and Somerville, 2000), whereas 12 in maize (Appenzeller et al., 2004), 16 in barley (Burton et al., 2004), 18 in poplar (Djerbi et al., 2005) have been reported. The CesA enzymes belong to glycosyltransferase-2 (GT-2) superfamily, which is defined by an eight-transmembrane topology and conserved cytosolic substrate binding and catalytic residues (McFarlane et al., 2014). In addition to *CesA*, plants also have *cellulose synthase-like* (*Csl*) genes, which can be involved in biosynthesis of hemicellulose and other glucans (Lerouxel et al., 2006). *Csl* genes can synthesize other polysaccharides that are not components of the hemicellulose matrix (Lerouxel et al., 2006). So far, several types of *Csl* genes have been identified, denoted as *CslA* to *CslK*. *CslA* encodes for (1,4)-β-D-mannan synthases (Dhugga et al., 2004; Liepman et al., 2005), *CslF* and *CslH* encode the mixed linkage glucan synthases for (1,3;1,4)-β-glucan biosynthesis (Burton et al., 2006; Doblin et al., 2009), *CslC* genes are involved in xyloglucan biosynthesis (Cocuron et al., 2007), and *CslD* in xylan and homogalacturonan synthesis (Hamann et al., 2004; Bernal et al., 2008a,b; Li et al., 2009), whereas the functional roles of other *Csl* genes remain elusive (Yin et al., 2009). Noteworthy, *CslB* and *CslG* are specific to dicots whereas *CslF* and *CslH* are found only in monocots (Fincher, 2009; Doblin et al., 2010), but recently two *CslG* genes were identified in *Panicum virgatum* (*Pavirv00027268m* and *Pavirv00027269m*; Yin et al., 2014).

Callose is a (1,3)-β-D-glucan, which is not present in cell walls but deposited in the walls of specialized tissues such as pollen mother cell walls, plasmodesmatal canals, and sieve plates in dormant phloem during normal growth and development (Stone and Clarke, 1992). In addition, callose is also deposited in response to environmental stimuli including abiotic stress, wounding, and pathogen challenge (Stone and Clarke, 1992; Muthamilarasan and Prasad, 2013). Callose is synthesized by callose synthases, which are encoded by *glucan synthase-like* (*Gsl*) genes (Saxena and Brown, 2000; Cui et al., 2001). To date, 12 *Gsl* genes have been identified in *Arabidopsis*, 13 in rice, 9 in poplar, and 8 in barley (Farrokhi et al., 2006).

In the case of lignin biosynthesis, phenylalanine is metabolized through the phenylpropanoid pathway to produce hydroxycinnamoyl-CoA esters, which enter the lignin branch of this pathway and are converted to monolignols. The process requires the involvement of phenylalanine ammonia lyase (PAL), trans-cinnamate 4-hydroxylase (C4H), 4-coumarate CoA ligase (4CL), hydroxycinnamoyl CoA:shikimate/quinate hydroxycinnamoyl transferase (HCT), *p*-coumaroyl shikimate 3′-hydroxylase (C3H), caffeoyl CoA 3-*O*-methyltransferase (CCoAOMT), ferulate 5-hydroxylase (F5H), caffeic acid *O*-methyltransferase (COMT), cinnamoyl CoA reductase (CCR), and cinnamyl alcohol dehydrogenase (CAD) (Bonawitz and Chapple, 2010; Zhong and Ye, 2015). Of these enzymes, PAL is the first enzyme of phenylpropanoid pathway which catalyzes the deamination of phenylalanine to generate cinnamic acid and C4H hydroxylates cinnamic acid to generate p-coumaric acid (Harakava, 2005). 4CL performs CoA esterification of *p*-coumaric acid and caffeic acid, whereas HCT catalyzes the conversion of *p*-coumaroyl-CoA and caffeoyl-CoA into corresponding shikimate or quinate esters and C3H converts these esters to corresponding caffeoyl esters. Following this, CCoAOMT catalyzes methylation of caffeoyl CoA to produce feruloyl CoA, whereas CCR converts hydroxycinnamoyl CoA esters to their corresponding aldehydes (Harakava, 2005). F5H has been assumed to catalyze the conversion of ferulic acid to 5-hydroxyferulic acid but recombinant DNA studies in *Arabidopsis* and *Liquidambar styraciflua* revealed that F5H converts coniferaldehyde and coniferyl alcohol to synapaldehyde and sinapyl alcohol, respectively (Humphreys et al., 1999; Osakabe et al., 1999). COMT is involved in the conversion of 5-hydroxyconiferaldehyde and/or 5-hydroxyconiferyl alcohol to sinapaldehyde and/or sinapyl alcohol, respectively (Osakabe et al., 1999; Parvathi et al., 2001), while CAD catalyzes the conversion of cinnamyl aldehydes into their corresponding alcohols (Harakava, 2005). The genes encoding these enzymes have recently been identified and characterized in several plant species (Raes et al., 2003; Vanholme et al., 2012; Shen et al., 2013; Carocha et al., 2015; van Parijs et al., 2015).

With the raise in the impacts of global climate change, reduction of greenhouse gases is essential, which could be facilitated through generating biorenewables. Importantly, production of lignocellulosic biofuels from secondary cell wall biomass has become a strategic research area, as it holds the potential to enhance energy security. C_4_ grasses, namely switchgrass (*P. virgatum*), napier grass (*Pennisetum purpureum*), pearl millet (*P. glaucum*), and foxtail millet (*Setaria italica*) have recently gained momentum in lignocellulosic biofuel research due to their high-efficiency CO_2_ fixation and efficient conversion of solar energy to biomass through C_4_ photosynthesis and photorespiration-suppressing modifications, respectively (Schmer et al., 2008; Byrt et al., 2011; van der Weijde et al., 2013). In addition, these grasses also possess better water use efficiency (WUE), higher nitrogen use efficiency (NUE), capacity to grow in arid and semi-arid regions and relatively high tolerance to environmental constraints including heat, drought, salinity and water-logging. For these reasons, C_4_ photosynthesis is an important trait for lignocellulosic biofuel crops (Byrt et al., 2011; van der Weijde et al., 2013).

Recently, foxtail millet (*S. italica*) and its wild progenitor, green foxtail (*S. viridis*) have been recognized as the suitable experimental models for biofuel research owing to their genetic relatedness to several biofuel grasses (Li and Brutnell, 2011; Zhang et al., 2012; Lata et al., 2013; Petti et al., 2013; Diao et al., 2014; Warnasooriya and Brutnell, 2014; Muthamilarasan and Prasad, 2015). The genomes of both foxtail millet and green foxtail have been sequenced (Bennetzen et al., 2012; Zhang et al., 2012), and the availability of foxtail millet draft genome sequence in public domains has facilitated various genetic and genomic studies in this model crop pertaining to stress response and crop improvement (Diao et al., 2014; Muthamilarasan and Prasad, 2015; Muthamilarasan et al., 2015) though no comprehensive genome-wide study on biofuel traits has been performed. Recently, Petti et al. (2013) has compared the lignocellulosic feedstock composition, cellulose biosynthesis inhibitor response, saccharification dynamics and *CesA* gene family of green foxtail with sorghum, maize and switchgrass. The study identified eight potential *CesA* gene family members for functional genomic characterization (Petti et al., 2013).

The present study has been performed to identify the gene families participating in lignocellulose biosynthesis using computational approaches. Further, qRT-PCR analysis of few genes has been performed to understand their expression patterns in response to different abiotic stress treatments.

## Materials and methods

### Identification of lignocellulose biosynthesis gene families

Protein sequences of enzymes involved in cellulose biosynthesis, namely CesA, Csl, and Gsl of rice and *Arabidopsis* were retrieved from cell wall genomics webserver (https://cellwall.genomics.purdue.edu/intro/index.html). The sequences for PAL, C4H, 4CL, HCT, C3H, CCoAOMT, F5H, COMT, CCR, and CAD reported in other crops (Appenzeller et al., 2004; Burton et al., 2004; Carocha et al., 2015; Zhong and Ye, 2015) were retrieved from respective literatures and HMM profile has been generated for individual families. Precisely, the sequences of respective families were aligned using Clustal Omega (http://www.ebi.ac.uk/Tools/msa/clustalo/) and HMM profiles were built using hmmbuilt command (http://hmmer.janelia.org/). HMMER tool was used to identify respective homologous proteins in foxtail millet protein dataset retrieved from Phytozome v10.2 (http://phytozome.jgi.doe.gov/) under default parameters (Muthamilarasan et al., 2014a). The protein sequences were confirmed using HMMSCAN (http://www.ebi.ac.uk/Tools/hmmer/search/hmmscan) analysis, and respective genomic, transcript, and CDS sequences were downloaded from Phytozome by BLAST searching the retrieved protein sequences against *S. italica* database under default parameters (http://phytozome.jgi.doe.gov/pz/portal.html#!info?alias=Org_Sitalica).

### Protein properties and phylogenetic analysis

The properties of identified cell wall-related proteins including molecular weight, pI, and instability index were identified using ExPASy ProtPram tool (http://web.expasy.org/protparam/). The amino acid sequences of respective families were imported into MEGA v6 (Tamura et al., 2013) for multiple sequence alignment and phylogenetic tree construction using neighbor-joining method after bootstrap analysis for 1000 replicates (Muthamilarasan et al., 2014b). Sequence alignment and analysis was performed using BioEdit v7.2.5 (http://www.mbio.ncsu.edu/bioedit/bioedit.html).

### Physical mapping and gene structure analysis

The chromosomal location of cell wall biosynthesis genes including chromosome number, position of gene start and end, gene length and orientation were obtained from Phytozome and a physical map was constructed using MapChart (Voorrips, 2002). Gene duplications, namely tandem and segmental were identified by performing MCScanx (Wang et al., 2012) according to the protocol of Plant Genome Duplication Database (Lee et al., 2012). Gene structure was predicted using Gene Structure Display Server v2.0 (http://gsds.cbi.pku.edu.cn/).

### Promoter analysis, targeting miRNA, and marker prediction

The upstream genomic sequence (~2 kb) of lignocellulose pathway genes of foxtail millet were retrieved from Phytozome and the presence of *cis*-regulatory elements were identified by Signal Scan Search using New PLACE web server (https://sogo.dna.affrc.go.jp/cgi-bin/sogo.cgi?page=analysis&lang=en). Mature miRNA sequences of foxtail millet were downloaded from miRBase v21 (Kozomara and Griffiths-Jones, 2014) and FmMiRNADb (Khan et al., 2014). This information along with the miRNA data of a dehydration stress library (Yadav et al., unpublished data) were used to identify the miRNAs targeting the transcripts of lignocellulose pathway genes using psRNAtarget server (Dai and Zhao, 2011) under default parameters. The large-scale genome-wide molecular markers namely simple sequence repeats (SSR; Pandey et al., 2013), expressed sequence tag (EST)-SSR (eSSR; Kumari et al., 2013), and intron-length polymorphic markers (Muthamilarasan et al., 2014c) were retrieved from the Foxtail millet Marker Database (http://www.nipgr.res.in/foxtail.html; Suresh et al., 2013) and searched for their presence in the genic and promoter regions of lignocellulose biosynthesis genes using in-house perl script.

### Comparative genome mapping and evolutionary analysis

Protein sequences of lignocellulose pathway genes of foxtail millet were BLASTP searched against the protein sequences of switchgrass (*Panicum virgatum*), rice (*Oryza sativa*), and poplar (*Populus trichocarpa*), and hits with more than 80% identity were selected. The genomic and CDS sequences along with chromosomal locations for these proteins were retrieved by performing BLAST searches against the corresponding genomes retrieved from Gramene (http://www.gramene.org/) under default parameters and comparative maps were visualized using Circos (Krzywinski et al., 2009). Reciprocal BLAST was also performed to ensure the unique relationship between the homologous genes (Mishra et al., 2013). Estimation of nonsynonymous substitutions per non-synonymous site (Ka) and synonymous substitutions per synonymous site (Ks) for paralogous (tandem and segmentally duplicated genes) as well as homologous (comparative mapping data) gene pairs were calculated by codeml program in PAML using PAL2NAL (Suyama et al., 2006). The Ka/Ks ratios along with estimation of duplication and divergence (as T = Ks/2λ, where, λ = 6.5 × 10^−9^) were performed according to Puranik et al. (2013).

### *In silico* expression profiling in tissues and drought stress

The transcriptome data of different tissues, namely root (SRX128223), stem (SRX128225), leaf (SRX128224), spica (SRX128226), and a drought stress library (SRR629694) as well as its control (SRR629695) were retrieved from European Nucleotide Archive (http://www.ebi.ac.uk/ena) (Zhang et al., 2012; Qi et al., 2013). The reads were filtered using NGS Toolkit (http://www.nipgr.res.in/ngsqctoolkit.html), mapped on foxtail millet genome using CLC Genomics Workbench v4.7.1, normalized by RPKM method and a heat map was generated using MultiExperiment Viewer (MeV) v4.9 (Saeed et al., 2003).

### Plant materials, stress and hormone treatments and quantitative real-time PCR analysis

Seeds of foxtail millet cv. “IC-403579” (dehydration and salinity tolerant) were grown under optimum conditions following Lata et al. (2014). Twenty one day-old seedlings were exposed to 250 mM NaCl (salinity), 20% PEG6000 (dehydration), 4°C (cold), 100 mM abscisic acid (ABA), 100 mM methyl jasmonate (MeJA), and 100 mM salicylic acid (SA) treatments (Mishra et al., 2013; Puranik et al., 2013; Kumar et al., 2015) and whole seedlings were collected at 0 h (h) (control), 1 h (early), and 24 h (late) (Yadav et al., 2015). The samples were frozen immediately in liquid nitrogen and stored at −80°C. RNA isolation, cDNA synthesis and RT-PCR analysis were performed according to Puranik et al. (2013) in three technical replicates for each biological triplicate using the primers mentioned in Supplementary Table S1. All qRT-PCR data were the means of at least three independent experiments and the results were presented as the mean values ± SE. The significance of differences between mean values of control and each stressed samples were statistically performed using One-Way analysis of variance (ANOVA) and comparison among means was carried out through Tukey-Kramer multiple comparisons test using GRAPHPAD INSTAT software v3.10 (http://www.graphpad.com). The differences in the effects of stress treatments on various parameters in 16 foxtail millet genes under study were considered statistically significant at ^*^P < 0.05, ^**^P < 0.01, ^***^P < 0.001.

## Results

### CesA/Csl and Gsl superfamily of foxtail millet

HMM searches identified the presence of 14 CesA (SiCesA) and 39 Csl (SiCsl) proteins in foxtail millet (Supplementary Table S2). Among the 14 SiCesA proteins, one was found to be an alternate transcript (Si028766m), whereas in SiCsl, three alternate transcripts (Si029554m, Si035399m, and Si035101m) were identified. Domain analysis of SiCesA proteins revealed the presence of both the cellulose synthase domain (CS; PF03552) and the zinc finger structure (ZF; PF14569) in all the proteins except SiCesA8 and SiCesA10, which have only the CS domain (Supplementary Table S3). In addition, all the SiCesA proteins except SiCesA8 had Glycosyl transferase 2 (GT2; PF13632) domain. In the case of SiCsl proteins, 36 proteins (primary transcripts) were identified, of which 10 belonged to SiCslA, 6 to SiCslC, 5 to SiCslD, 4 to SiCslE, 7 to SiCslF, 2 each to SiCslH and SiCslJ families (Supplementary Table S2). Interestingly, two members of CslJ have been identified in foxtail millet, which was previously considered to be a cereal-specific gene family (Doblin et al., 2010). Domain analysis showed that all the SiCslA and SiCslC proteins possess GT2 domain (PF13641, PF13632, PF00535, and PF13506) (Supplementary Table S3).

All 5 SiCslD proteins possess CS (PF03552) and GT2 (PF13632) domain, and interestingly, SiCslD2, SiCslD4, and SiCslD5 were evidenced to have an additional RING/Ubox like zinc-binding domain (PF14570), whereas SiCslD3 has two CS domains (Supplementary Table S4). All the SiCslE proteins except SiCslE2 have more than one CS domain and SiCslE3 has an additional GT2 domain (PF13641). In the case of SiCslF proteins, all of the members except SiCslF6 have two CS domains and in addition, SiCslF1, SiCslF3, and SiCslF7 possess GT2 domain (PF13632). Two members each belonging to CslH and CslJ family proteins were identified and both the group members have two CS domains (Supplementary Table S4).

A total of 12 Gsl (SiGsl) proteins were identified in foxtail millet and all possessed glucan synthesis (GS) domain (1,3-beta-glucan synthase component; PF02364) (Supplementary Table S5). The number of GS domain within these proteins also varied as SiGsl1, SiGsl6, and SiGsl12 have two GS domains, whereas SiGsl11 had three domains. In addition, SiGsl2, SiGsl3, SiGsl5, SiGsl7, SiGsl8, SiGsl10, and SiGsl11 have a 1,3-beta-glucan synthase subunit FKS1, domain-1 (PF14288). Furthermore, SiGsl08, SiGsl10, and SiGsl11 have an additional Vta1 (VPS20-associated protein 1) like domain (PF04652) (Supplementary Table S5).

### Monolignol pathway proteins of foxtail millet

HMM profiling of PAL (SiPAL), C4H (SiC4H), 4CL (Si4CL), HCT (SiHCT), C3H (SiC3H), CCoAOMT (SiCCoAOMT), F5H (SiF5H), COMT (SiCOMT), CCR (SiCCR), and CAD (SiCAD) proteins in foxtail millet identified 10, 3, 20, 2, 2, 6, 2, 4, 33, and 13 members, respectively (Supplementary Table S6). Splice variants were evidenced among these members, including three each in SiCL16 and SiCCR14, two in SiCCR11 and one each in Si4CL5, SiCCoAOMT1, SiCOMT, SiCCR11, and SiCCR17. HMMSCAN revealed a diverse domain organization of these proteins (Supplementary Table S7). All of the SiPAL proteins possess aromatic amino acid lyase (PF00221) domain, whereas Cytochrome P450 (PF00067) was present in all SiC4H, SiC3H, and SiF5H proteins. AMP-binding enzyme (PF00501) and AMP-binding enzyme C-terminal (PF13193) domains were present in all the Si4CL proteins except Si4CL13, which has only an AMP-binding enzyme domain. Both SiHCT1 and SiHCT2 have transferase family (PF02458) domains, and SiCCoAOMT proteins were evidenced to possess O-methyltransferase (PF01596) and methyltransferase (PF13578) domains with an exception of SiCCoAOMT, which has two O-methyltransferase domains (Supplementary Table S7). O-methyltransferase domain was also found to be present in SiCOMT proteins, whereas SiCOMT2 has an additional dimerisation domain (PF13578). A diverse domain composition was observed among SiCCR proteins in addition to the presence of signature NAD-dependent epimerase/dehydratase family (PF01370) and 3-beta hydroxysteroid dehydrogenase/isomerase family (PF01073) domains. Almost all the SiCCR proteins possess additional domains including GDP-mannose-4,6-dehydratase (PF16363), Male sterility protein (PF07993), NmrA-like family (PF05368), NAD(P)H-binding (PF13460), Polysaccharide biosynthesis protein (PF02719), and KR domains (PF08659). Of note, SiCCR7 was devoid of any of these domains except the NAD-dependent epimerase/dehydratase family domain, and SiCCR3 has an additional Alcohol dehydrogenase GroES-like domain (PF08240) (Supplementary Table S7). The presence of Alcohol dehydrogenase GroES-like and Zinc-binding dehydrogenase (PF00107) domains is the characteristic feature of SiCAD proteins and in addition to these, D-isomer specific 2-hydroxyacid dehydrogenase, NAD-binding domain (PF02826) was present in SiCAD4, SiCAD9, and SiCAD12. Moreover, an alanine dehydrogenase/PNT, C-terminal domain (PF01262) was found to be present in SiCAD12 and SiCAD13 (Supplementary Table S7).

### Properties of lignocellulose pathway proteins

Among the SiCesA proteins, SiCesA4 was the largest protein with 1095 amino acids (aa), followed by SiCesA2 (1092 aa), SiCesA11 (1090 aa) and SiCesA3 (1088 aa), and the smallest was SiCesA8 (884 aa) (Supplementary Table S2). The molecular weight of these proteins also varied accordingly, ranging from SiCesA8 (95.5 kDa) to SiCesA11 (123.2 kDa), with an isoelectric pH (pI) of 6.03 (SiCesA10) to 8.15 (SiCesA1). The protein instability index was between 36.07 (SiCesA11) to 50.62 (SiCesA8), which signified that all the SiCesA proteins except SiCesA2, SiCesA8, and SiCesA10 were stable. In the case of SiCsl proteins, the smallest protein was SiCslE2 with 144 aa and the largest was SiCslD1 (1217 aa), and their respective molecular weights ranged from 16.4 kDa (SiCslE2) to 132.2 kDa (SiCslD1). The pI of SiCsl proteins ranged from 4.61 (SiCslE2) to 9.32 (SiCslF7), and their instability index range (31.44–67.71) revealed that a maximum of SiCsl proteins (~33%) were stable. The size and molecular weights of SiGsl proteins ranged from 418 aa (47.8 kDa in SiGsl9) to 1956 aa (225.2 kDa in SiGsl8). Similarly, pI range of these proteins was between 8.61 (SiGsl12) and 9.69 (SiGsl9). The instability index range between 28.89 and 52.08 indicated that ~46% of SiGsl proteins were stable and the rest are unstable (Supplementary Table S2).

The SiPAL class of monolignol pathway proteins showed a narrow range of protein properties, as their sizes varied from 699 (SiPAL1 and SiPAL2) to 851 aa (SiPAL10), with molecular weights from 74.9 kDa (SiPAL2) to 91.1 kDa (SiPAL10) (Supplementary Table S6). The pI range of SiPAL was between 5.82 and 6.52, and their instability index range (28.82–39.84) showed that all the proteins except SiPAL5 were stable. The three members of SiC4H, namely SiC4H1, SiC4H2, and SiC4H3 had molecular sizes of 530 aa (59.7 kDa), 430 aa (49.3 kDa), and 506 aa (57.9 kDa), respectively. Their respective pI were 9.26, 7.72, and 9.33, and their instability index (46.46, 49.84, and 48.61) revealed that SiC4H proteins were stable. Among the Si4CL proteins, Si4CL4 and Si4CL10 were the smallest proteins with 198 aa (21.8 and 21.7 kDa in size, respectively) and the largest was Si4CL9 (642 aa; 68.5 kDa). Their pI range was between 5.14 and 8.98. The protein instability index ranged from 24.76 (Si4CL3) to 47.96 (Si4CL6) hinting that all the Si4CL proteins except Si4CL3 were stable. SiHCT, SiC3H, and SiF5H proteins have two members each, with a narrow range of protein properties, and all these proteins were found to be stable as indicated by their stability index. A significant difference was observed with the sizes of SiF5H members since SiF5H1 was 158 aa (16.7 kDa) and SiF5H2 was 524 aa (57.7 kDa) (Supplementary Table S6). Among SiCCoAOMT proteins, the smallest protein was SiCCoAOMT1 with 243 aa (25.7 kDa) and the largest was SiCCoAOMT5 with 307 aa (33.4 kDa). The pI range was between 5.04 and 8.94, and the protein instability index range (27.69–51.49) showed that except SiCCoAOMT4, all others were stable. The three-member SiCOMT class proteins have molecular sizes of 247 aa (25.8 kDa; SiCOMT1), 402 aa (43.53 kDa; SiCOMT2), and 153 aa (16.71 kDa; SiCOMT3). The pI values were 5.09, 5.97, and 9 for SiCOMT1, SiCOMT2, and SiCOMT3, respectively. The instability index range (42.24–52.75) hinted that all SiCOMT proteins are stable. Among the monolignol pathway proteins, SiCCR class has the highest number (26 members) and their sizes ranged from 27.2 kDa (251 aa; SiCCR26) to 69.13 kDa (625 aa; SiCCR9), with a pI range of 4.72 (SiCCR23) to 9.32 (SiCCR19). The protein instability index ranged from 24.86 (SiCCR18) to 54.11 (SiCCR13), which points out that ~77% of SiCCR proteins were stable. In the case of SiCAD proteins, SiCAD9 and SiCAD13 were the smallest proteins with 336 aa (35.6 and 36.4 kDa in size, respectively) and SiCAD8 was the largest with 495 aa (52.7 kDa). The pI ranged from 5.05 to 9.24, and the instability index (19.35–39.79) showed that ~50% of SiCAD proteins are unstable (Supplementary Table S6).

### Sequence alignment and phylogenetic analysis of CesA/Csl and Gsl proteins

**Figure 1:**
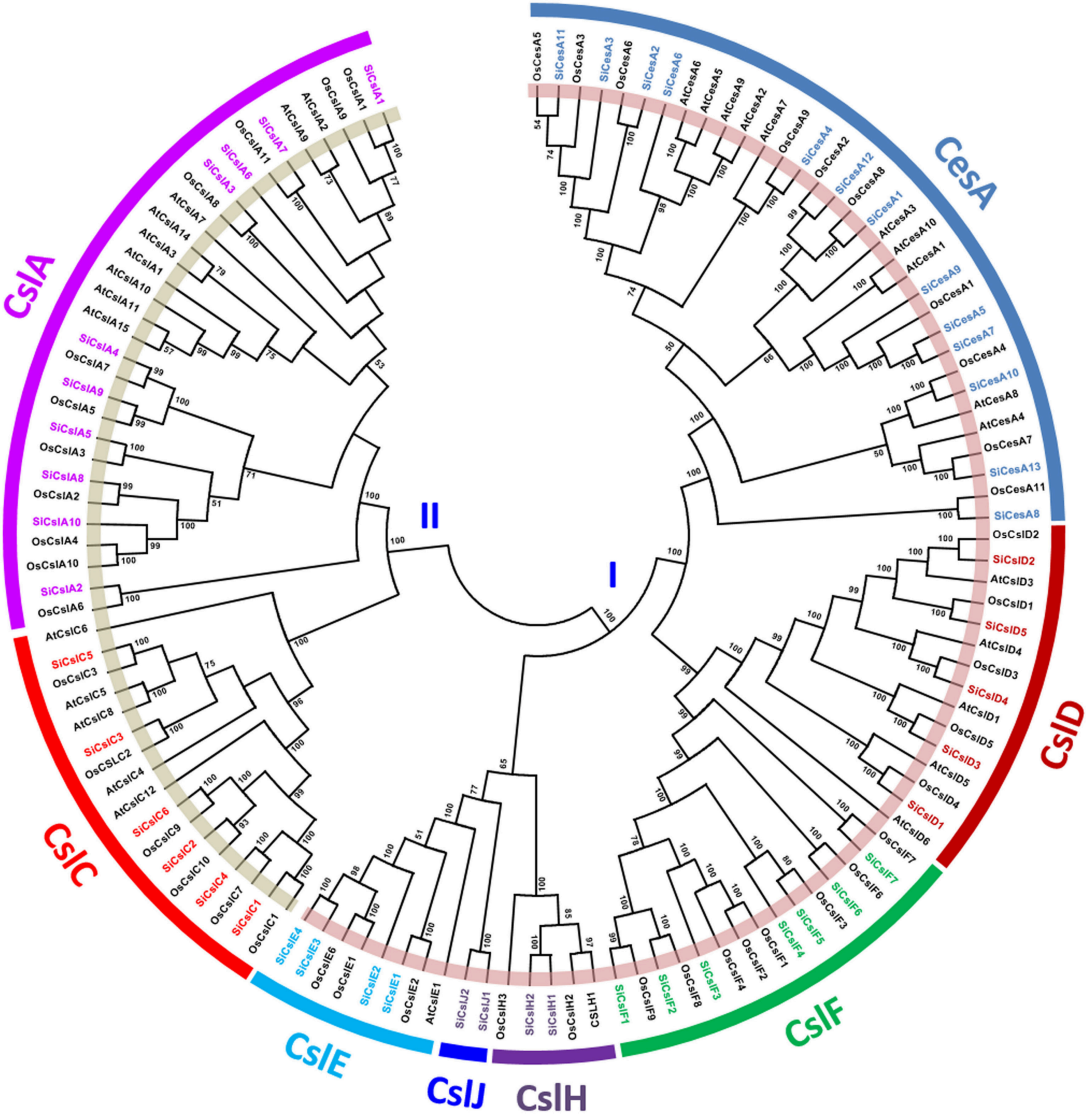
**Unrooted protein phylogenetic tree constructed with CesA/Csl proteins of *Setaria italica* (Si), *Oryza sativa* (Os), and *Arabidopsis thaliana* (At)**.

SiCesA and SiCsl proteins were aligned individually, and the alignment revealed the presence of conserved “DXD, D, QXXRW” motif in both the superfamilies. All the SiCesA proteins except SiCesA8 have a “DCD, D, QVLRW” consensus sequence, whereas SiCesA8 had a unique “DYD, D” sequence and the motif “QXXRW” was absent (Supplementary Figure S1). Noteworthy, SiCesA8 protein has only the CS domain, while the other SiCesA proteins possess CS, ZF, and GT2 domains (Supplementary Table S3). In the case of SiCsl proteins, the “DXD” motif is absent in all the members of SiCslA, SiCslC and SiCslE2 (Supplementary Figure S2). This motif was predominantly “DCD,” except in SiCslF1 and SiCslF2, which have “DGD.” The second consensus “D” amino acid is present in all the SiCsl members (as “ED”), except SiCslA6, SiCslE2, and SiCslF4 (Supplementary Figure S2). In addition, SiCslA6 and SiCslE2 did not possess the “QXXRW” motif also, whereas a subgroup-wise conservation was evidenced in this motif in rest of the members. The majority of SiCslA (7) and all the SiCslC members have “QQHRW” motif, whereas SiCslE proteins have “QHKRW,” SiCslH and SiCslJ proteins have “QYKRW” and “QNKRW” motifs, respectively (Supplementary Figure S2). The unrooted phylogenetic tree constructed using the amino acid sequences of SiCesA/Csl proteins along with CesA/Csl proteins of rice and *Arabidopsis* (https://cellwall.genomics.purdue.edu/intro/index.html) showed 2 distinct clusters, namely I and II (Figure 1). Cluster I was resolved into six branches including CesA, CslD, CslE, CslF, CslH, and CslJ, whereas cluster II had two branches, CslA and CslC.

**Figure 2:**
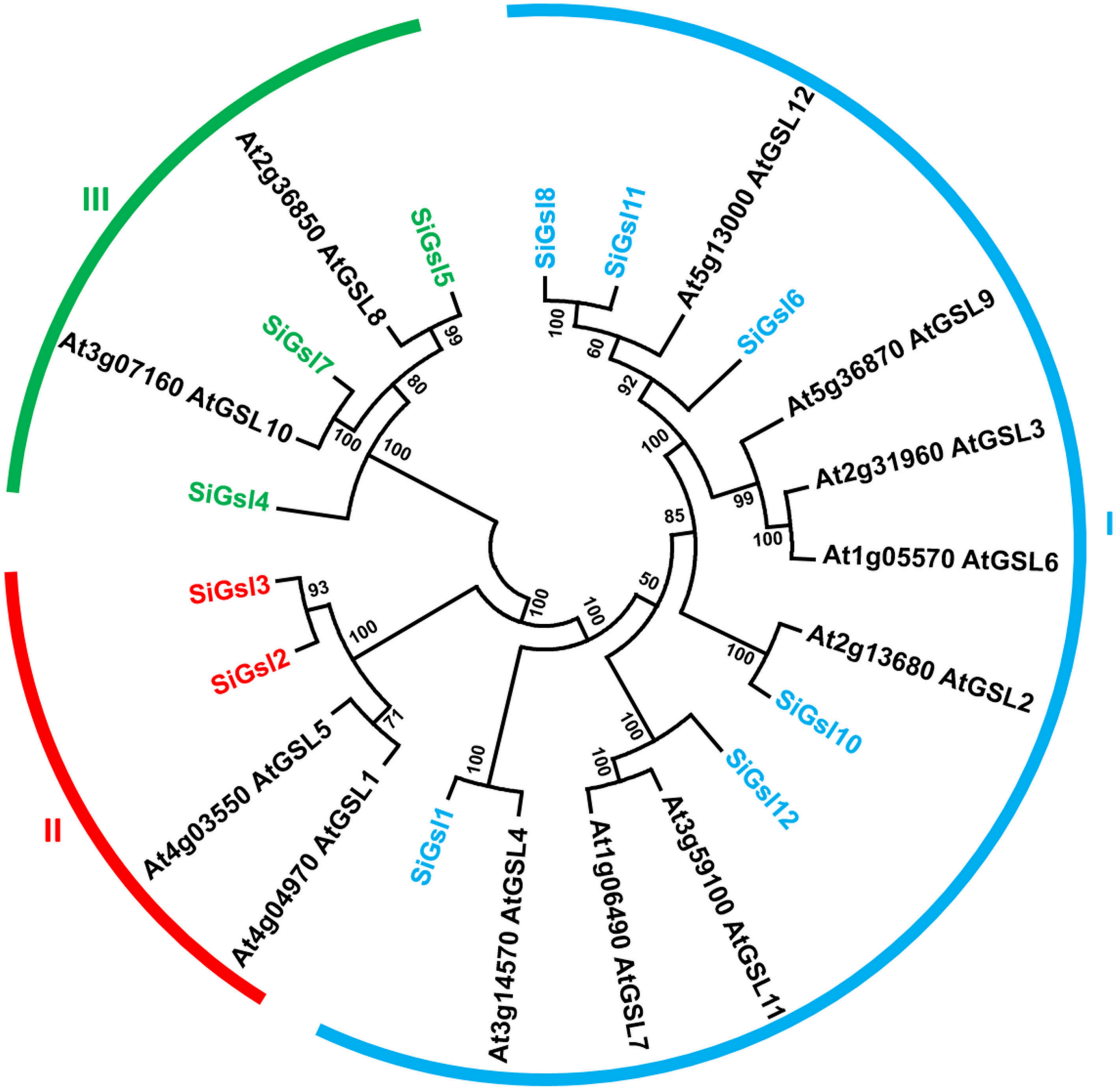
**Unrooted protein phylogenetic tree constructed with Gsl proteins of *Setaria italica* (Si) and *Arabidopsis thaliana* (At)**.

Sequence alignment of SiGsl proteins showed that the N-terminal region of all these proteins was diverse, whereas the C-terminal region was conserved (Supplementary Figure S3). Prediction of transmembrane (TM) helices in these proteins using TMHMM Server v2.0 (http://www.cbs.dtu.dk/services/TMHMM/) showed the presence of 7–16 TM helices in SiGsl proteins (Supplementary Figure S4). Phylogeny of foxtail millet, rice and *Arabidopsis* Gsl proteins showed three clusters (Figure 2). Cluster I included SiGsl4, SiGsl5, and SiGsl7, whereas cluster II comprised SiGsl2 and SiGsl3. SiGsl1, SiGsl6, SiGsl8, SiGsl10, SiGsl11, and SiGsl12 were included in cluster III.

### Sequence alignment and phylogenetic analysis of monolignol biosynthesis pathway proteins

**Figure 3:**
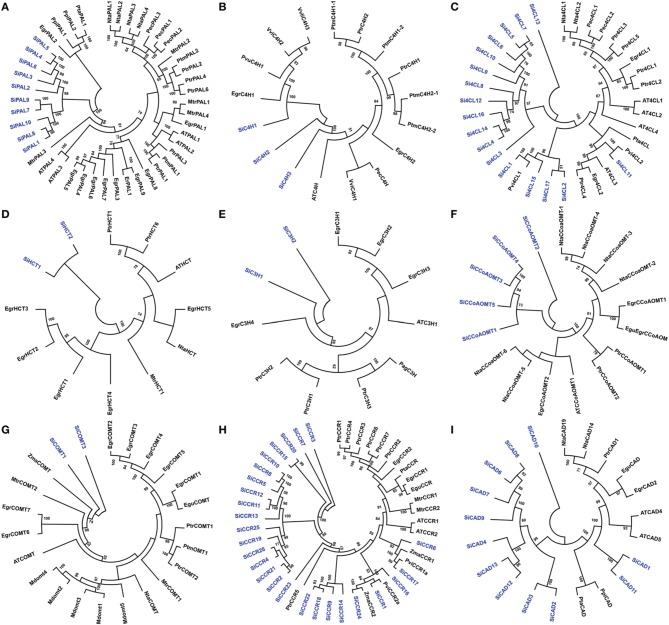
**Unrooted protein phylogenetic trees constructed with (A) PAL, (B) C4H, (C) 4CL, (D) HCT, (E) C3H, (F) CCoAOMT, (G) COMT, (H) CCR, and (I) CAD proteins of *Setaria italica* (Si), *Eucalyptus gunnii* (Egu), *E. grandis* (Egr), *Nicotiana tabacum* (Nta), *Populus trichocarpa* (Ptr), *Pinus pinaster* (Ppi), *Pinus taeda* (Pta), *Medicago truncatula* (Mtr), *Panicum virgatum* (Pvi), *Zea mays* (Zma), *Malus domestica* (Mdom), *Vitis vinifera* (Vvi), *Eucalyptus globulus* (Egl), *Populus alba* x *Populus grandidentata* (Pag), *Petroselinum crispum* (Pec), *Populus tremuloides* (Ptm), *Phaseolus vulgaris* (Pvu), and *Eucalyptus robusta* (Er)**.

Sequence alignment and analysis of SiPAL proteins showed that all the members are almost completely conserved (Supplementary Figure S5). SiPAL2 was found to possess an extended N-terminal sequence of about 135 amino acids, which is unique to this class of protein. A phylogenetic tree constructed with PAL sequences of foxtail millet, eucalyptus, poplar, tobacco, medicago and *Arabidopsis* showed that the SiPAL proteins are phylogenetically divergent from the rest (Figure 3A). Sequence alignment of SiC4H showed that all the members share the conserved P450 superfamily domain and P450-featured motifs, namely, haem-iron binding motif (PFGVGRRSCPG), the T-containing binding pocket motif (AAIETT, the E-R-R-E-R-E-R), for optimal orientation of the enzyme (Supplementary Figure S5). Further, presence of conserved substrate recognition sites (SRSs) of C4H/CYP73A5 enzymes, including SRS1 (SRTRNVVFDIFTGKGQDMVFTVY), SRS2 (LSQSFEYNY), SRS4 (IVENINVAAIETTLWS), and SRS5 (RMAIPLLVPH) was also evidenced (Supplementary Figure S5). Phylogeny of SiC4H along with C4H protein sequences of other organisms showed the grouping of SiC4H1 with C4H1 proteins of eucalyptus and *Phaseolus vulgaris*, whereas SiC4H2 and SiC4H3 were found to be more divergent (Figure 3B).

Si4CL protein sequence alignment showed the presence of 2 highly conserved peptide motifs “box I” (LPYSSGTTGLPKGV; AMP binding signature) and “box II” (GEICIRG), in addition to other conserved regions (Supplementary Figure S5). Phylogeny of 4CL proteins showed grouping of Si4CL1, Si4CL2, Si4CL15, and Si4CL16 with switchgrass (Pvi4CL1), demonstrating their close proximity and similarly, Si4CL11 was found to be grouped with Pvi4CL2, whereas other Si4CL proteins formed their own distinct cluster (Figure 3C). Alignment of SiHCT sequences showed that all the proteins have the conserved motifs for the acyl transferase family, namely “HXXXDG” and “DFGWG” (Supplementary Figure S5). Multiple sequence alignment of SiC3H proteins showed the presence of Cytochrome P450 cysteine heme-iron ligand signature [FW]-[SGNH]-x-[GD]-{F}-[RKHPT]-{P}-C-[LIVMFAP]-[GAD] (Supplementary Figure S5). The conserved motifs including three putative S-adenosyl-L-methionine binding motifs (A, B, and C) and CCoAOMT signature motifs (D, E, F, G, and H) were identified through multiple sequence alignment of SiCCoAOMT proteins (Supplementary Figure S5). Phylogenetic analysis of SiHCT, SiC3H, and SiCCoAOMT proteins with their respective family members of other organisms revealed the dissimilarity of foxtail millet proteins compared to their homologs (Figures 3D–F). In the case of CCoAOMT, SiCCoAOMT2 formed a distinct clade, whereas other SiCCoAOMT members were grouped together in one clade (Figure 3F).

Being truncated proteins, alignment of SiF5H1 with SiF5H2, and SiCOMT2 with SiCOMT1 and SiCOMT3 were not performed (Supplementary Figure S5). Protein sequence alignment between SiCOMT1 and SiCOMT3 did not highlight any consensus motif and their phylogenetic analysis with COMT proteins of other plants showed grouping of SiCOMT with ZmaCOMT of maize (Figure 3G). Sequence alignment of SiCCR proteins revealed that the conserved “KNWYCYGK” motif, catalytic site or the binding site for the cofactor NADPH (Larsen, 2004) has been diversified in foxtail millet (Supplementary Figure S5). Except SiCCR1 and SiCCR24, other SiCCR proteins have at least one amino acid change in this motif, which could be attributed to the substrate affinity of CCR proteins (Pichon et al., 1998). Phylogenetic analysis of SiCCR proteins showed that a maximum of these proteins were clustered in a separate group, whereas few proteins were grouped with CCR proteins of maize, switchgrass and poplar (Figure 3H). Alignment results of SiCAD highlighted a high degree of similarity in conserved domains and binding residues, including Zn-1 binding domain motif GHE(X)_2_G(X)_5_G(X)_2_V, NADP(H) co-substrate-binding motif GXG(X)_2_G (glycine-rich repeat) and Zn-2 metal ion binding motif GD(X)_9, 10_C(X)_2_C(X)_2_C(X)_7_C (Supplementary Figure S5). Phylogenetic tree of SiCAD with CAD proteins of other plant species showed clustering of a maximum of SiCAD proteins in one clade with complete out-grouping of SiCAD10. SiCAD1 and SiCAD11 were found to cluster with poplar CAD proteins (Figure 3I).

### Gene structure of lignocellulose pathway genes

The sequence data of genomic DNA, transcript and CDS along with chromosomal locations of confirmed protein sequences of identified lignocellulose biosynthesis pathway enzymes were retrieved and analyzed for gene size, intron-exon and physical position (Supplementary Tables S2, S6). The size of *SiCesA* genes ranged from 3.1 (*SiCesA8*) to 6.9 kb (*SiCesA9*) and few genes including *SiCesA3, SiCesA7, SiCesA5*, and *SiCesA9* have a maximum of 13 introns, whereas *SiCesA12* was intronless (Supplementary Figure S6). The gene sizes of *SiCsl* ranged from 1.7 (*SiCslA6* and *SiCslE2*) to 6.6 kb (*SiCslA1* and *SiCslF6*), and their gene structure analysis revealed that *SiCsl* genes have up to eight introns (Supplementary Figure S7). The only intronless gene of *SiCsl* superfamily was *SiCslE2*. Among the *SiGsl* gene family members, *SiGsl3* was the smallest gene (3.2 kb), whereas the largest one was *SiGsl4* (17 kb). Interestingly, *SiGsl* genes were evidenced to contain numerous introns. *SiGsl7* has a maximum of 49 introns, whereas *SiGsl2* and *SiGsl3* were intronless (Supplementary Figure S8).

*SiPAL* gene sizes ranged from 2.1 (*SiPAL4*) to 4.6 kb (*SiPAL3*), of which *SiPAL4, SiPAL5*, and *SiPAL6* were intronless, *SiPAL2* has two introns and other *SiPAL* genes have 2 introns each (Supplementary Figure S9). Among the *Si4CL* genes, *Si4CL3* was the smallest gene (2 kb), whereas *Si4CL15* was the largest (6.7 kb). A total of 10 *Si4CL* genes have 5 introns each, while maximum number of introns was found in *Si4CL5* (6 introns). *Si4CL3* has the least number of one intron in its gene (Supplementary Figure S9). The size of *SiCCoAOMT* genes ranged from 0.8 (*SiCCoAOMT4*) to 3 kb (*SiCCoAOMT2*) with a maximum number of introns (7) in *SiCCoAOMT2*. *SiCCoAOMT3* and *SiCCoAOMT4* have one intron each (Supplementary Figure S9). Among the *SiCCR* genes, *SiCCR3* was 1.3 kb in size and though it is the smallest gene of this class, it has eight introns. *SiCCR9* and *SiCCR22* are the largest genes with a size of 5.8 kb and both the genes have 4 introns each. *SiCCR2* has a maximum of 10 introns, while *SiCCR7* is the only intronless gene in this group. The size of *SiCAD* genes ranged from 1.4 (*SiCAD9*) to 4.2 kb (*SiCAD1* and *SiCAD8*), with *SiCAD7, SiCAD8*, and *SiCAD9* having a minimum of 2 introns each whereas *SiCAD5* has a maximum of 6 introns (Supplementary Figure S9).

### Chromosomal location and gene duplication of lignocellulose pathway genes

**Figure 4:**
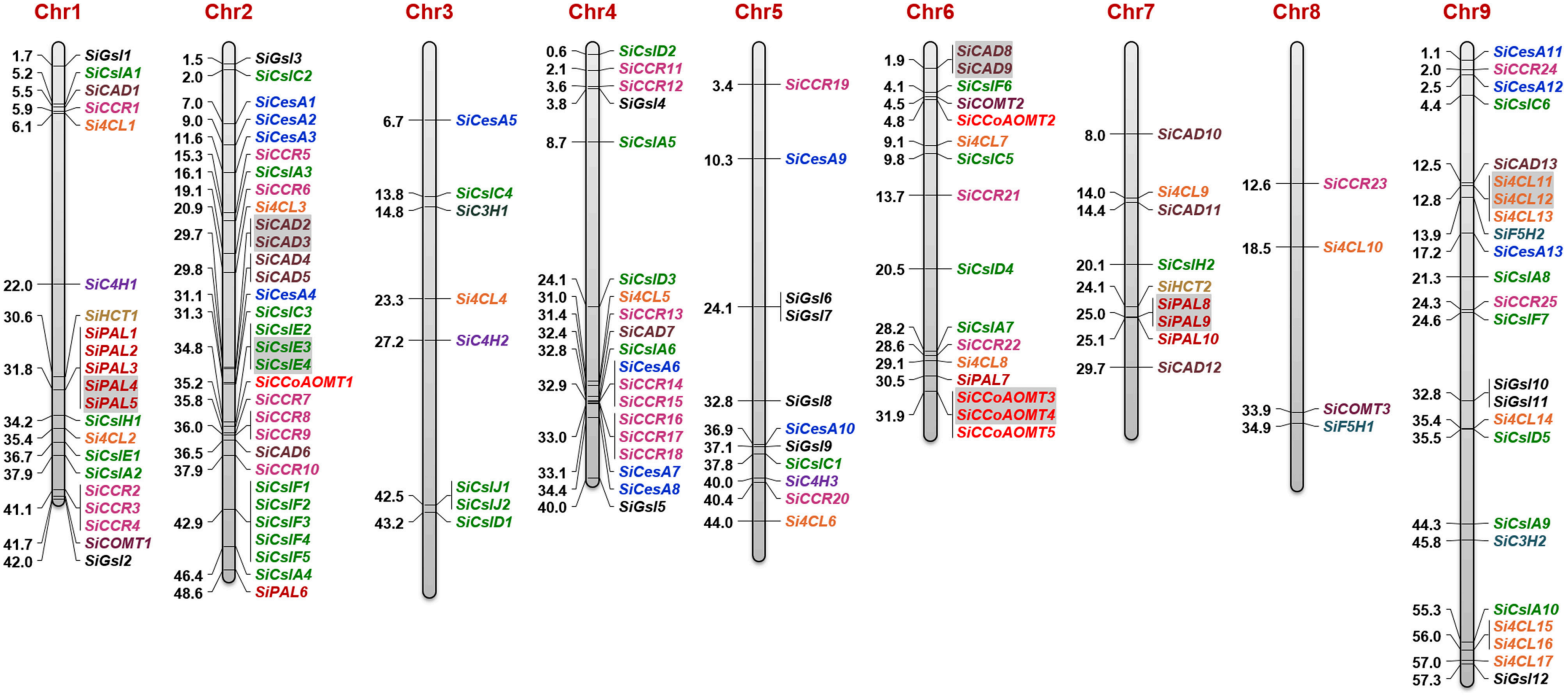
**Physical map showing the chromosomal locations of lignocellulose biosynthesis genes**. Bars represent chromosomes and the numbers at the left corresponds to location (in Mb). Gene IDs are provided in the right. Tandemly duplicated gene pairs are highlighted with gray shade.

The identified secondary cell wall biosynthesis genes were plotted onto the nine chromosomes of foxtail millet to generate the physical map (Figure 4), which showed that the majority of lignocellulose biosynthesis pathway genes (31; ~22%) were present in chromosome 2, followed by chromosome 9 (24 genes; ~17%) and chromosome 1 (21 genes; ~15%), and a minimum of 4 genes (~3%) were mapped on chromosome 8. Expansion of respective gene families within the genome were analyzed by investigating tandem and segmental duplication, which showed that 7 genes underwent tandem duplication, whereas segmental duplication did not occur among the lignocellulose pathway genes (Figure 4). *SiCesA* members were distributed on chromosomes 2 (4 genes), 4 (1), 5 (2), and 9 (3) and none of the genes were evidenced to undergo tandem or segmental duplication. *SiCsl* genes were found to be present in all the chromosomes except chromosome 8, and duplication analysis revealed that *SiCslE3* and *SiCslE4* were tandemly duplicated gene pairs on chromosome 2. *SiGsl* members were distributed on chromosomes 1 (2 genes), 2 (1), 4 (2), 5 (4), and 9 (3) and no duplication pattern in this gene family was observed.

Among the monolignol biosynthesis genes, the majority of *SiPAL* genes were present in chromosome 1 (5) and 7 (3), and interestingly, *SiPAL4* and *SiPAL5* as well as *SiPAL8* and *SiPAL9* were identified to be tandem duplicates. Each of the three *SiC4H* genes were found in chromosome 1, 3, and 5 (Figure 4). A higher number of *Si4CL* genes were present in chromosome 9 (7 genes), of which *Si4CL11* and *Si4CL12* were tandemly duplicated gene pairs. Chromosome 1 and 6 have two *Si4CL* members each and one member each in chromosome 2, 3, 4, 5, 7, and 8. Two members of *SiHCT, SiC3H*, and *SiF5H* as well as three genes of *SiCOMT* were present in chromosome 1, 3, 6, 7, 8, and 9 (Figure 4). Four out of five *SiCCoAOMT* genes were present in chromosome 6 and *SiCCoAOMT1* was mapped on chromosome 2, and duplication analysis revealed that *SiCCoAOMT3* and *SiCCoAOMT4* were tandemly duplicated gene-pairs. Among the *SiCCR* genes, *SiCCR26* could not be mapped due to non-availability of its co-ordinates in Phytozome database. Of the 25 *SiCCR* genes mapped, a maximum of 8 genes were found to be present in chromosome 4 (8), followed by chromosome 2 (6) and 1 (4). Of the 13 *SiCAD* genes, maximum was in chromosome 2 (5) and a minimum of one each in chromosomes 1, 4, and 9. *SiCAD2* and *SiCAD3* on chromosome 2 as well as *SiCAD8* and *SiCAD9* on chromosome 6 were found to be tandemly duplicated gene-pairs (Figure 4).

### Promoter analysis on lignocellulose pathway genes

*In silico* analysis for predicting putative *cis*-regulatory elements showed the presence of universal as well as gene-specific promoter sequences in the upstream of lignocellulose pathway genes (Supplementary Tables S8, S9). A total of 271 *cis*-elements were found in *CesA/Csl* and *Gsl* genes, of which 15 (5.5%) elements, namely ACGTATERD1, ARR1AT, CAATBOX1, CACTFTPPCA1, DOFCOREZM, EBOXBNNAPA, GATABOX, GT1CONSENSUS, GTGANTG10, MYCCONSENSUSAT, NODCON2GM, OSE2ROOTNODULE, POLLEN1LELAT52, WBOXNTERF3, and WRKY71OS were present in all these genes (Supplementary Table S8). Thirty-nine unique *cis*-elements (~14%) which were present in any one gene of *CesA/Csl* and *Gsl* superfamilies were also found, such as ABADESI1 (*SiCslF6*), CEREGLUBOX3PSLEGA (*SiCesA2*), GBOXLERBCS (*SiCslA8*), ZDNAFORMINGATCAB1 (*SiCslA6*), TATCCACHVAL21 (*SiGsl3*), etc. In addition, few promoter sequences were found to be present in all the genes except one or two genes and this includes BIHD1OS (*SiCslC4*), CCAATBOX1 (*SiCslA1, SiCslF4*), CURECORECR (*SiCslC3, SiGsl1*), DPBFCOREDCDC3 (*SiCslC2, SiCslC4*), EECCRCAH1 (*SiGsl5*), MYBCORE (*SiCesA3, SiCslD4*), RAV1AAT (*SiCslD1*), and SORLIP1AT (*SiCesA4, SiGsl8*). Of note, no superfamily specific regulatory elements were identified (Supplementary Table S8).

A total of 293 *cis*-elements were detected in the upstream region of monolignol pathway genes, of which 10 (3.4%) were present in all the genes and 37 (~13%) were unique to any one gene (Supplementary Table S9). The elements which were present in all the genes include ARR1AT, CAATBOX1, CACTFTPPCA1, DOFCOREZM, EBOXBNNAPA, GATABOX, GT1CONSENSUS, GTGANTG10, WBOXNTERF3, and WRKY71OS. Few *cis*-regulatory elements were found to be present in all except one or two genes and it includes ACGTATERD1 (*SiPAL2*), CURECORECR (*SiPAL2, SiPAL10*), and MYBCORE (*SiPAL7, SiCCR16*). Similar to *CesA/Csl* and *Gsl*, no monolignol genes have superfamily specific regulatory elements (Supplementary Table S9).

### MicroRNAs and molecular markers of lignocellulose pathway genes

*In silico* scanning of lignocellulose pathway gene transcripts to identify their targeting miRNAs showed that the transcripts of *SiCslC2, SiGsl10*, and *SiF5H2* could be targeted by the miRNAs sit-miRn29, sit-miR114-npr and sit-miR395b, respectively (Supplementary Table S10). *SiGsl3* was predicted to be targeted by two foxtail millet miRNAs, namely sit-miR156d-1 and sit-miR156d-2. These miRNAs would have a putative role in post-transcriptional gene silencing for regulation of lignocellulose pathway gene expression. Identification of previously reported molecular markers in the genic and regulatory regions of lignocellulose pathway genes revealed the presence of SSR and ILP markers in 34 genes (Supplementary Table S11). Of these, three genes have two and three markers each, and the remaining 28 genes possess single markers. Among the markers, SSRs were found to be predominant (~81%) and the rest are ILPs (~19%).

### Expression profile of lignocellulose pathway genes in tissues and dehydration stress

**Figure 5:**
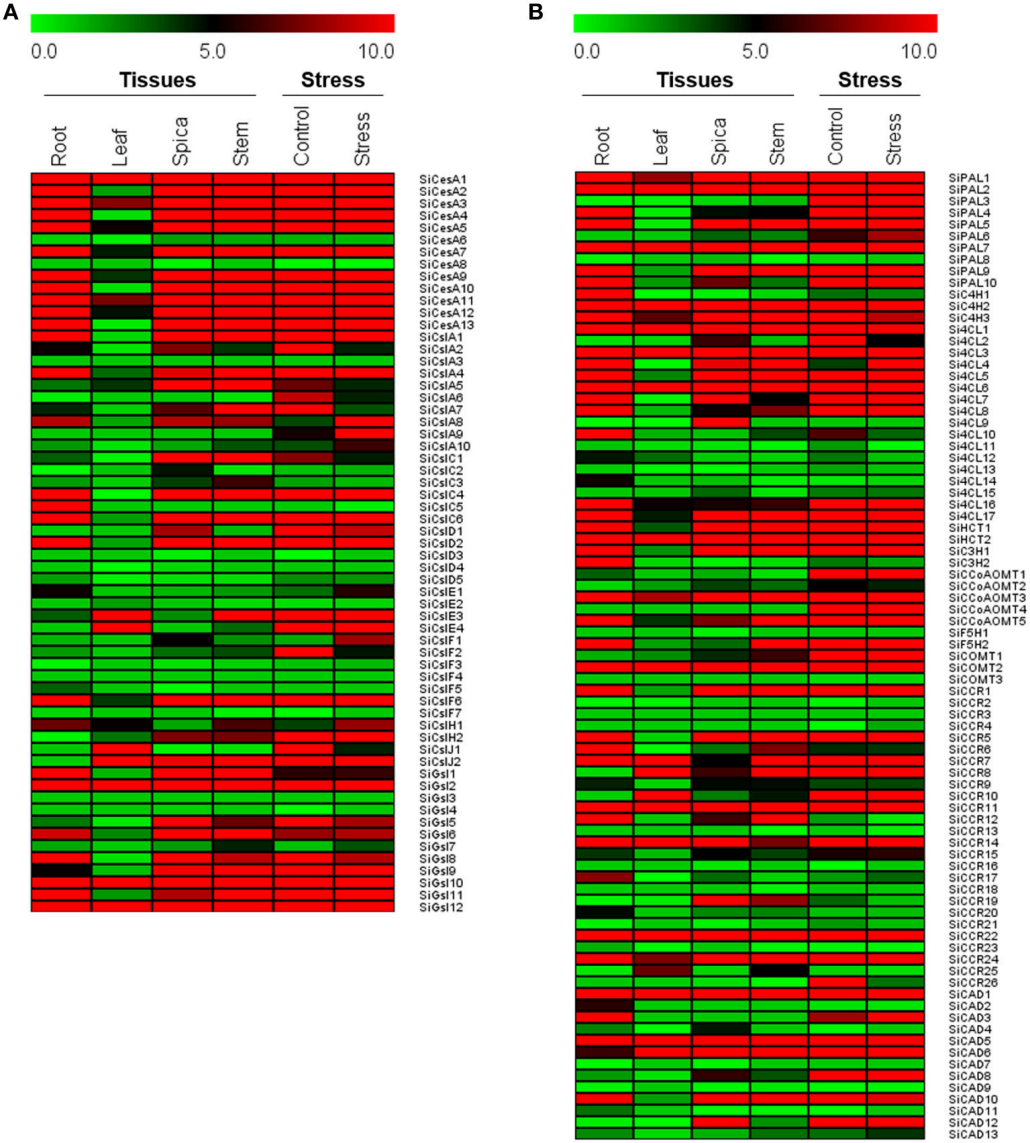
**Heat map showing the expression of (A) cellulose biosynthesis genes, and (B) monolignol biosynthesis genes in four different tissues and dehydration stress library**. The Illumina RNA-seq data were re-analyzed and the heat map was generated. Bar at the top with the values 0.0, 5.0, and 10.0 represent low, intermediate and high expression, respectively.

Expression of all the genes in four tissues and dehydration stress was calculated using RPKM values derived from RNA-seq data. Tissue-specific expression profile showed differential expression pattern of all the genes with relatively lower expression in leaf (Figure 5). In the case of *CesA/Csl* and *Gsl* superfamilies, higher expression of *SiCesA1, SiGsl2, SiGsl10*, and *SiGsl12* was evidenced in all the four tissues when compared to the other members of the same gene family. Tissue-specific higher expression of *SiCslD1* in spica, and *SiCslE4* and *SiCslJ2* in leaf was also observed. Many genes including *SiCesA6, SiCesA8, SiCslA3, SiCslC3, SiGsl3*, and *SiGsl7* were not expressed in these tissues (Figure 5A). Tissue-specific expression profiling of monolignol genes showed higher expression of *SiPAL1, SiPAL2, SiPAL7, SiC4H2, Si4CL1, Si4CL3, Si4CL6, SiHCT2, SiCOMT2, SiCCR11, SiCAD1*, and *SiCAD5* in all the four studied tissues. Tissue-specific higher expression was evidenced with *SIPAL4, Si4CL10*, and *SiCAD3* in root, and *Si4CL9* and *SiCAD12* in spica. Similar to *CesA/Csl* and *Gsl*, monolignol genes also showed a relatively lower expression in leaf tissue (Figure 5B). Expression profiling of all the genes in response to dehydration stress showed almost a uniform expression in both control and stress samples (Figure 5). Comparison of expression patterns between tissues and stress library revealed that the expression of predominant lignocellulose pathway genes was unaltered. Only three genes, namely *SiCslA8, SiCslA9*, and *Si4CL4* showed a higher expression in dehydration stress library compared to control, of which *SiCslA8* and *SiCslA9* were expressed only during stress and not in any of the tissue-specific RNA-Seq libraries. Few genes which were highly expressed in control were observed to be down regulated during stress and this includes *SiCslA5, SiCslA6, SiCslA7, SiCslF2*, and *SiCCR26* (Figure 5).

### Homologous relationships of lignocellulose pathway genes with other grasses

**Figure 6:**
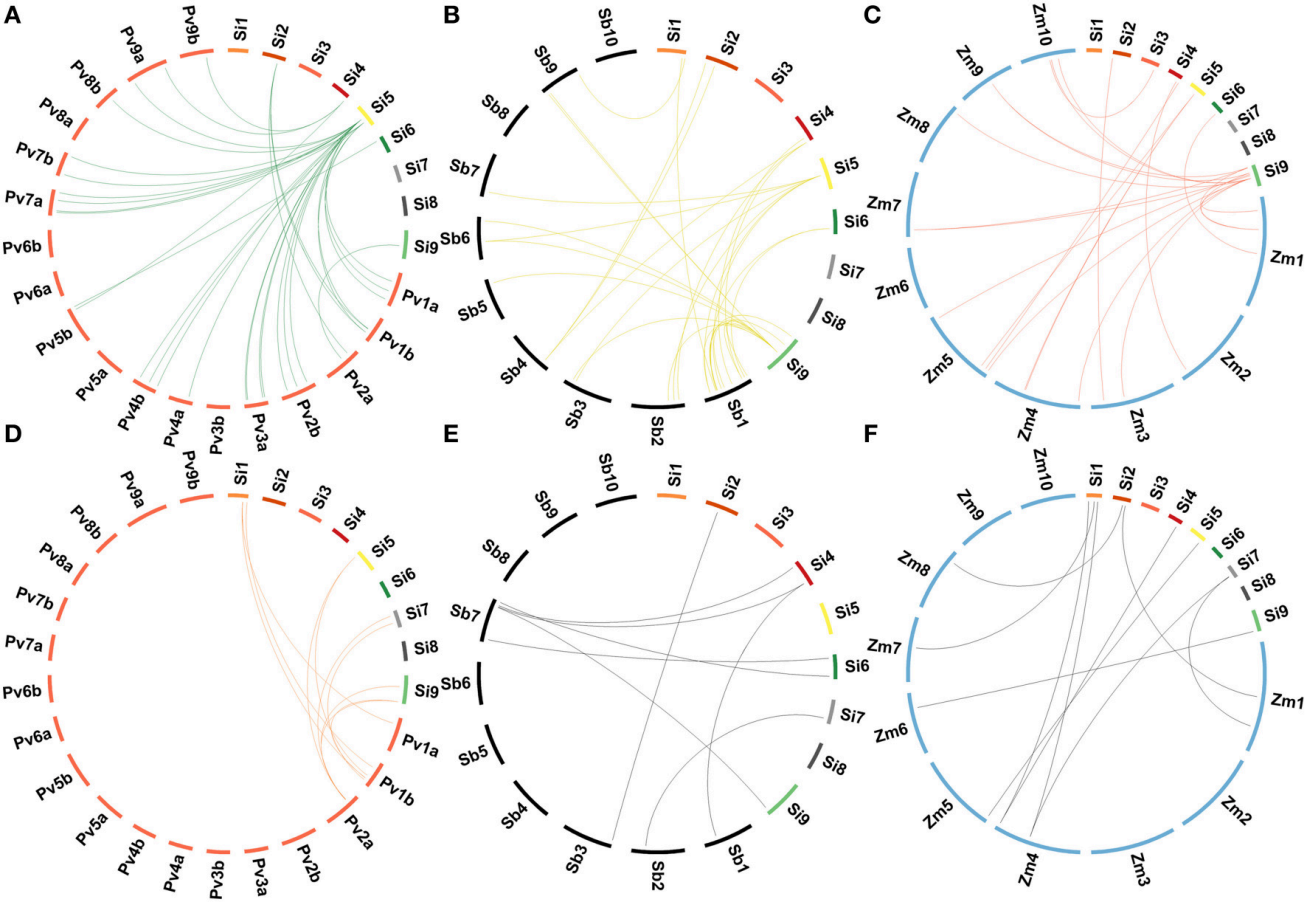
**Comparative genome map showing homologous relationships between *CesA/Csl* and *Gsl* superfamilies of *Setaria italica* and (A) *Panicum virgatum*, (B) *Sorghum bicolor*, (C) *Zea mays*, and between monolignol biosynthesis genes of *Setaria italica* and (D) *Panicum virgatum*, (E) *Sorghum bicolor*, (F) *Zea mays***.

Homologs of foxtail millet lignocellulose pathway genes in sequenced C_4_ panicoid genomes, namely switchgrass (*Panicum virgatum*), sorghum (*Sorghum bicolor*), and maize (*Zea mays*) were derived (Figure 6). A maximum lignocellulose pathway gene-based homology was observed between foxtail millet and switchgrass as 19 genes of foxtail millet showed homology with 60 genes of switchgrass (Supplementary Table S12). Of the 19 foxtail millet genes, six belonged to *SiGsl*, four to *SiCCR*, three each to *SiCsl* and *SiPAL*, and one each to SiHCT, Si4CL and SiCAD. Eighteen foxtail millet genes showed orthologous relationship with 41 sorghum genes, of which *SiGsl11* had a maximum of 11 homologs, followed by *SiGsl7* (7 homologs) and *SiGsl5* and *SiCCR17* (3 homologs each) (Supplementary Table S13). In the case of foxtail millet-maize homology, 26 foxtail millet genes showed homologous relationship with 38 maize genes (Supplementary Table S14). Among the foxtail millet genes, *SiGsl* had a maximum of 7 homologs in maize, followed by *SiGsl7* (3 homologs).

Among the lignocellulose pathway proteins, CADs and COMTs were well characterized as they play key role in secondary cell wall lignification (Saballos et al., 2009; Saathoff et al., 2011a,b, 2012; Sattler et al., 2012; Trabucco et al., 2013). Sequence analysis of these proteins in several grasses identified the presence of conserved motifs in few members, which distinguish them as lignifying proteins from the rest of non-lignifying proteins. Lignifying CADs possess additional 12 amino acids T^49^, Q^53^, L^58^, M^60^, C^95^, W^119^, V^276^, P^286^, M^289^, L^290^, F^299^, and I^300^, which are involved in substrate recognition and binding (Youn et al., 2006). Of the 13 SiCAD proteins, SiCAD11 contains 11 of 12 conserved amino acid residues. Of note, the active substrate-binding residues, W119 and F298, which determine specificity for aromatic alcohols and, the NADP(H) binding site, S212, were present in SiCAD11. Sequence-based homology analysis showed higher percentage of identity between SiCAD11 and lignifying CADs of other grasses namely switchgrass (Pavir.J34526; 91%), sorghum (Sobic.006G211900; 89%) and maize (GRMZM5G844562; 85%). Similarly, the conserved amino acids M^130^, N^131^, L^136^, A^162^, H^166^, F^176^, M^180^, H^183^, I^319^, M^320^, and N^324^, which function in substrate-binding and positioning in COMTs (Sattler et al., 2012; Trabucco et al., 2013) are found to be present in SiCOMT02 of foxtail millet. Sequence-based homology with SiCOMT02 showed high percent identity to sorghum (Sobic.007G047300; 94%), switchgrass (Pavir.Fa01907; 85%), and maize (AC196475.3; 89%).

### Duplication and divergence of lignocellulose pathway genes

The number of non-synonymous substitutions per non-synonymous site (Ka) and synonymous substitutions per synonymous site (Ks) was calculated for paralogous as well as homologous gene pairs and Ka/Ks ratio along with time of divergence (in million years ago; mya) were derived. The ratio of Ka to Ks for tandemly duplicated gene-pairs ranged from 0.09 to 0.18 with an average value of 0.13, which suggested that these genes were under strong positive purifying selection (Ka/Ks > 1) and the duplication event was predicted to occur around 25 mya (Supplementary Table S15). In the case of Ka/Ks ratio of homologous gene-pairs, it was maximum between foxtail millet-switchgrass (average Ka/Ks = 0.91; Supplementary Table S12), whereas foxtail millet-sorghum and foxtail millet-maize homologs showed an average ratio of 0.19 (Supplementary Tables S13, S14). Since these values were less than 1, it signifies the intense positive selective pressure acted on respective protein-coding genes. The time of divergence between foxtail millet and switchgrass was predicted to occur around 4.7 mya, whereas the divergence of foxtail millet-sorghum and foxtail millet-maize occurred around 27 mya. This demonstrates that duplication and divergence have played a key role in shaping the lignocellulose pathway multigene families in foxtail millet and other C_4_ grass genomes.

### Expression profile of candidate genes during stress and hormone treatments

**Figure 7:**
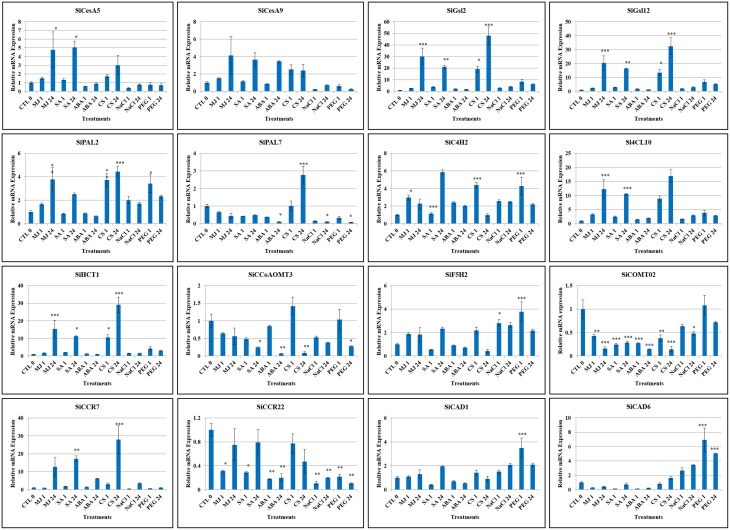
**Relative expression of candidate lignocellulose biosynthesis genes analyzed using qRT-PCR under dehydration (PEG), salinity (NaCl) and cold stress (CS) as well as abscisic acid (ABA), salicylic acid (SA) and methyl jasmonate (MJ) treatments for 0 (Control: CTL), 1 and 24 h. *Act2* was used as an internal control to normalize the data**. The error bars representing standard deviation were calculated based on three technical replicates for biological triplicates. Statistical analysis between treatment and control using Tukey-Kramer multiple comparisons test has been performed and the differences in the effects of stress treatments in all the genes were considered statistically significant at ^*^P < 0.05, ^**^P < 0.01, ^***^P < 0.001.

Expression patterns of sixteen candidate lignocellulose biosynthesis genes, namely *SiCesA5, SiCesA9, SiGsl2, SiGsl12, Si4CL10, SiPAL2, SiPAL7, SiC4H2, SiHCT1, SiCCoAOMT3, SiF5H2, SiCOMT2, SiCCR7, SiCCR22, SiCAD1*, and *SiCAD6* in response to stress (dehydration, salinity, cold) and hormone (abscisic acid, salicylic acid, methyl jasmonate) treatments was performed at two time points (1 h, early; 24 h, late). These candidates were chosen based on; (i) expression profiles deduced *in silico* using RNA-seq data, (ii) representing the nine chromosomes of foxtail millet, and (iii) their function in secondary cell wall formation such as *SiCOMT2* in lignification Overall, the study demonstrated differential expression pattern of these genes during stress and hormone treatments except *SiCCR22* which was found to be down-regulated under all conditions (Figure 7). *SiGsl2* and *SiGsl12* were found to be highly expressed during all the three stress conditions, whereas *SiCAD6* was up-regulated during both salinity and dehydration stress. Dehydration stress has been observed to induce the expression of all the genes except *SiCCoAOMT3, SiCOMT2, SiCesA5, SiCCR22, SiPAL7, SiCCR7*, and *SiCesA9*, though the degree of expression varied between the genes. Salinity stress showed an induction in expression of *SiC4H2, SiCAD6, SiF5H2, SiGsl12*, and *SiGsl2*, while *SiPAL2* was induced during early salt stress and *SiCAD01, Si4CL10*, and *SiCCR7* were found to be up-regulated in late phase salinity stress, thus suggesting a significant higher expression among the members of *SiGsl* and *SiCAD* family. Significant up-regulation of *SiGsl2, SiGsl12, Si4CL10, SiHCT1*, and *SiCCR7* during cold stress suggests the putative involvement of these genes in strengthening the cell wall for tolerance to low temperature. Higher expression of these genes was also found during both early and late phases of treatment with salicylic acid and methyl jasmonate. Differential expression of candidate genes was observed during the treatment of all the hormones except abscisic acid, which showed no effect on the expression of majority of candidate genes except *SiGsl2*, which was induced at early phase of ABA treatment, *SiCCR7* and *SiCes9*, which were induced at late phase of ABA treatment, and *SiC4H2*, which was induced at both the phases of ABA treatment. In addition, expression of *SiCCoAOMT3, SiCOMT2, SiCCR22, SiPAL7, SiCAD1*, and *SiCAD6* was found to be down-regulated during hormone treatments, while *SiF5H2* was up-regulated only under late phase of salicylic acid treatment.

## Discussion

Cellulose, hemicelluloses and lignin constitute the complex polymeric structure of secondary cell wall and the lignocellulose biosynthesis pathway involves the action of *cellulose synthase* (*CesA*), *cellulose synthase-like* (*Csl*), *glucan synthase-like* (*Gsl*), *phenylalanine ammonia lyase* (*PAL*), *trans-cinnamate 4-hydroxylase* (*C4H*), *4-coumarate CoA ligase* (*4CL*), *hydroxycinnamoyl CoA:shikimate/quinate hydroxycinnamoyl transferase* (*HCT*), *p-coumaroyl shikimate 3*′*-hydroxylase* (*C3H*), *caffeoyl CoA 3-O-methyltransferase* (*CCoAOMT*), *ferulate 5-hydroxylase* (*F5H*), *caffeic acid O-methyltransferase* (*COMT*), *cinnamoyl CoA reductase* (*CCR*), and *cinnamyl alcohol dehydrogenase* (*CAD*) genes, which are well studied in several crop plants as well as trees for understanding and improving biofuel traits (Zhong and Ye, 2015). In the present study, all these gene families in foxtail millet were systematically identified and characterized using *in silico* approaches, and expression profiling of chosen genes was performed in response to several stress as well as hormonal treatments for identifying target genes for functional characterization.

A total of 13 *CesA* and 36 *Csl* genes were identified in foxtail millet, and all the SiCesA proteins were found to possess the characteristic cellulose synthase (CS) domain and 12 SiCesA had an additional zinc finger (ZF) structure. Similarly, 11 CesA proteins have been reported in rice, of which 9 contained both CS and ZF domain, and 2 lacked ZF domain (Wang et al., 2010). Role of CesA proteins in cellulose biosynthesis in both primary and secondary cell walls has been well dissected in *Arabidopsis*. In this plant, 10 *CesA* genes have been identified (Richmond and Somerville, 2000), of which *AtCesA1, AtCesA3*, and *AtCesA6* were reported to be involved in primary cell wall cellulose synthesis (Persson et al., 2007), *AtCesA4, AtCesA7*, and *AtCesA8* in secondary cell wall development, and *AtCesA2, AtCesA5, AtCesA9*, and *AtCesA10* in tissue-specific cellulose biosynthesis processes (Gardiner et al., 2003; Taylor et al., 2003). Recent functional characterization of AtCesA proteins led to the identification of unidirectional movement of these protein complexes in seed coat epidermal cells, which deposit cellulose that are involved in mucilage extrusion, adherence and ray formation (Griffiths et al., 2015). In flax (*Linum usitatissimum*), 14 distinct *CesA* genes were identified and were targeted for silencing using virus-induced gene silencing (VIGS) approach, which showed impacts on outer-stem tissue organization and secondary cell wall formation (Chantreau et al., 2015). A genome-wide association study of single nucleotide polymorphisms (SNPs) developed through re-sequencing of diverse chickpea accessions revealed a superior haplotype and favorable natural allelic variants in the upstream regulatory region of a *CesA* gene, denoted as *Ca_Kabuli_CesA3* (Kujur et al., 2015). Interestingly, up-regulation of this superior gene haplotype resulted in higher transcript expression of *Ca_Kabuli_CesA3* gene in pollen and pod of high pod/seed number chickpea accession, thus resulting in enhanced accumulation of cellulose (Kujur et al., 2015). The specific allelic variant caused cellulose changes specifically in pollen tubes of chickpea and therefore, investigating the homologous gene of foxtail millet identified in the present study will provide novel clues on its role, which could be manipulated for achieving greater biomass yield and bioconversion efficiency.

Physical map of *SiCesA* genes showed their distribution in chromosomes 2, 3, 4, 5, and 9, with a maximum of 4 genes in chromosome 4 and minimum of one gene in chromosome 3 (Figure 4). Extension of gene families is attributed to the occurrence of three major duplication mechanisms, namely segmental, tandem and retroposition (Cannon et al., 2004). However, none of these duplications were found to be involved in the expansion of *SiCesA* genes as revealed through MCScanX analysis though both tandem and segmental duplication events were reported in *OsCesA* family (Wang et al., 2010). Being a member of glycosyltransferase 2 (GT2) family, CesA proteins have the conserved “DXD, D, QXXRW” motif (Somerville et al., 2004) and conforming to this, all the *SiCesA* proteins except SiCesA8 have a “DCD, D, QVLRW” consensus sequence, whereas SiCesA8 had a unique “DYD, D” sequence and the motif “QXXRW” was absent. Similar sequence variations have also been reported by Wang et al. (2010) in rice. Studies on *CesA* gene family in crop plants have revealed the presence of a large family of cellulose synthase-like (*Csl*) genes with sequence similarity to *CesA* (Richmond and Somerville, 2000), and these genes are shown to be involved in biosynthesis of hemicelluloses (Yin et al., 2009). Similar to CesA, Csl proteins also belong to GT2 family and possess the conserved “DXD, D, QXXRW” motif (Somerville et al., 2004). In foxtail millet, 36 *Csl* genes were identified and categorized as *CslA, CslC, CslD, CslE, CslF, CslH*, and *CslJ* in accordance to the classification followed by Wang et al. (2010) in rice. Interestingly, 2 *CslJ* genes were identified in foxtail millet, which were reported to be specific to cereals though they are not present in rice and *Brachypodium* (Fincher, 2009). Domain analysis has shown the presence of GT2 domains in all SiCslA and SiCslC proteins, whereas other SiCsl possess CS domain. Similar reports in *Arabidopsis* and rice have shown the presence of characteristic GT2 domain in CslA and CslC proteins (Yin et al., 2009; Wang et al., 2010). Studies have shown that CslA and CslC subgroups are the most divergent proteins, which have evolved through duplication and divergence from a common ancestral gene (Yin et al., 2009; Del Bem and Vincentz, 2010), and therefore share similar structural and physicochemical properties (Youngs et al., 2007). Nevertheless, membrane topology and enzymatic function of these proteins are contrastingly different (Davis et al., 2010; Liepman and Cavalier, 2012). In addition, predominant SiCslD family proteins have an additional RING/Ubox like zinc-binding domain, which contains a C3HC4 motif capable of binding to zinc cations.

Molecular processes and biological functions of *Csl* genes have been less explored when compared to *CesA* genes though Csl proteins are equally important in cell structuring. Numerous reports have supported the involvement of CslA protein in the synthesis of 1,4-β-mannan and glucomannan backbones (Dhugga et al., 2004; Liepman et al., 2005; Suzuki et al., 2006; Goubet et al., 2009; Gille et al., 2011) and heterologous expression of *CslA* genes has shown the activity of single enzyme in integrating mannose and glucose into glcomannan chains (Suzuki et al., 2006; Liepman et al., 2007; Gille et al., 2011). Similarly, *CslC* genes encode for xyloglucan glucan synthase, which are involved in xyloglucan biosynthesis (Cocuron et al., 2007). Heterologous expression of *AtCslC4* in *Pichia pastoris* produced soluble 1,4-β-glucans with a low degree of polymerization, whereas expression of *AtCslC4* along with *AtXXT1* (xyloglucan xylosyltransferase) produced insoluble 1,4-β-glucans with a higher degree of polymerization suggesting the cooperative action of both the enzymes in xyloglucan biosynthesis (Liepman and Cavalier, 2012). Though CslD proteins were speculated to be involved in xylan and homogalacturonan synthesis (Hamann et al., 2004; Bernal et al., 2008a,b; Li et al., 2009), *Arabidopsis csld* mutants have been shown to possess severe phenotypic defects including deformed root hairs (*csld2*; Bernal et al., 2008b), root hairs burst (*csld3*; Bernal et al., 2008b), defective growth of pollen tube (*csld1* and *csld4*; Bernal et al., 2008b; Wang et al., 2011) and reduced plant growth (*csld5*; Bernal et al., 2008a). These reports suggest the role of CslD in normal growth and development of plants beyond their function in xylan and homogalacturonan synthesis. The present study identified 4 *SiCslE* genes, whose characterization has not been performed yet in any crop species. One *CslE* gene in *Arabidopsis* and two in rice were reported to date. *CslF* family of genes were considered to be present among grass species and they regulate the synthesis of mixed-linkage glucan (β-1,3; 1,4, glucan) (Hazen et al., 2002; Burton et al., 2006). Mutation of barley *CslF6* gene resulted in reduction of (1,3;1,4)-β-D-Glucan and had an impact on chemical composition of barley grains (Hu et al., 2014), whereas overexpression of this gene in *Nicotiana benthamiana* led to accumulation of (1,3;1,4)-β-D-Glucan (Wong et al., 2015). Recently, Jin et al. (2015) has demonstrated the role of *OsCslF6* in affecting phosphate accumulation altering the level of carbon metabolism in rice. Similar to *CslF, CslH* and *CslJ* are also grass-specific gene family involved in deposition of (1,3;1,4)-β-D-Glucan (Doblin et al., 2009; Yin et al., 2009, 2014). In the present study, two genes each belonging to *CslH* and *CslJ* family were identified.

Similar to CesA/Csl, glucan synthase-like protein (Gsl) family are also involved in polysaccharide biosynthesis, particularly in synthesis 1,3-β-D-glucan callose (Li et al., 1999). Calloses are deposited in developing cell walls of fiber cells, seed hairs and plasmodesmatal canals. Moreover, deposition of callose is also reported in response to pathogen invasion (Muthamilarasan and Prasad, 2013) and abiotic stress including desiccation, wounding and metal toxicity (Stone and Clarke, 1992). In spite of the importance of *Gsl* genes, limited studies have been performed on elucidating the molecular role of these genes and their respective proteins. In *Arabidopsis*, 12 *Gsl* genes have been identified (https://cellwall.genomics.purdue.edu/intro/index.html) and mutating *AtGSL5* has been found to confer resistance to powdery mildew infection (Nishimura et al., 2003). A similar report by Jacobs et al. (2003) has also shown that silencing of *AtGsl5* enhances the resistance of silenced lines to *Sphaerotheca fusca, Golovinomyces orontii*, and *Blumeria graminis*. In contrast to the role of callose in acting as a physical barrier to prevent pathogen invasion, the reports by Nishimura et al. (2003) and Jacobs et al. (2003) have demonstrated the resistance of *Arabidopsis* to pathogens in the absence of callose. These reports have proved the importance to study the molecular and physiological roles of Gsl proteins in response to biotic as well as abiotic stress, and the present investigation has identified 12 *SiGsl* genes which could serve as interesting candidates for functional characterization as foxtail millet is tolerant to environmental stresses.

In the case of monolignol biosynthesis, ten key enzymes namely PAL, C4H, 4CL, HCT, C3H, CCoAOMT, F5H, COMT, CCR, and CAD have been identified and characterized in the present study. Through systematic analysis, 10, 3, 17, 2, 2, 5, 2, 3, 26, and 13 proteins belonging to PAL, C4H, 4CL, HCT, C3H, CCoAOMT, F5H, COMT, CCR, and CAD families, respectively were identified (Supplementary Table S6). These numbers compared with the genes reported in *Arabidopsis*, poplar and eucalyptus has shown that foxtail millet has higher number of *PAL* genes (10) whereas other three organisms have 4, 5, and 9 genes, respectively (Raes et al., 2003; Shi et al., 2010; Carocha et al., 2015). Both foxtail millet and poplar have 2 *C4H* and 17 *4CL* genes, whereas *Arabidopsis* and eucalyptus have lesser number of *C4H* and *4CL* genes. Of note, foxtail millet has a maximum of 26 *CCR* genes, while *Arabidopsis* has 7 and eucalyptus has 2 genes (Raes et al., 2003; Shi et al., 2010; Carocha et al., 2015). The identified monolignol biosynthesis genes were distributed in all the nine chromosomes of foxtail millet, of which two gene-pairs each of *SiPAL* (*SiPAL4-SiPAL5*; *SiPAL8-SiPAL9*) and *SiCAD* (*SiCAD2*-*SiCAD3*; *SiCAD8*-*SiCAD9*), and one pair each of *Si4CL* (*Si4CL11-Si4CL12*) and *SiCCoAOMT* (*SiCCoAOMT3*-*SiCCoAOMT4*) were identified to be tandemly duplicated (Figure 4). Phylogenetic analysis of foxtail millet monolignol biosynthesis proteins with *bona fide* proteins of eucalyptus, tobacco, poplar, *Arabidopsis*, maize, medicago and grape revealed that predominant proteins of foxtail millet are highly divergent (Figure 3).

Furthermore, promoter analysis has been performed for foxtail millet lignocellulose biosynthesis genes, which revealed the presence of diverse *cis*-regulatory elements that fall under the following categories; (i) *cis*-elements which are universally present in all the gene family members, (ii) *cis*-elements which are present in all the gene family members except one gene, and (iii) *cis*-element which is unique to any one gene of its corresponding gene family (Supplementary Tables S8, S9). These data suggest the transcriptional control of cell wall genes by the action of network of transcription factors. This would assist in understanding gene regulatory mechanism controlling the expression of lignocellulose genes and fine tuning them to achieve the optimal pattern of secondary cell-wall deposition. Since gene expression is also regulated at post-transcriptional level through miRNAs, the present study also identified foxtail millet miRNAs which target the transcripts of lignocellulose biosynthesis genes (Supplementary Table S10). Moreover, different kinds of molecular markers including SSRs, eSSRs, and ILPs present in both upstream and genic region of lignocellulose biosynthesis genes have been identified (Supplementary Table S11), which could be useful for conducting genomics-assisted breeding for biofuel traits in foxtail millet. *In silico* expression profiles of all the lignocellulose biosynthesis genes in four tissues as well as dehydration library revealed the differential expression of these genes in these tissues and during stress, thus signifying their putative involvement in biological functions other than cell wall structuring. This is supported by the reports on mutants of studied genes in *Arabidopsis* and other plants in which severe phenotypic defects have been observed.

In addition to being potential targets for biofuel traits, the lignocellulose biosynthesis genes have also been reported to play vital role in abiotic stress responses. Chen et al. (2005) have shown that *Arabidopsis CesA8* mutants accumulate increased levels of ABA, proline and sugars, and express higher levels of stress-related genes, and thus possess enhanced tolerance to drought and osmotic stress. Considering this, Guerriero et al. (2014) analyzed the expression of nine putative *CesA* genes in response to cold, heat and salt stress in *Medicago sativa* and identified a salt/heat-induced and a cold/heat-repressed group of genes, which suggest the putative involvement of cellulose synthases in conferring abiotic stress tolerance. Similar to *CesA* genes, *Csl* genes have also been shown to participate in stress responsive machinery. Characterization of *the salt-overly sensitive6* allele of *AtCslD5* has demonstrated reactive oxygen species-based signaling mechanism in response to osmotic stress in *Arabidopsis* (Zhu et al., 2010). Similarly, accumulation of callose in response to environmental stimuli through overexpression of *Gsl* genes has been extensively studied (Nedukha, 2015). Stass and Horst (2009) have reported the production of abiotic stress-induced callose in all the plants through a highly conserved signaling pathway. Lignification has also been reported to be induced during abiotic stresses (Moura et al., 2010). In view of these, expression profiling of candidate genes in response to dehydration, salinity and cold stress as well as ABA, SA, and MeJA treatments was performed, which showed significant higher expression of *SiGsl2* and *SiGsl12* in all the stress conditions. Few genes including *SiCAD6, SiC4H2, SiPAL2, SiF5H2, Si4CL10, SiHCT1*, and *SiCCR7* were evidenced to be up-regulated either at early or late or both the phases of stresses. Similarly, differential expression patterns were observed for all the genes during hormone treatments and of note, ABA treatment has no significant impact on the expression of the majority of genes.

Noteworthy, the expression profiles of candidate lignocellulose biosynthesis genes were in correlation with the *cis*-regulatory elements present in the promoter regions of respective genes. The genes which are up-regulated during dehydration and salinity stress including *SiGsl2, SiGSl12, SiPAL2, SiC4H2, Si4Cl10, SiF5H2, SiHCT1, SiCAD1*, and *SiCAD6* have one or more “response to dehydration stress” *cis-*motifs ABRELATERD1, ACGTATERD1 and MYCATRD22 in their promoter regions (Vandepoele et al., 2009; Yan et al., 2014). Similarly, *SiGsl2, SiGSl12* and *Si4Cl10* that showed higher expression under cold stress have CACGT motif, which was reported to be responsible for response to cold stress (Vandepoele et al., 2009). In case of hormonal treatments, methyl jasmonate responsive *cis-*element BOXLCOREDCPAL (Yan et al., 2014) was found in the promoter regions of *SiCesA5, SiGsl2, SiGSl12, Si4Cl10, SiPAL2, SiC4H2*, and *SiCCR7*. These genes showed significant up-regulation at either early or late or both the phases of methyl jasmonate treatment. Similarly, ABA-responsive genes such as *SiC4H2, SiCCR7, SiGsl2*, and *SiCesA9* have both MYCCONSENSUSAT and MYCATRD22 *cis*-motifs, which have been reported to be MYC recognition site in the promoter of dehydration responsive *rd22* gene which in turn was ABA-dependent (Yan et al., 2014), suggesting that these genes were activated in response to ABA. Thus the present study demonstrates that the interaction of *cis*-elements and transcription factors has resulted in differential gene expression through activation or repression respective genes in response to various environmental stresses and hormone treatments (Lee et al., 2002; Benitez et al., 2013). The findings and potential correlation between the cis-elements to response to a specific elicitor condition are indirect. It is possible that they are linked, but such primary evidence is not provided here. It is also not known if there were any changes to cell walls in the plants used for expression analyses. Altogether, the present investigation suggests the putative involvement of these genes in strengthening the cell wall for tolerance to abiotic stresses, and they could serve as potential candidates for further functional characterization.

## Conclusions

The present study has identified the genes belonging to *CesA/Csl, Gsl, PAL, C4H, 4CL, HCT, C3H, CCoAOMT, F5H, COMT, CCR*, and *CAD* superfamilies in foxtail millet and the genes were mapped onto nine chromosomes. *In silico* analyses of putative protein properties and gene structures revealed diverse characteristic features of these proteins and their gene duplication analysis showed that few gene family members underwent tandem duplication. Phylogenetic analysis of respective proteins demonstrated that except CesA/Csl and Gsl superfamily, the monolignol biosynthesis proteins are highly diverse. Promoter analysis showed the presence of various unique and common *cis*-regulatory elements in the upstream of lignocellulose biosynthesis genes and potential miRNAs of foxtail millet were identified to target few genes for post-transcriptional gene silencing. In addition, three types of molecular markers were found in lignocellulose biosynthesis genes, which could be used in genomics-assisted breeding. Comparative genome mapping of foxtail millet lignocellulose biosynthesis genes with the sequenced C_4_ panicoid genomes revealed higher homology with switchgrass, followed by sorghum and maize. Evolutionary analysis showed that both paralogous and homologous gene-pairs underwent intense positive purifying selection, and duplication occurred ~25 mya, whereas divergence of foxtail millet and switchgrass occurred ~4 mya. Similarly, divergence of foxtail millet from sorghum and maize was predicted to occur ~27 mya. *In silico* expression analysis of all the identified genes in four tissues and dehydration stress library of foxtail millet revealed their differential expression pattern, and also suggested the putative biological function of these genes in processes other than cell wall biosynthesis. Expression profiling of candidate genes in response to dehydration, salinity and cold stress along with ABA, SA and MeJA treatments supported the differential expression of these genes with significant higher expression of *SiGsl12, SiHCT1*, and *SiCAD6* genes. The results suggested that these genes could be used as potential candidates for functional characterization for biofuel traits. Though similar studies have already been completed in switchgrass, sorghum and maize, the present study conducted in biofuel model foxtail millet would facilitate improving the crop for efficient biofuel production.

## Author contributions

MP conceived and designed the experiments. MM, YK, JJ, SS, CL performed the experiments. MM, CL, MP analyzed the results. MM, MP wrote the manuscript. MP approved the final version of the manuscript.

## Funding

Research on foxtail millet genomics at MP's laboratory is funded by the Core Grant of National Institute of Plant Genome Research, New Delhi, India.

### Conflict of interest statement

The authors declare that the research was conducted in the absence of any commercial or financial relationships that could be construed as a potential conflict of interest.

## Supplementary material

Supplementary Figure S1 **Multiple sequence alignment of SiCesA proteins**.

Supplementary Figure S2 **Multiple sequence alignment of SiCsl proteins**.

Supplementary Figure S3 **Multiple sequence alignment of SiGsl proteins**.

Supplementary Figure S4 **Prediction of transmembrane domains in the SiGsl proteins**. Red line represents transmembrane, blue line represents inside and pink line represents outside orientation.

Supplementary Figure S5 **Multiple sequence alignment of monolignol biosynthesis proteins**.

Supplementary Figure S6 **Gene structure of *SiCesA* genes**.

Supplementary Figure S7 **Gene structure of *SiCsl* genes**.

Supplementary Figure S8 **Gene structure of *SiGsl* genes**.

Supplementary Figure S9 **Gene structure of monolignol biosynthesis genes**.

Supplementary Table S1 **Details of primers used in qRT-PCR analysis**.

Supplementary Table S2 **Details of *SiCesA/Csl* and *SiGsl* superfamily genes of foxtail millet**.

Supplementary Table S3 **Details of various domains present in SiCesA proteins**.

Supplementary Table S4 **Details of various domains present in SiCsl proteins**.

Supplementary Table S5 **Details of various domains present in SiGsl proteins**.

Supplementary Table S6 **Details of monolignol biosynthesis pathway genes of foxtail millet**.

Supplementary Table S7 **Details of various domains present in monolignol biosynthesis pathway proteins**.

Supplementary Table S8 **Summary of cis-regulatory elements present in *SiCesA/Csl* and *SiGsl* superfamily genes**.

Supplementary Table S9 **Summary of cis-regulatory elements present in monolignol biosynthesis pathway genes**.

Supplementary Table S10 **Details of foxtail millet miRNAs identified to target the transcripts of lignocellulose pathway genes**.

Supplementary Table S11 **Summary of molecular markers present in lignocellulose pathway genes**.

Supplementary Table S12 **The Ka/Ks ratios and estimated divergence time for homologous lignocellulose pathway proteins between *Setaria italica* and *Panicum virgatum***.

Supplementary Table S13 **The Ka/Ks ratios and estimated divergence time for homologous lignocellulose pathway proteins between *Setaria italica* and *Sorghum bicolor***.

Supplementary Table S14 **The Ka/Ks ratios and estimated divergence time for homologous lignocellulose pathway proteins between *Setaria italica* and *Zea mays***.

Supplementary Table S15 **The Ka/Ks ratios and estimated divergence time for tandemly duplicated lignocellulose pathway proteins**.
