# Supplementary material for: Integrative analysis and expression profiling of secondary cell wall genes in C4 biofuel model Setaria italica reveals targets for lignocellulose bioengineering
Source: Front Plant Sci. 2015 Nov 4;6:965. doi: 10.3389/fpls.2015.00965 (PMC4631826; doi:10.3389/fpls.2015.00965)

Supplementary Figure S1

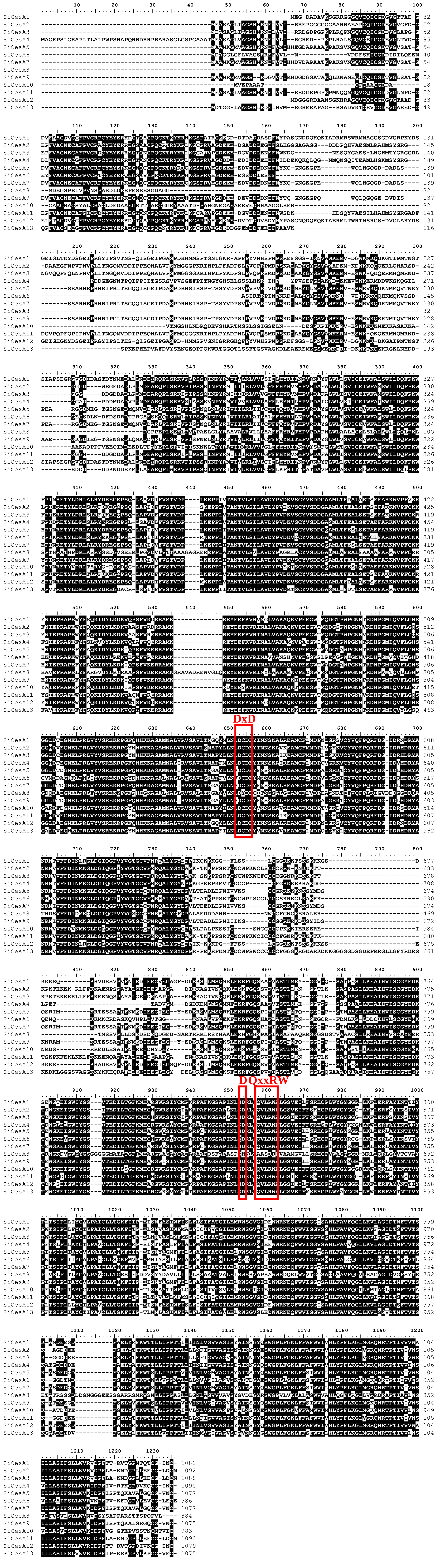

Supplement: Supplementary Figure S1 — Multiple sequence alignment of SiCesA proteins. [file Image1.PDF]
