# Supplementary material for: Integrative analysis and expression profiling of secondary cell wall genes in C4 biofuel model Setaria italica reveals targets for lignocellulose bioengineering
Source: Front Plant Sci. 2015 Nov 4;6:965. doi: 10.3389/fpls.2015.00965 (PMC4631826; doi:10.3389/fpls.2015.00965)

Supplementary Figure S2

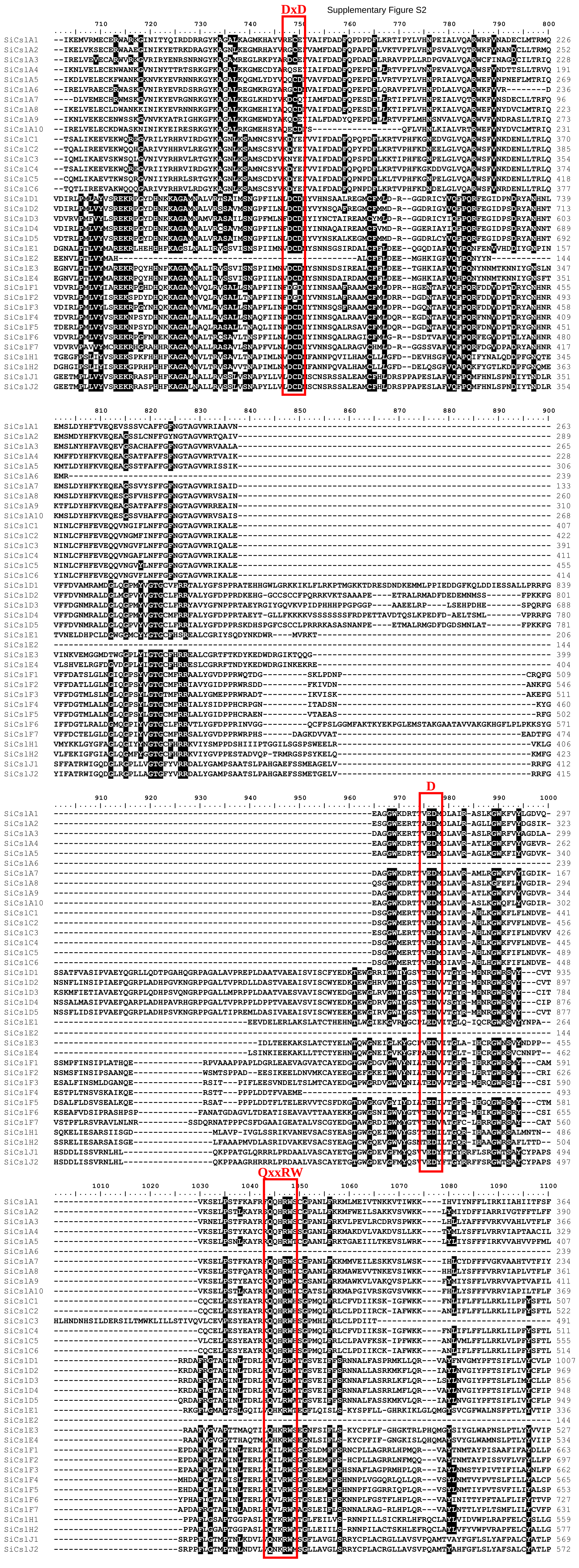

Supplement: Supplementary Figure S2 — Multiple sequence alignment of SiCsl proteins. [file Image2.PDF]
