# Supplementary figures and images for: Integrative analysis and expression profiling of secondary cell wall genes in C4 biofuel model Setaria italica reveals targets for lignocellulose bioengineering
Source: Front Plant Sci. 2015 Nov 4;6:965. doi: 10.3389/fpls.2015.00965 (PMC4631826; doi:10.3389/fpls.2015.00965)

Supplementary Figure S4

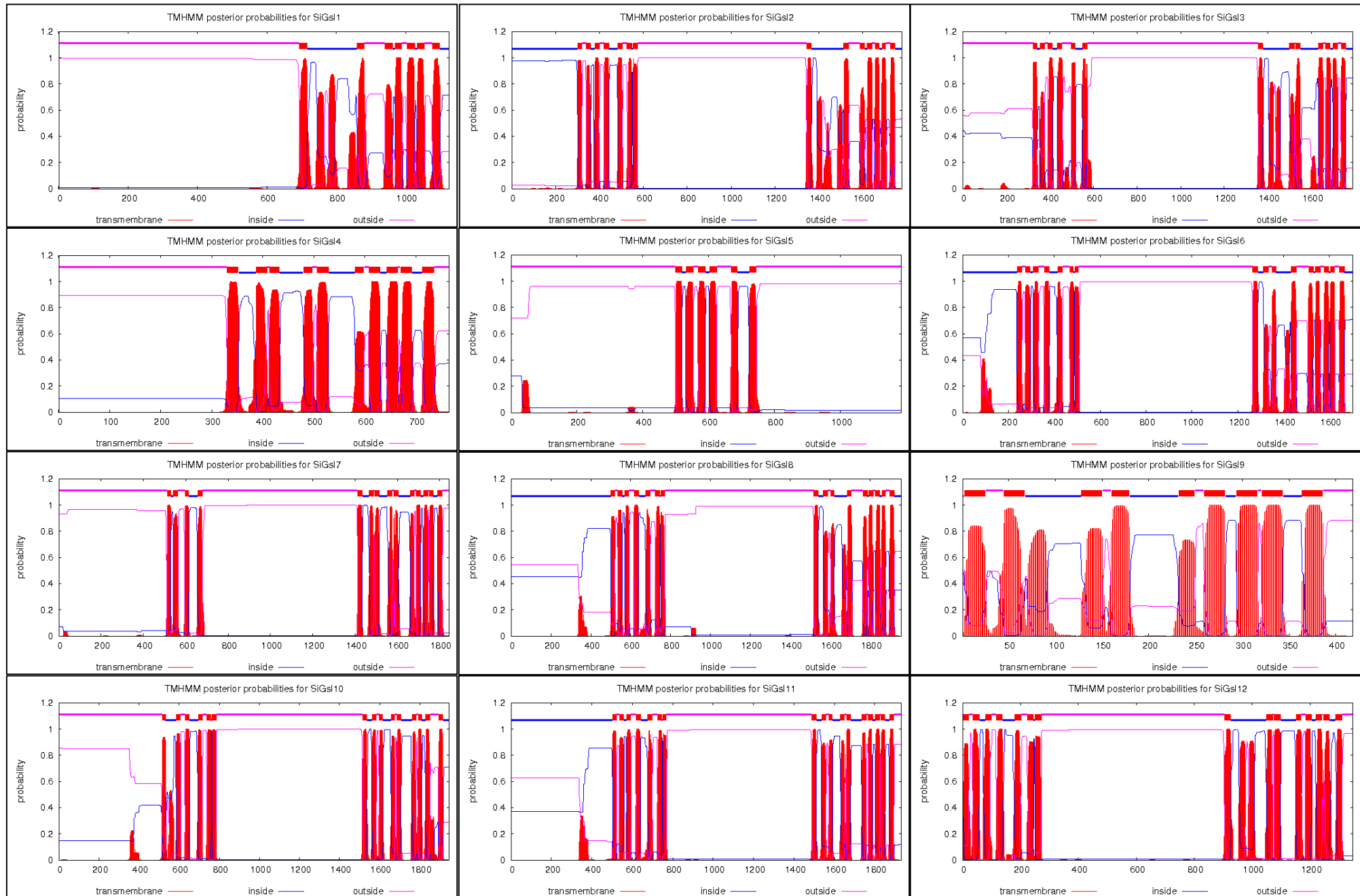

Supplement: Supplementary Figure S4 — Prediction of transmembrane domains in the SiGsl proteins. Red line represents transmembrane, blue line represents inside and pink line represents outside orientation. [file Image4.PDF]

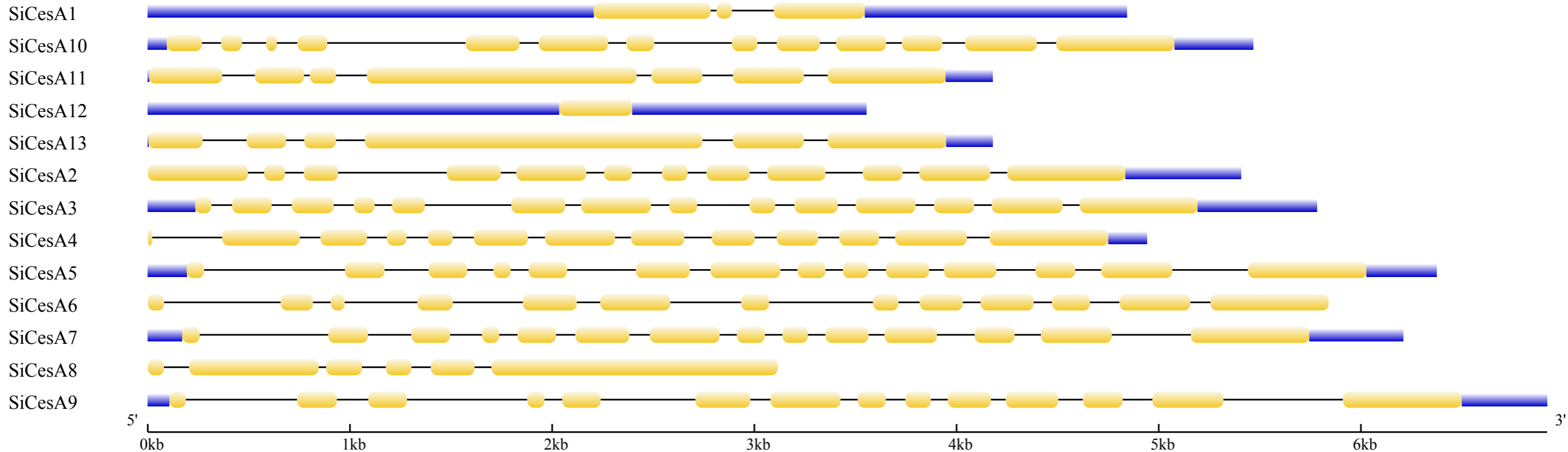

Legend:

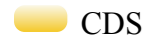

CDS

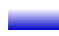

upstream/ downstream

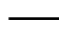

Intron

Supplementary Figure S6

Supplement: Supplementary Figure S6 — Gene structure of SiCesA genes. [file Image6.PDF]
