# Supplementary material for: Integrative analysis and expression profiling of secondary cell wall genes in C4 biofuel model Setaria italica reveals targets for lignocellulose bioengineering
Source: Front Plant Sci. 2015 Nov 4;6:965. doi: 10.3389/fpls.2015.00965 (PMC4631826; doi:10.3389/fpls.2015.00965)
Supplement: Supplementary Figure S5 — Multiple sequence alignment of monolignol biosynthesis proteins. [file Image5.PDF]

Supplementary Figure S5

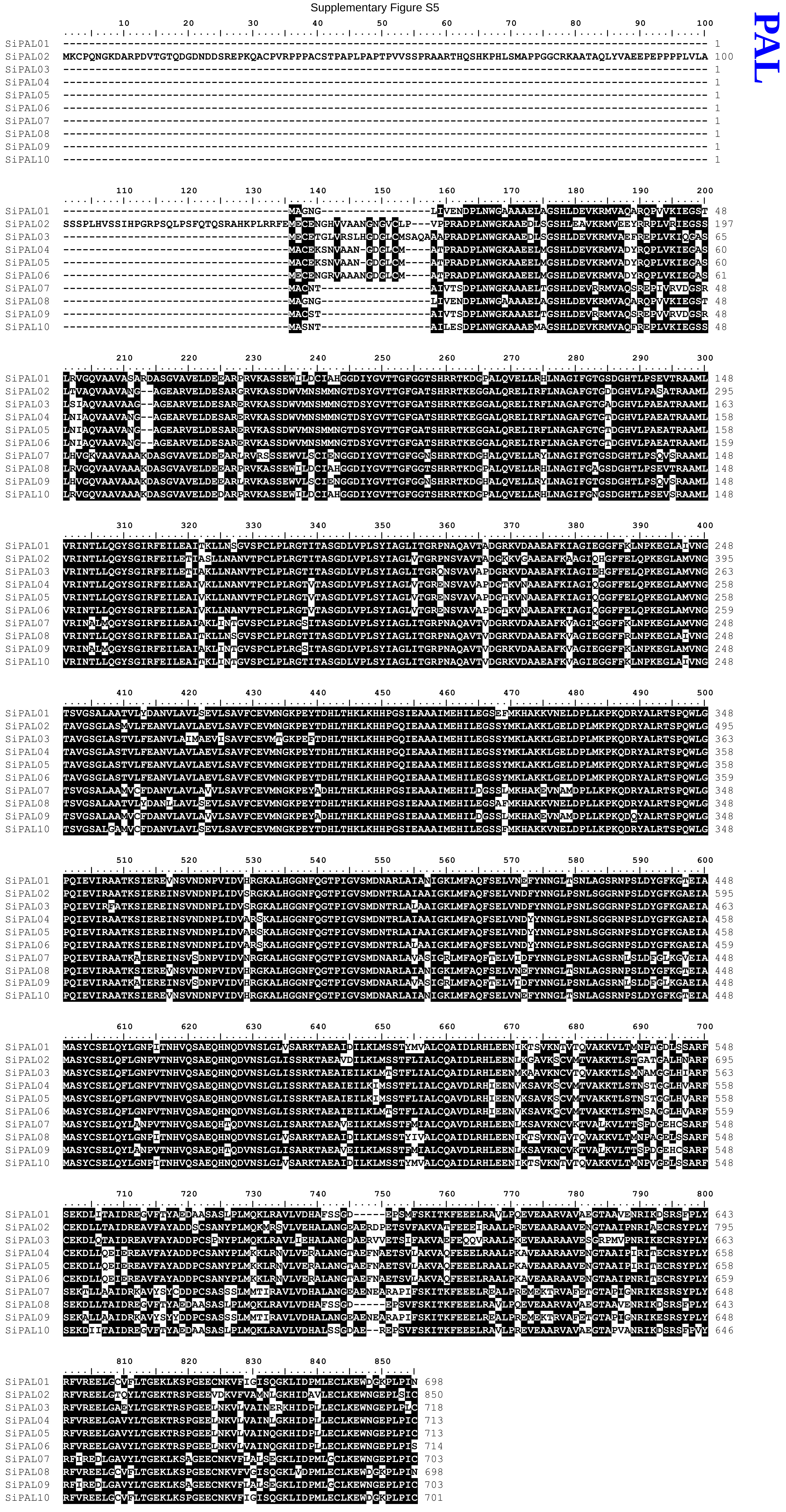

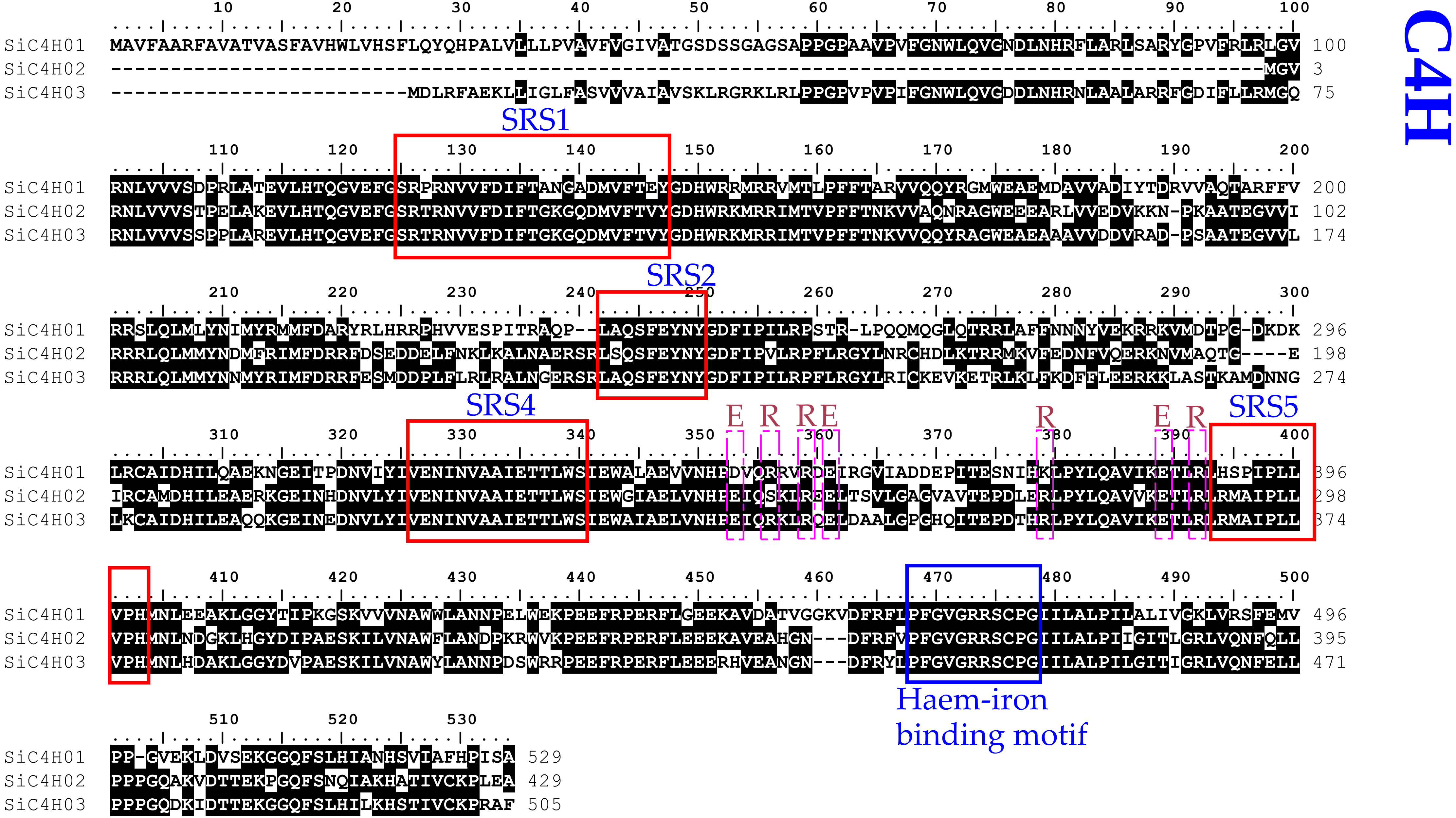
C4H
SRS1
SRS2
SRS4
SRS5
E R RE R E R
Haem-iron binding motif
SiC4H01
SiC4H02
SiC4H03

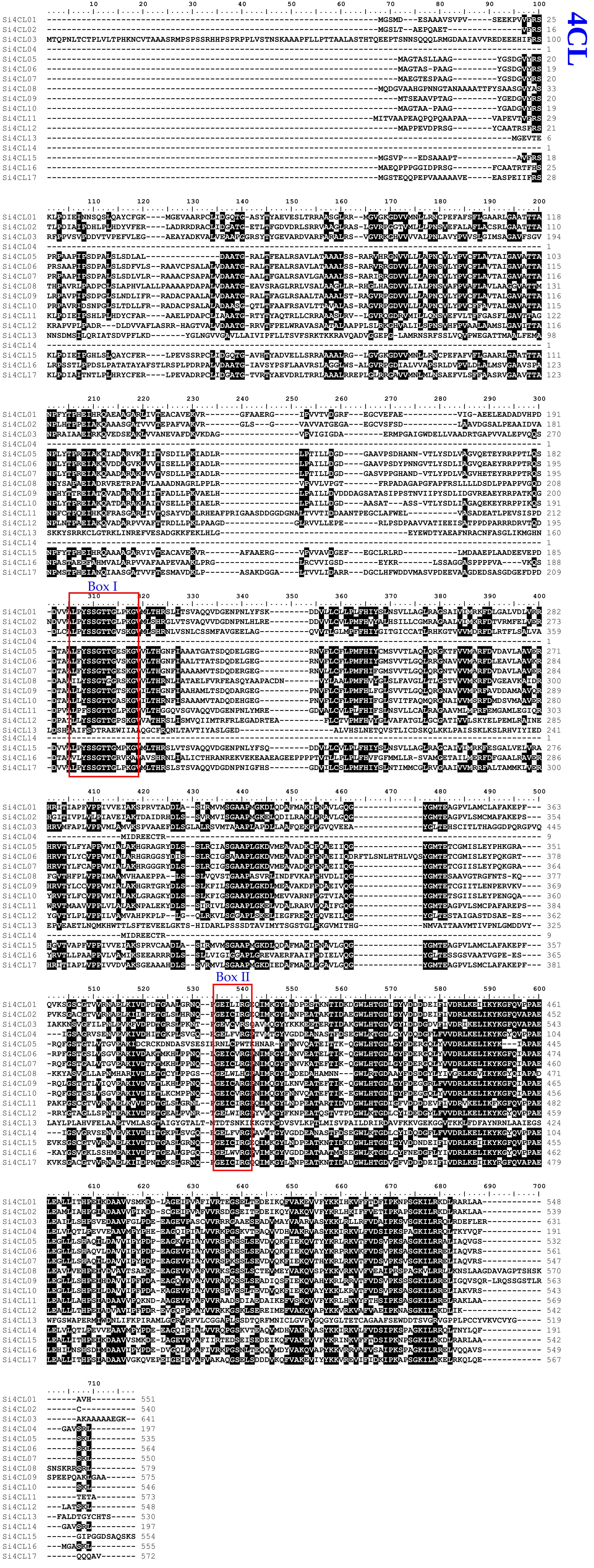
4CL
Box I
Box II

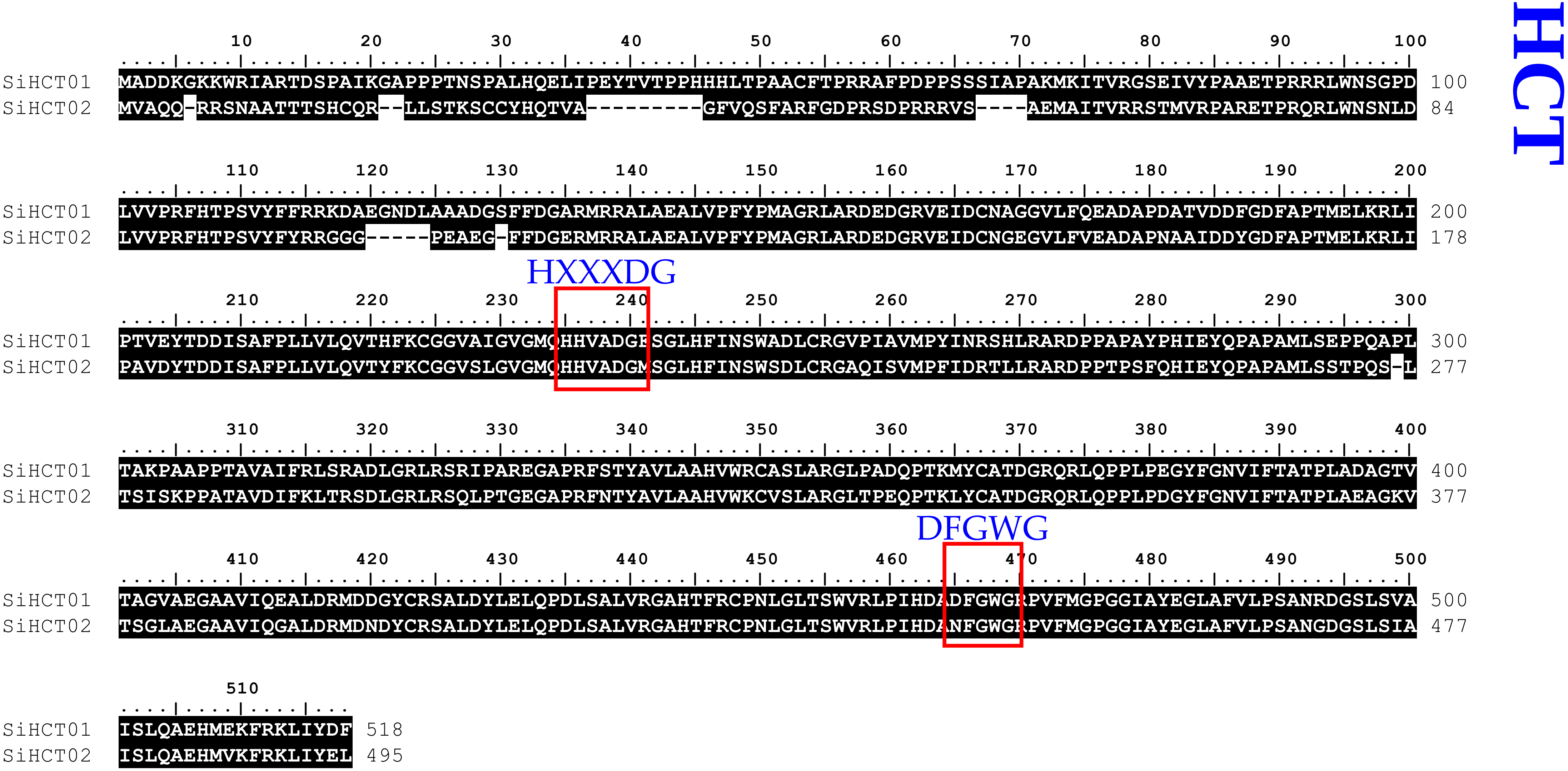
HCT
HXXXDG
DFGWG
SiHCT01
SiHCT02

C3H

```
                   10        20        30        40        50        60        70        80        90       100
           ....|....|....|....|....|....|....|....|....|....|....|....|....|....|....|....|....|....|....|....|
SiC3H01    MDASLLLSVGLAAVLIPLSLALLNRLRVG-------RLPPGPRPWPVLGNLRQIKPIRCRCFQEWAERYGPIISVWFGSGLTVVVSTSELAKEVLKEKDQ 93
SiC3H02    --MELPPAVPFVAVVLATVLFLVTILRRWNHPKLKHNLPPGPRPWPVIGNLNLIGALPHRSIHELSKQYGPLMSLRLGSFPVVVGSSVDAARFILKTHDL 98

                  110       120       130       140       150       160       170       180       190       200
           ....|....|....|....|....|....|....|....|....|....|....|....|....|....|....|....|....|....|....|
SiC3H01    QLADRPRNRSTQRFSRNGQDLIWADYGPHYIKVRKLCNLELFTPKRLEALRPIREDEVTAMVESVH---RDATAPGNEGKPVVVRNHLSMVAFNNITRLA 190
SiC3H02    AFIDRPRMASGRYTGCNYSDVLWAPYGAYWRQARRLWKTEILSARRLRSHEHVRDEEVRAMLRDLYGHGPEAPASAAAGRAVVLLDHLFMANLNAISRMV 198

                  210       220       230       240       250       260       270       280       290       300
           ....|....|....|....|....|....|....|....|....|....|....|....|....|....|....|....|....|....|....|
SiC3H01    FGKRFMNANG----EVDEQGREFKTIVHNGIKIGASLSVAEFIWYLRWLCPLNEELYKTHNERRDRLTMKIIEEHAKALKESG---AKQHFVDALFTLKE 283
SiC3H02    LGKKYVVHGGTGSSAAATTPEEFKWMIDEFFFLSGALNVGDMIPWLGWLDPQIKRIKRLG-RKFDRFLEQVLDEHNERRRREGEEFAAMDMVDLLLELAD 297

                  310       320       330       340       350       360       370       380       390       400
           ....|....|....|....|....|....|....|....|....|....|....|....|....|....|....|....|....|....|....|
SiC3H01    QYDL--------------------------------------------------------------------------SEDTVIG--------------- 294
SiC3H02    DPNLGAEVDLEHVLVRGAELGERLQQVGTNSRRGRRACSAEDGCVLLRCRHGGTCACAAGTRKKDMLLLACFMGTGDARADDMLGPAALDHAARQPAYGG 397

                  410       420       430       440       450       460       470       480       490       500
           ....|....|....|....|....|....|....|....|....|....|....|....|....|....|....|....|....|....|....|
SiC3H01    -LLWDMITAGMDTTVISVEWAMAELVRNPRVQKKLQEELDRVVGRDRVMLETDFQSLPYLQAVVKESLRLHPPTPLMLPHKASTSVKIGGYDIPKGTNVM 393
SiC3H02    NVAEDLIVGGTDTSSVTIEWAMSELLRNPDALAKATEELDRVIGRERLVTEGDIPNLPYMEAVVKEAMRLHPVTPLLAPRLSREDASMGGYDVPAGTLVF 497
```

[FW]-[SGNH]-x-[GD]-{F}-[RKHPT]-{P}-C-[LIVMFAP]-[GAD]

```
                  510       520       530       540       550       560       570       580       590       600
           ....|....|....|....|....|....|....|....|....|....|....|....|....|....|....|....|....|....|....|
SiC3H01    VNVWAVARDPKVWSN-PLEYRPERFMEESIDIKGSDFRVLPFGAGRRVCPGAQLGINLVASMIGHLLHHFEWSLPEGTRPEDVDMMESPGLVTFMGTPLQ 492
SiC3H02    VNVWAIGRDPAVWGDAPSEFRPERFVGSSLDVKGQDFELLPFGSGRRMCPGIGLGLKMVQLILANLVHGFAWRLPDGMVKEELSMEEKFGLSMPRMVPLE 597

                  610       620
           ....|....|....|....|
SiC3H01    AVARPRLENEELYKRVPVEM 512
SiC3H02    AVPEPRLP-AHLYAGP---- 612
```

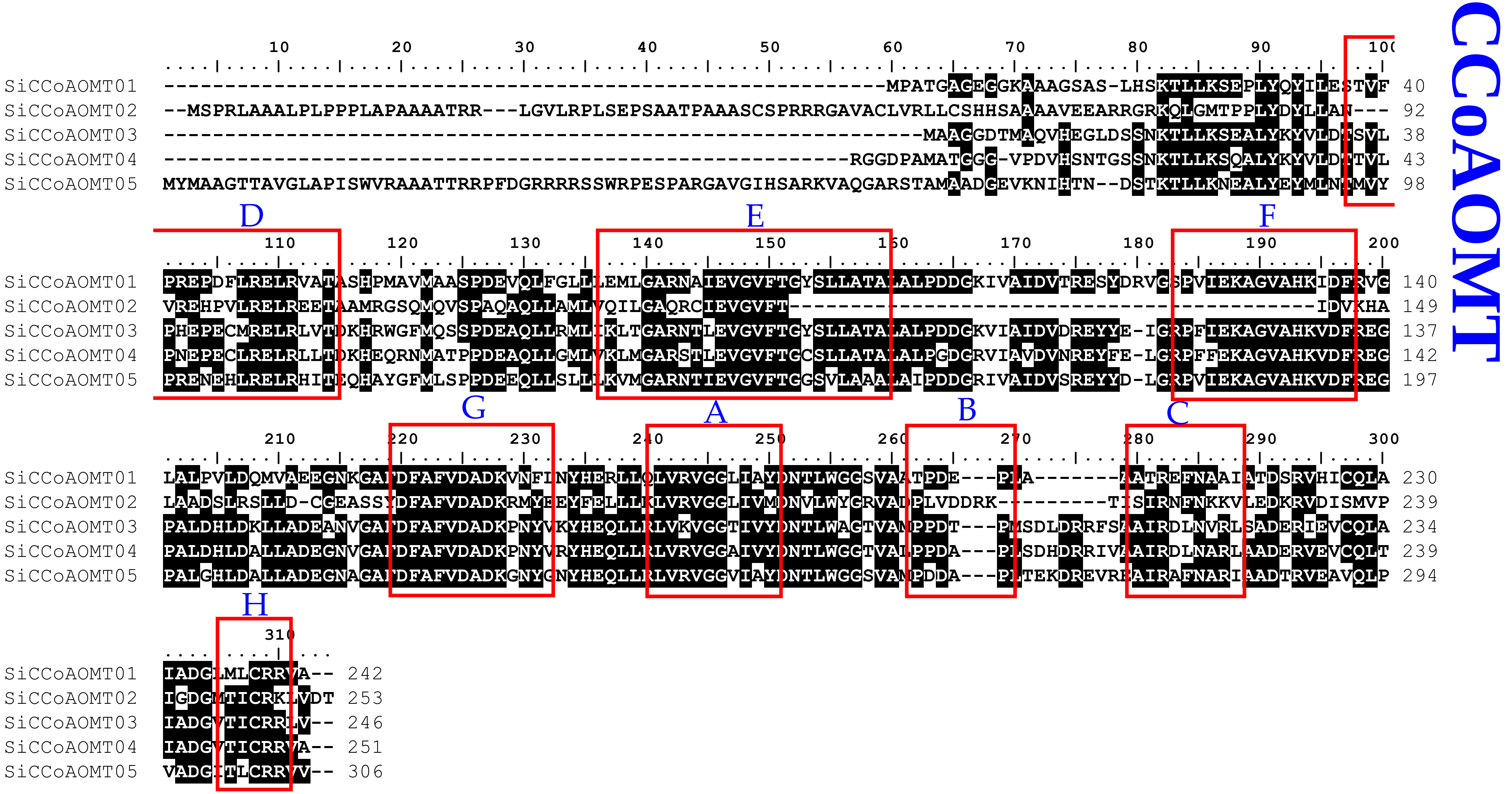
CCoAOMT
D
E
F
G
A
B
C
H
SiCCoAOMT01
SiCCoAOMT02
SiCCoAOMT03
SiCCoAOMT04
SiCCoAOMT05
40
92
38
43
98
140
149
137
142
197
230
239
234
239
294
242
253
246
251
306

COMT

```
                   10        20        30        40        50        60        70        80        90       100
          ....|....|....|....|....|....|....|....|....|....|....|....|....|....|....|....|....|....|....|....|
SiCOMT01  MMELANMISVPMALTAVIRLGVPSAVWAGGANAPLSAAELLPEGHPDPSVLERLLRLLASRGVFSEHGGGSAGSPRRFALTAVGRTLVPSGPSGASYADY 100
SiCOMT03  -------------MRVVQSLG-----WSGGRLCLRHPRQRANNIQPASTACGCLVVLCTVKELLLKVQLQFRNCGKRVVHVAG--EMFGSVPSGDAVLMK 80

                  110       120       130       140       150       160       170       180       190       200
          ....|....|....|....|....|....|....|....|....|....|....|....|....|....|....|....|....|....|....|....|
SiCOMT01  VLQHHQDALVSAWPRLHEAVLDPAGPEPFARANAGVPAYAYYGRDREANEVMLRAMTGVSEPFMEALLDGYAAGFEGVATLVDVGGSSGACLEMIMRRVD 200
SiCOMT03  VIVIQSVLLETPES-------TPASLDSFT-IDMIILVNFKGGKERTEQEYAKLGRD-----------AGFTGGFQSTYIFCNIY-----ALEFTK---- 152

                  210       220       230       240
          ....|....|....|....|....|....|....|....|..
SiCOMT01  TIREGINFDLPDVVAAAPPIVGTEYSVACSSITLSAFRSHPITLVAW 247
SiCOMT03  ----------------------------------------------- 152
```

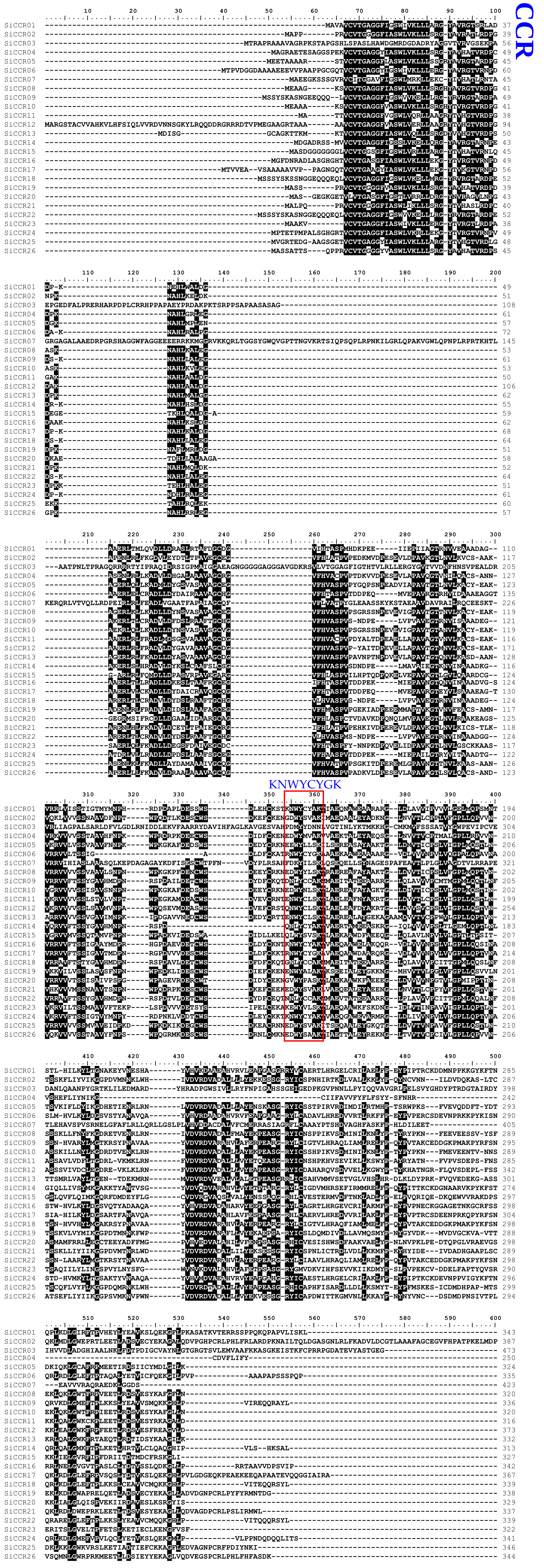
CCR
KNWYCYGK

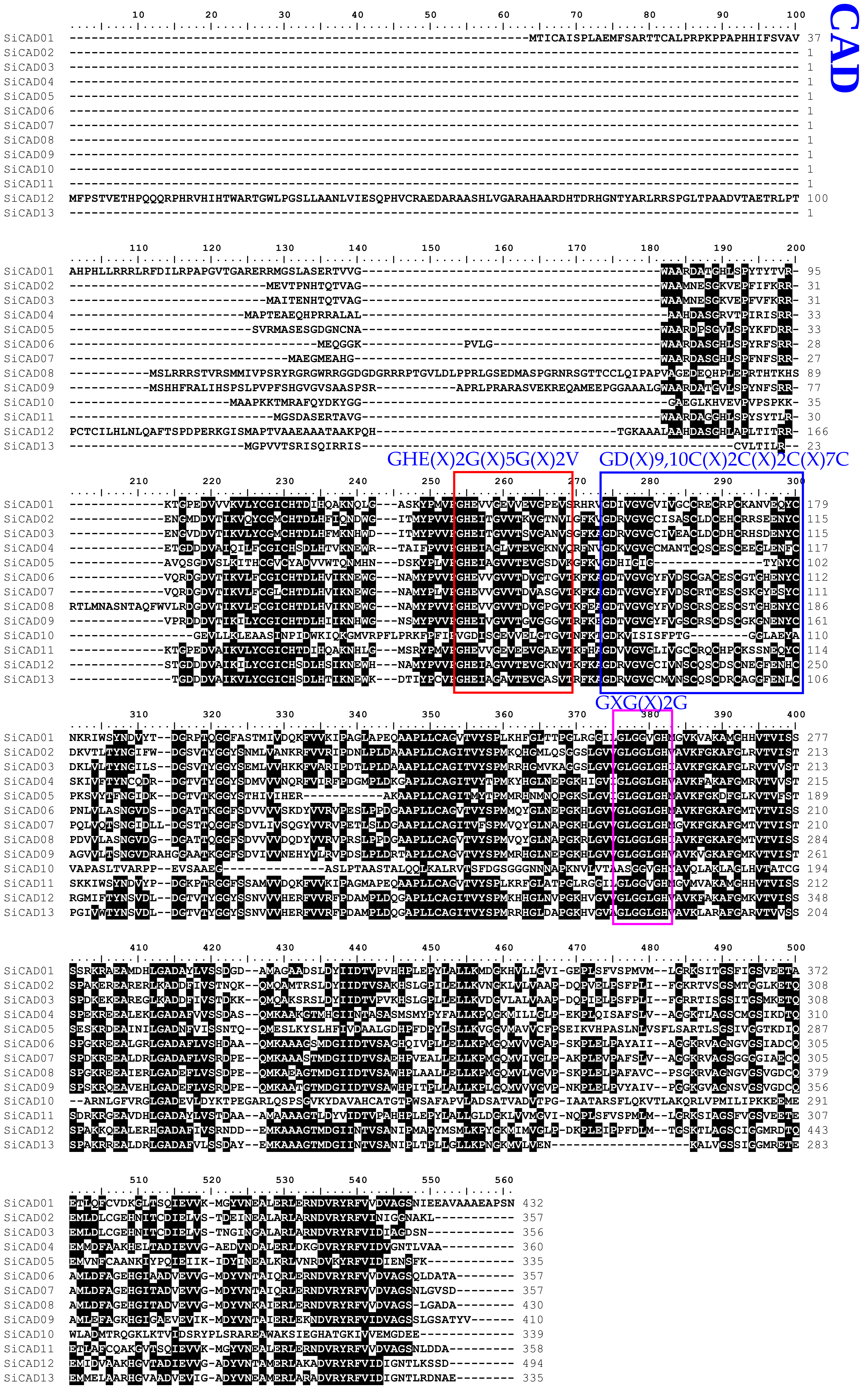
CAD
GHE(X)2G(X)5G(X)2V
GD(X)9,10C(X)2C(X)2C(X)7C
GXG(X)2G
SiCAD01
SiCAD02
SiCAD03
SiCAD04
SiCAD05
SiCAD06
SiCAD07
SiCAD08
SiCAD09
SiCAD10
SiCAD11
SiCAD12
SiCAD13
