# Supplementary material for: Integrative analysis and expression profiling of secondary cell wall genes in C4 biofuel model Setaria italica reveals targets for lignocellulose bioengineering
Source: Front Plant Sci. 2015 Nov 4;6:965. doi: 10.3389/fpls.2015.00965 (PMC4631826; doi:10.3389/fpls.2015.00965)
Supplement: Supplementary Figure S7 — Gene structure of SiCsl genes. [file Image7.PDF]

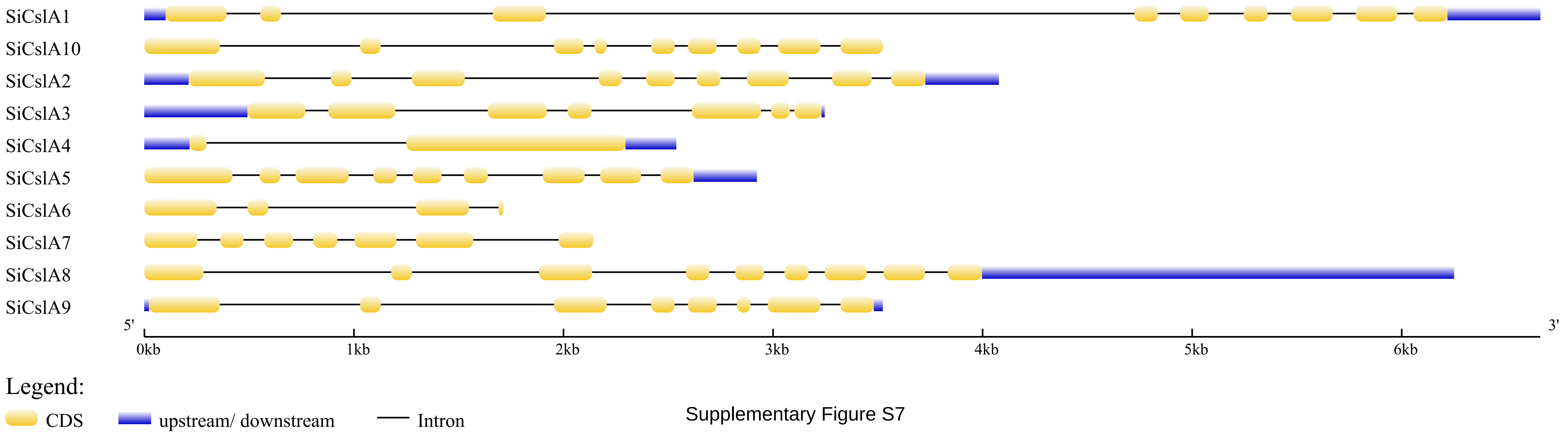

Supplementary Figure S7

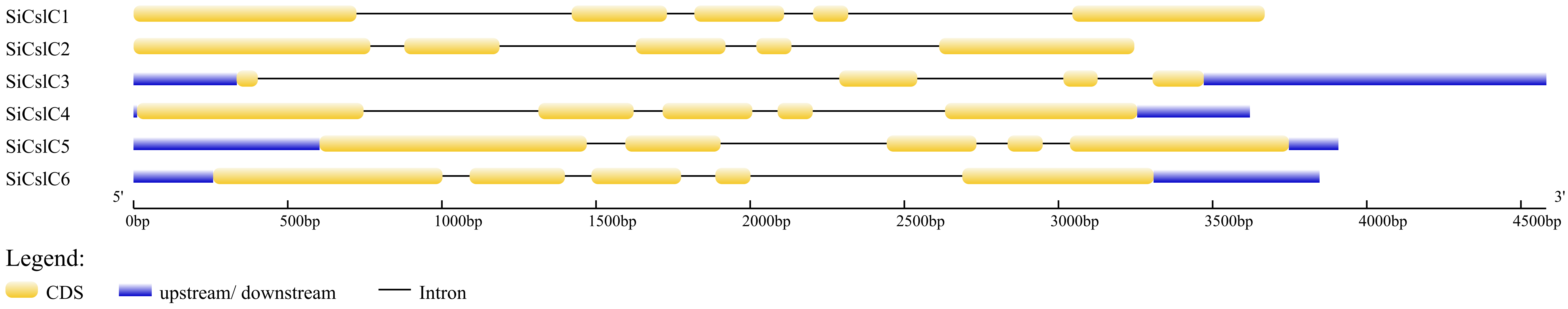
SiCslC1
SiCslC2
SiCslC3
SiCslC4
SiCslC5
SiCslC6
5'
3'
0bp
500bp
1000bp
1500bp
2000bp
2500bp
3000bp
3500bp
4000bp
4500bp
Legend:
CDS
upstream/ downstream
Intron

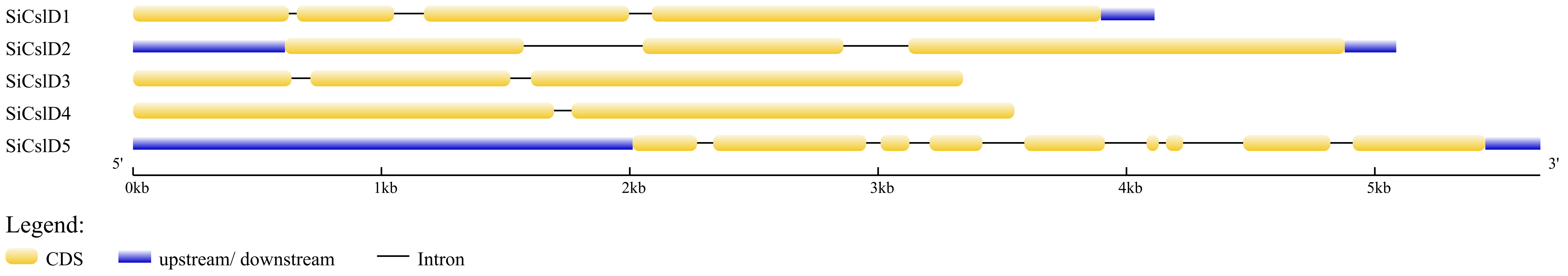
SiCslD1
SiCslD2
SiCslD3
SiCslD4
SiCslD5
5'
3'
0kb
1kb
2kb
3kb
4kb
5kb
Legend:
CDS
upstream/ downstream
Intron

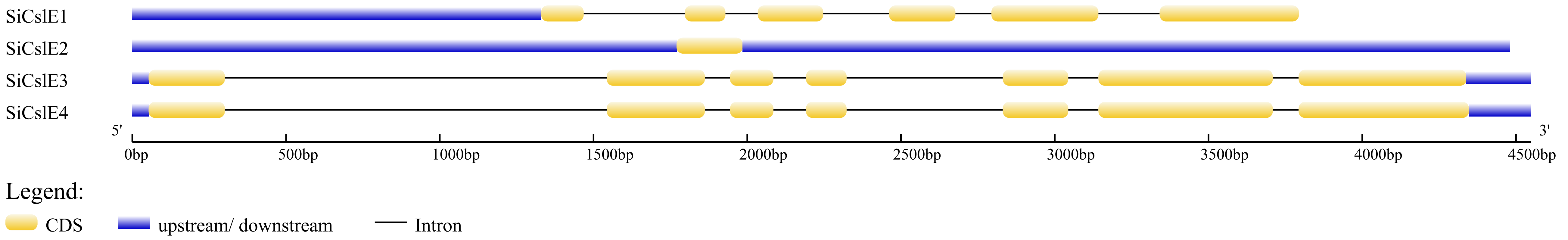
SiCslE1
SiCslE2
SiCslE3
SiCslE4
5'
3'
0bp
500bp
1000bp
1500bp
2000bp
2500bp
3000bp
3500bp
4000bp
4500bp
Legend:
CDS
upstream/ downstream
Intron

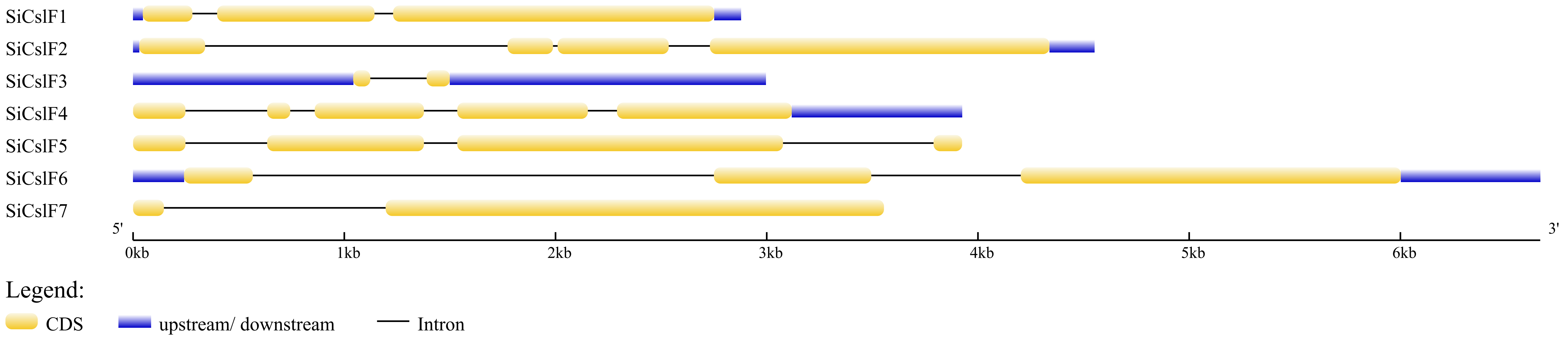
SiCslF1
SiCslF2
SiCslF3
SiCslF4
SiCslF5
SiCslF6
SiCslF7
5'
3'
0kb
1kb
2kb
3kb
4kb
5kb
6kb
Legend:
CDS
upstream/ downstream
Intron

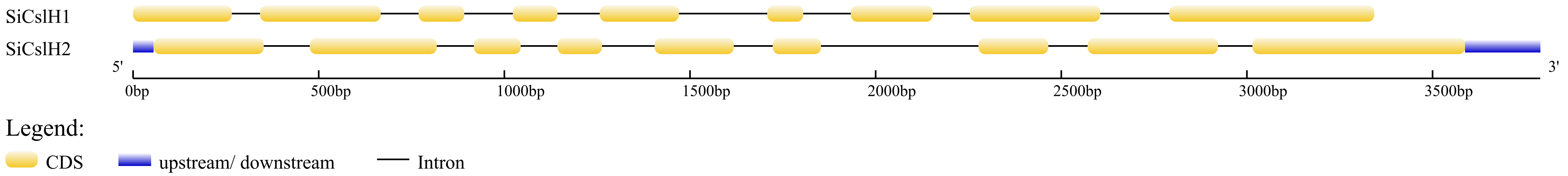
SiCslH1
SiCslH2
5'
3'
0bp
500bp
1000bp
1500bp
2000bp
2500bp
3000bp
3500bp
Legend:
CDS
upstream/ downstream
Intron

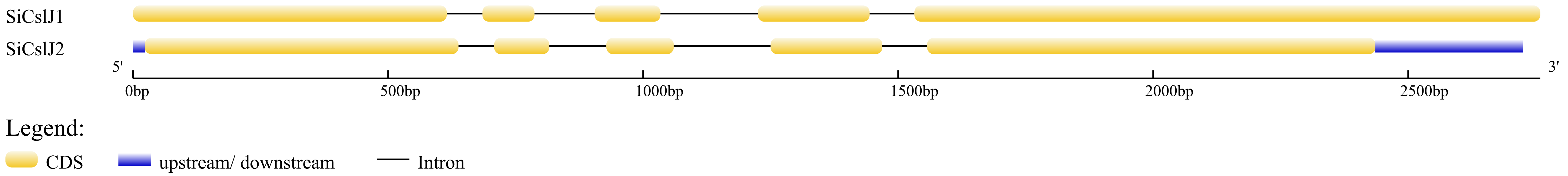
SiCslJ1
SiCslJ2
5'
3'
0bp
500bp
1000bp
1500bp
2000bp
2500bp
Legend:
CDS
upstream/ downstream
Intron
