# Supplementary material for: Integrative analysis and expression profiling of secondary cell wall genes in C4 biofuel model Setaria italica reveals targets for lignocellulose bioengineering
Source: Front Plant Sci. 2015 Nov 4;6:965. doi: 10.3389/fpls.2015.00965 (PMC4631826; doi:10.3389/fpls.2015.00965)
Supplement: Supplementary Figure S8 — Gene structure of SiGsl genes. [file Image8.PDF]

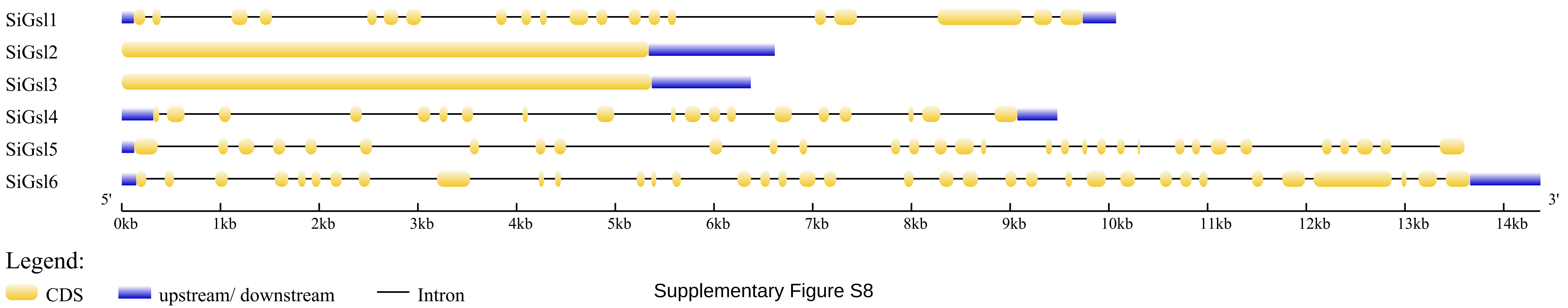

Supplementary Figure S8

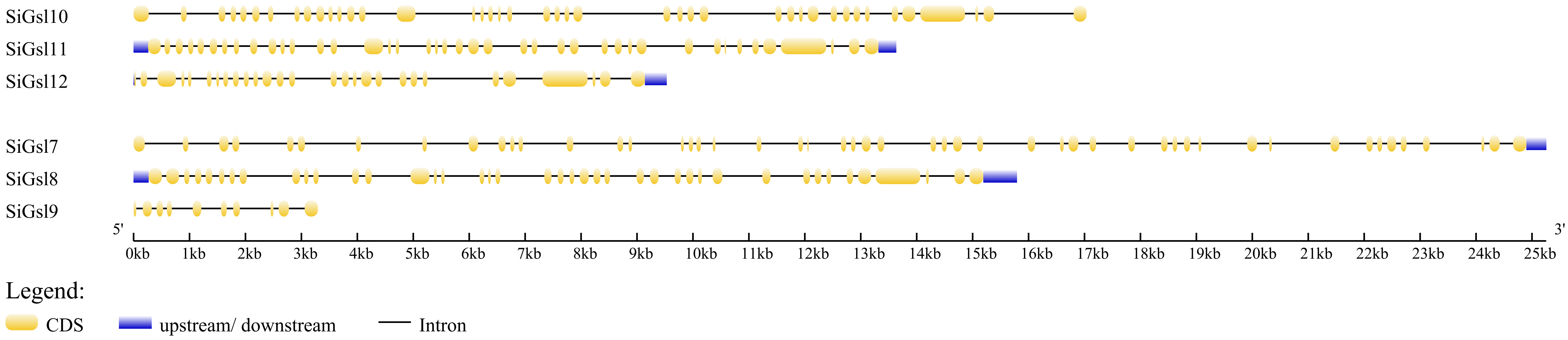
SiGsl10
SiGsl11
SiGsl12
SiGsl7
SiGsl8
SiGsl9
5'
3'
0kb
1kb
2kb
3kb
4kb
5kb
6kb
7kb
8kb
9kb
10kb
11kb
12kb
13kb
14kb
15kb
16kb
17kb
18kb
19kb
20kb
21kb
22kb
23kb
24kb
25kb
Legend:
CDS
upstream/ downstream
Intron
