# Supplementary material for: Integrative analysis and expression profiling of secondary cell wall genes in C4 biofuel model Setaria italica reveals targets for lignocellulose bioengineering
Source: Front Plant Sci. 2015 Nov 4;6:965. doi: 10.3389/fpls.2015.00965 (PMC4631826; doi:10.3389/fpls.2015.00965)
Supplement: Supplementary Figure S9 — Gene structure of monolignol biosynthesis genes. [file Image9.PDF]

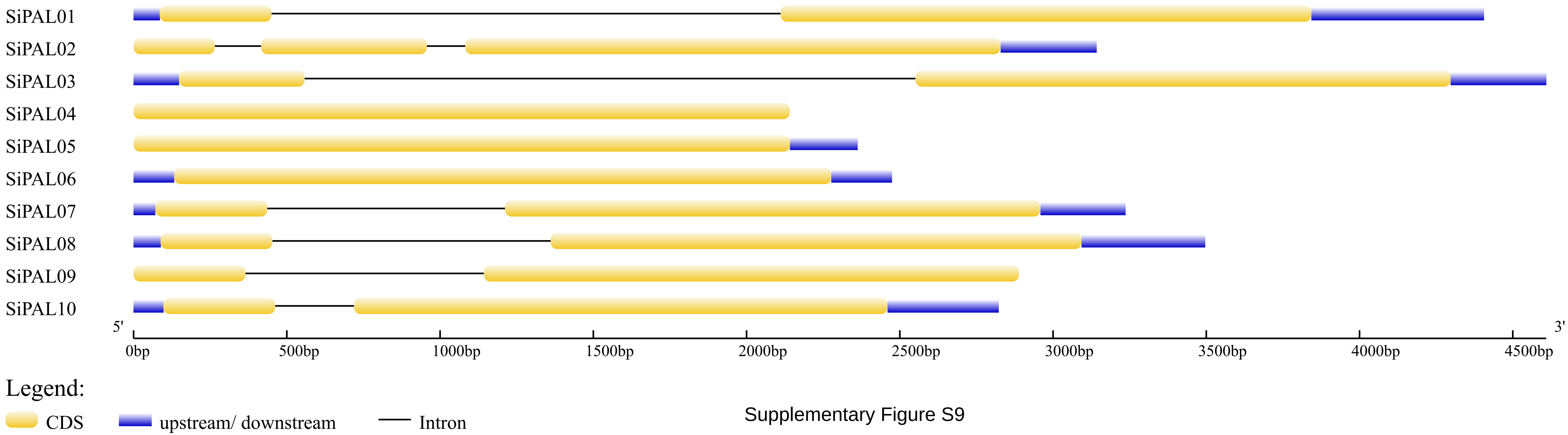

Supplementary Figure S9

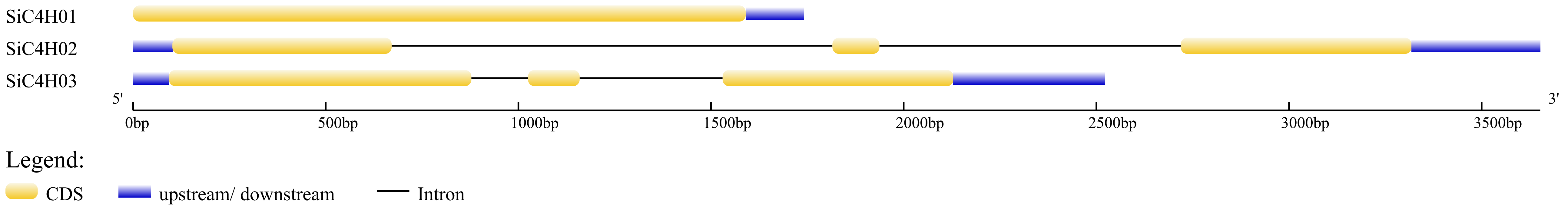
SiC4H01
SiC4H02
SiC4H03
5'
3'
0bp
500bp
1000bp
1500bp
2000bp
2500bp
3000bp
3500bp
Legend:
CDS
upstream/ downstream
Intron

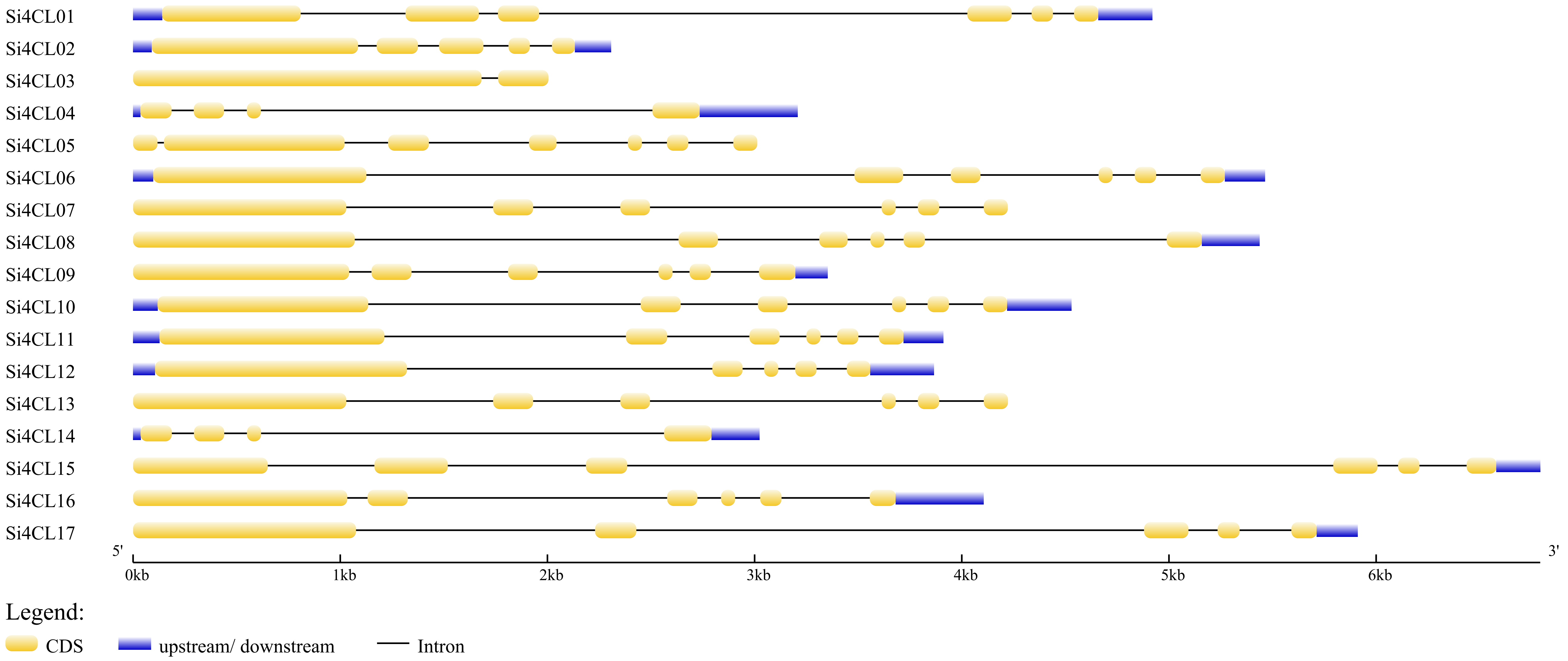
Si4CL01
Si4CL02
Si4CL03
Si4CL04
Si4CL05
Si4CL06
Si4CL07
Si4CL08
Si4CL09
Si4CL10
Si4CL11
Si4CL12
Si4CL13
Si4CL14
Si4CL15
Si4CL16
Si4CL17
5'
3'
0kb
1kb
2kb
3kb
4kb
5kb
6kb
Legend:
CDS
upstream/ downstream
Intron

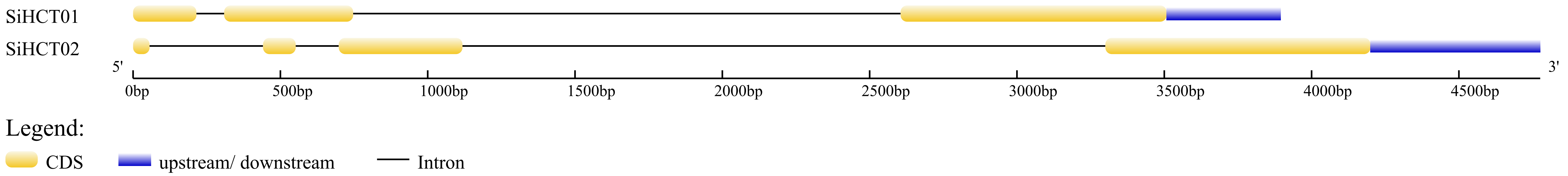
SiHCT01
SiHCT02
5'
3'
0bp
500bp
1000bp
1500bp
2000bp
2500bp
3000bp
3500bp
4000bp
4500bp
Legend:
CDS
upstream/ downstream
Intron

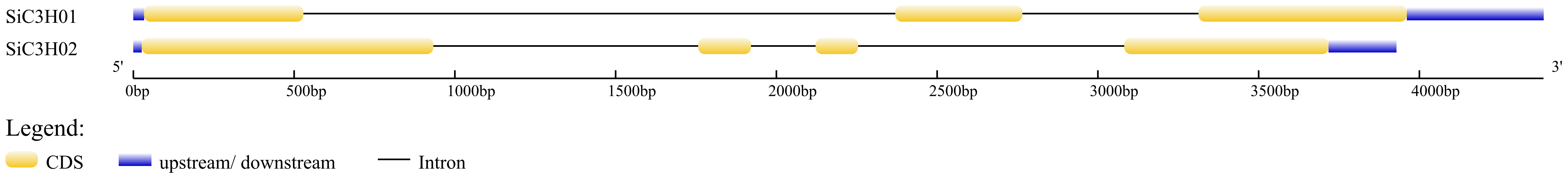
SiC3H01
SiC3H02
5'
3'
0bp
500bp
1000bp
1500bp
2000bp
2500bp
3000bp
3500bp
4000bp
Legend:
CDS
upstream/ downstream
Intron

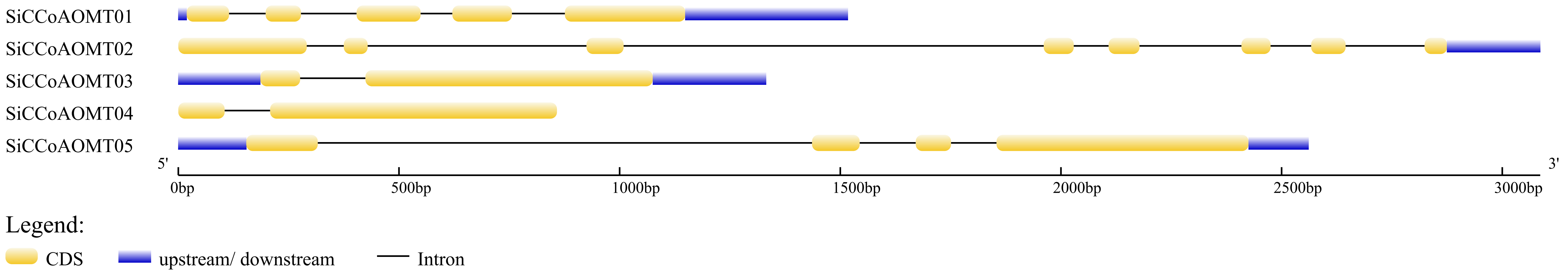

SiCCoAOMT01
SiCCoAOMT02
SiCCoAOMT03
SiCCoAOMT04
SiCCoAOMT05
5'
3'
0bp
500bp
1000bp
1500bp
2000bp
2500bp
3000bp
Legend:
CDS
upstream/ downstream
Intron

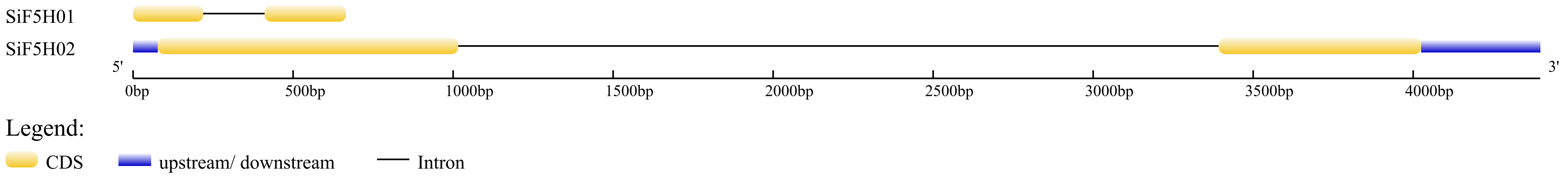
SiF5H01
SiF5H02
5'
3'
0bp
500bp
1000bp
1500bp
2000bp
2500bp
3000bp
3500bp
4000bp
Legend:
CDS
upstream/ downstream
Intron

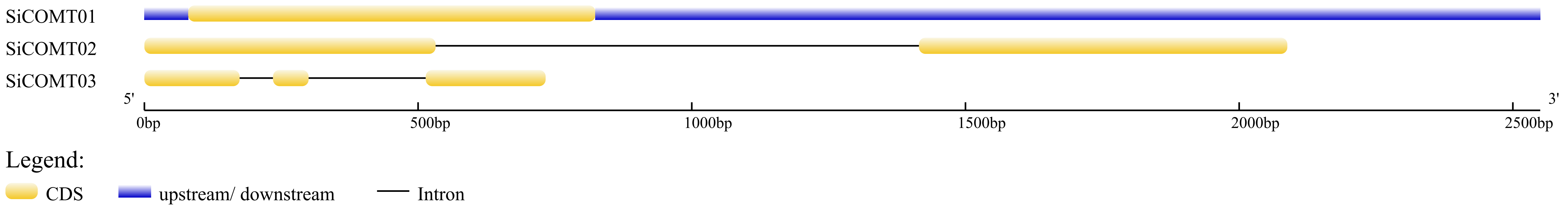
SiCOMT01
SiCOMT02
SiCOMT03
5'
3'
0bp
500bp
1000bp
1500bp
2000bp
2500bp
Legend:
CDS
upstream/ downstream
Intron

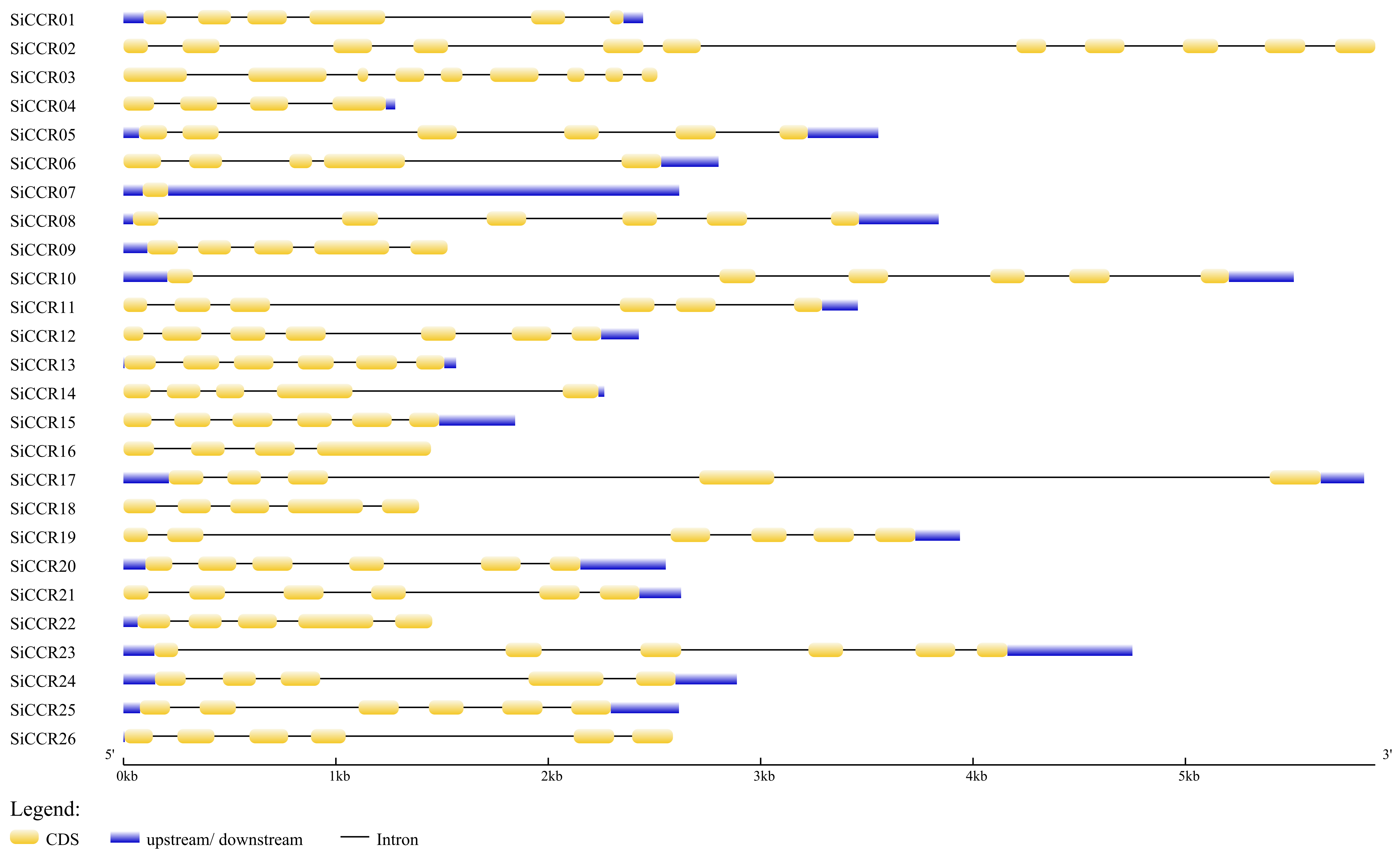
SiCCR01
SiCCR02
SiCCR03
SiCCR04
SiCCR05
SiCCR06
SiCCR07
SiCCR08
SiCCR09
SiCCR10
SiCCR11
SiCCR12
SiCCR13
SiCCR14
SiCCR15
SiCCR16
SiCCR17
SiCCR18
SiCCR19
SiCCR20
SiCCR21
SiCCR22
SiCCR23
SiCCR24
SiCCR25
SiCCR26
5'
3'
0kb
1kb
2kb
3kb
4kb
5kb
Legend:
CDS
upstream/ downstream
Intron

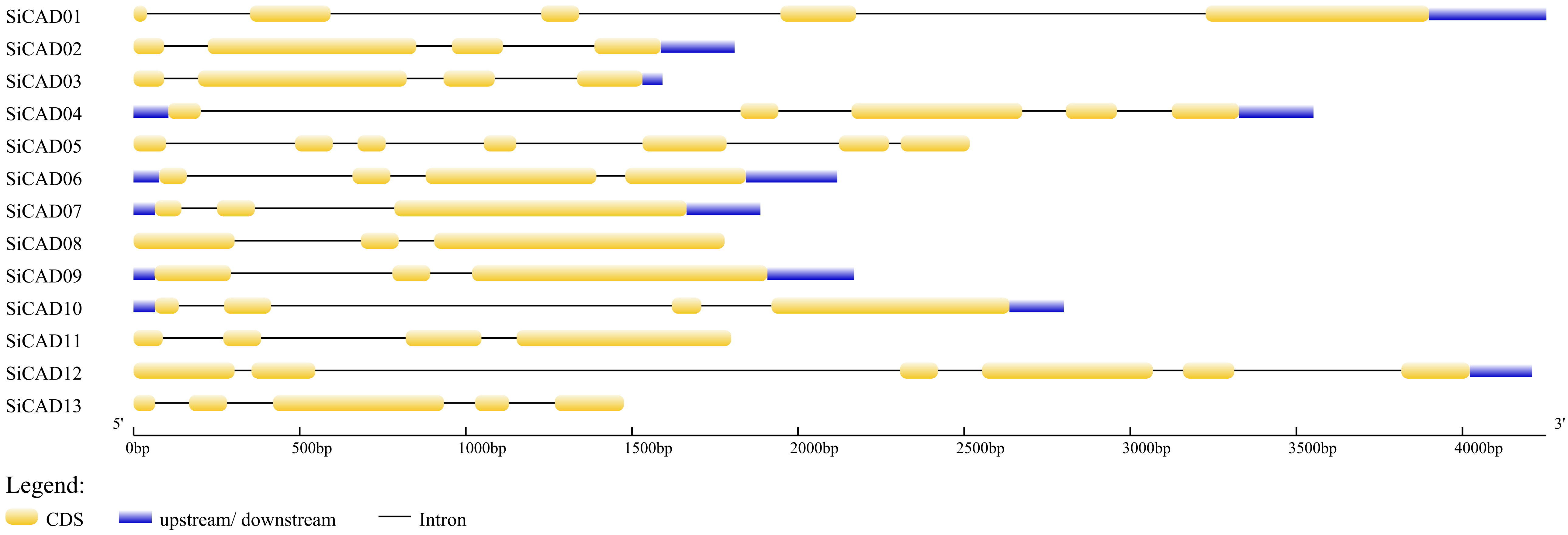
SiCAD01
SiCAD02
SiCAD03
SiCAD04
SiCAD05
SiCAD06
SiCAD07
SiCAD08
SiCAD09
SiCAD10
SiCAD11
SiCAD12
SiCAD13
5'
3'
0bp
500bp
1000bp
1500bp
2000bp
2500bp
3000bp
3500bp
4000bp
Legend:
CDS
upstream/ downstream
Intron
