# Supplementary material for: Integrative analysis and expression profiling of secondary cell wall genes in C4 biofuel model Setaria italica reveals targets for lignocellulose bioengineering
Source: Front Plant Sci. 2015 Nov 4;6:965. doi: 10.3389/fpls.2015.00965 (PMC4631826; doi:10.3389/fpls.2015.00965)
Supplement: Supplementary Table S1 — Details of primers used in qRT-PCR analysis. [file Table1.DOC]

**Supplementary Table S1.** Details of primers used in qRT-PCR analysis.

| **Primer Name** | **Forward Sequence (5'-3')** | **Reverse Sequence (5'-3')** |
| --- | --- | --- |
| SiPAL2 | AAGTTCATGTGAATGTTGTTGTGTTC | GCCCAGGCAATGCACATC |
| SiHCT1 | ACACGAGCCATCCATCAGTA | CAAAGAATCTTGCTGCAACC |
| SiGsl2 | AGGTAGCATTGTATGGTTGGC | CCCGTCTCTCTCATTCACTC |
| SiCesA22 | CAGCAGAGGGTTGGAGATTT | CGTCGAGACTACCACCATCA |
| SiGsl13 | CGACATGTTCAAGTCGGT | GCGTCGTAGCAGTTCTTGAG |
| SiCCR22 | GAACAGGAAGCAATGCAAGCA | ACAAGATCAACGAGCACGACAT |
| SiGsl12 | GCACGGGCCTATGAGATAAT | GTCCTCTGCTGAATGCTTGA |
| SiF5H2 | GGAGGAGTCTGGTGGCTTT | TTCCTTATACACTAGGCATGCAG |
| SiCesA5 | GGGTGAGCCGAAGAGAAAG | CGCCTGCCTTATTCAGAAC |
| SiCCR7 | GTATGGGCATCAGTATGGCA | GGGCTTTATTGGCGTTTCT |
| SiCCoAOMT3 | GTCTGGTCGTCTCGGTGCTT | TGTATGCGGTTGCTCCAATTC |
| SiCAD1 | GTGGACCGTGAGGCTGTGT | TCAGCCCAAGGAAGCAGAAA |
| SiPAL7 | TGCCCATCAACTAAGGAGAA | TACAACCAAAGGTCACCCAA |
| SiC4H2 | TGCCTGTTGAGTGATGGG | TGCAAGAACATAACCAAGTGAA |
| SiCAD6 | TGGGAGACGAAGAGTGATGA | TGCCACACAAGAAATCCAAT |
| SiCOMT2 | TGTTCCACGTCGACATGATCA | ATGCGTTGGCGTAGATGTAGGT |
| Actin2 | CGCATATGTGGCTCTTGACT | GGGCACCTAAATCTCTCTGC |
