# Supplementary material for: Integrative analysis and expression profiling of secondary cell wall genes in C4 biofuel model Setaria italica reveals targets for lignocellulose bioengineering
Source: Front Plant Sci. 2015 Nov 4;6:965. doi: 10.3389/fpls.2015.00965 (PMC4631826; doi:10.3389/fpls.2015.00965)
Supplement: Supplementary Table S3 — Details of various domains present in SiCesA proteins. [file Table3.DOC]

**Supplementary Table S3.** Details of various domains present in SiCesA proteins.

| **Protein** | **Cellulose synthase**  **(PF03552)** | | **Zinc-binding RING-finger**  **(PF14569)** | | **Glycosyl transferase family group 2**  **(PF13632)** | |
| --- | --- | --- | --- | --- | --- | --- |
|  | **Start** | **End** | **Start** | **End** | **Start** | **End** |
| **SiCesA1** | 355 | 1077 | 9 | 86 | 724 | 909 |
| **SiCesA2** | 358 | 1086 | 31 | 106 | 735 | 920 |
| **SiCesA3** | 352 | 1082 | 29 | 106 | 730 | 916 |
| **SiCesA4** | 387 | 1091 | 72 | 149 | 709 | 922 |
| **SiCesA5** | 352 | 1071 | 31 | 108 | 717 | 901 |
| **SiCesA6** | 264 | 981 | 21 | 94 | 647 | 814 |
| **SiCesA7** | 352 | 1071 | 31 | 108 | 717 | 901 |
| **SiCesA8** | 134 | 879 | - | - | - | - |
| **SiCesA9** | 350 | 1069 | 29 | 106 | 715 | 899 |
| **SiCesA10** | 261 | 977 | - | - | 633 | 811 |
| **SiCesA11** | 354 | 1084 | 29 | 106 | 733 | 918 |
| **SiCesA12** | 354 | 1074 | 10 | 87 | 722 | 907 |
| **SiCesA13** | 309 | 1070 | 27 | 103 | 739 | 902 |

**-** Not present
