# Supplementary material for: Integrative analysis and expression profiling of secondary cell wall genes in C4 biofuel model Setaria italica reveals targets for lignocellulose bioengineering
Source: Front Plant Sci. 2015 Nov 4;6:965. doi: 10.3389/fpls.2015.00965 (PMC4631826; doi:10.3389/fpls.2015.00965)
Supplement: Supplementary Table S4 — Details of various domains present in SiCsl proteins. [file Table4.DOC]

**Supplementary Table S4.** Details of various domains present in SiCsl proteins.

| **Protein** | **Cellulose**  **synthase (PF03552)** | | **Glycosyltransferase**  **like family 2**  **(PF13641)** | | **Glycosyl transferase**  **family group 2**  **(PF13632)** | | **Glycosyl transferase**  **family 2**  **(PF00535)** | | **Glycosyl transferase**  **family 21**  **(PF13506)** | | **RING/Ubox like**  **zinc-binding domain**  **(PF14570)** | |
| --- | --- | --- | --- | --- | --- | --- | --- | --- | --- | --- | --- | --- |
|  | **Start** | **End** | **Start** | **End** | **Start** | **End** | **Start** | **End** | **Start** | **End** | **Start** | **End** |
| **SiCslA1** | - | - | 84 | 319 | 177 | 414 | 87 | 253 | 144 | 318 | - | - |
| **SiCslA2** | - | - | 110 | 345 | 203 | 448 | 113 | 280 | 170 | 344 | - | - |
| **SiCslA3** | - | - | 86 | 321 | 179 | 415 | 89 | 261 | 145 | 320 | - | - |
| **SiCslA4** | - | - | 49 | 284 | 142 | 363 | 52 | 225 | 112 | 283 | - | - |
| **SiCslA5** | - | - | 127 | 362 | 220 | 441 | 131 | 304 | 190 | 361 | - | - |
| **SiCslA6** | - | - | 102 | 238 | - | - | 105 | 239 | - | - | - | - |
| **SiCslA7** | - | - | 8 | 189 | 47 | 283 | 3 | 130 | - | - | - | - |
| **SiCslA8** | - | - | 81 | 316 | 174 | 395 | 84 | 258 | - | - | - | - |
| **SiCslA9** | - | - | 131 | 366 | 224 | 441 | 134 | 312 | 197 | 365 | - | - |
| **SiCslA10** | - | - | 107 | 324 | 191 | 417 | - | - | - | - | - | - |
| **SiCslC1** | - | - | 228 | 463 | 321 | 534 | 231 | 410 | 286 | 462 | - | - |
| **SiCslC2** | - | - | 243 | 478 | 336 | 556 | 246 | 425 | 300 | 477 | - | - |
| **SiCslC3** | - | - | 234 | 477 | 305 | 545 | 271 | 440 | 241 | 393 | - | - |
| **SiCslC4** | - | - | 232 | 467 | 325 | 539 | 236 | 414 | 290 | 466 | - | - |
| **SiCslC5** | - | - | 276 | 511 | 369 | 587 | 335 | 510 | 280 | 458 | - | - |
| **SiCslC6** | - | - | 235 | 470 | 328 | 548 | 238 | 416 | 264 | 469 | - | - |
| **SiCslD1** | 424 | 1210 | - | - | 880 | 1041 | - | - | - | - | - | - |
| **SiCslD2** | 405 | 1172 | - | - | 840 | 1004 | - | - | - | - | 148 | 197 |
| **SiCslD3** | 297 | 656 | - | - | 730 | 889 | - | - | - | - | - | - |
|  | 669 | 1059 |  |  |  |  |  |  |  |  |  |  |
| **SiCslD4** | 383 | 1152 | - | - | - | - | - | - | - | - | 141 | 189 |
| **SiCslD5** | 382 | 1151 | - | - | 818 | 984 | - | - | - | - | 134 | 183 |
| **SiCslE1** | 2 | 204 | - | - | - | - | - | - | - | - | - | - |
|  | 197 | 349 |  |  |  |  |  |  |  |  |  |  |
|  | 342 | 497 |  |  |  |  |  |  |  |  |  |  |
| **SiCslE2** | 1 | 102 | - | - | - | - | - | - | - | - | - | - |
| **SiCslE3** | 91 | 408 | 234 | 480 | - | - | - | - | - | - | - | - |
|  | 390 | 720 |  |  |  |  |  |  |  |  |  |  |
| **SiCslE4** | 95 | 396 | - | - | - | - | - | - | - | - | - | - |
|  | 395 | 730 |  |  |  |  |  |  |  |  |  |  |
| **SiCslF1** | 163 | 506 | - | - | 400 | 699 | - | - | - | - | - | - |
|  | 501 | 866 |  |  |  |  |  |  |  |  |  |  |
| **SiCslF2** | 203 | 542 | - | - | - | - | - | - | - | - | - | - |
|  | 532 | 901 |  |  |  |  |  |  |  |  |  |  |
| **SiCslF3** | 166 | 507 | - | - | 407 | 710 | - | - | - | - | - | - |
|  | 496 | 865 |  |  |  |  |  |  |  |  |  |  |
| **SiCslF4** | 117 | 481 | - | - | - | - | - | - | - | - | - | - |
|  | 487 | 766 |  |  |  |  |  |  |  |  |  |  |
| **SiCslF5** | 162 | 500 | - | - | - | - | - | - | - | - | - | - |
|  | 492 | 861 |  |  |  |  |  |  |  |  |  |  |
| **SiCslF6** | 188 | 927 | - | - | - | - | - | - | - | - | - | - |
| **SiCslF7** | 131 | 470 | - | - | 363 | 673 | 329 | 450 | - | - | - | - |
|  | 463 | 831 |  |  |  |  |  |  |  |  |  |  |
| **SiCslH1** | 96 | 391 | - | - | - | - | - | - | - | - | - | - |
|  | 393 | 753 |  |  |  |  |  |  |  |  |  |  |
| **SiCslH2** | 106 | 415 | - | - | - | - | - | - | - | - | - | - |
|  | 412 | 778 |  |  |  |  |  |  |  |  |  |  |
| **SiCslJ1** | 102 | 397 | - | - | - | - | - | - | - | - | - | - |
|  | 389 | 762 |  |  |  |  |  |  |  |  |  |  |
| **SiCslJ2** | 102 | 399 | - | - | - | - | - | - | - | - | - | - |
|  | 394 | 648 |  |  |  |  |  |  |  |  |  |  |

**-** Not present
