# Supplementary material for: Integrative analysis and expression profiling of secondary cell wall genes in C4 biofuel model Setaria italica reveals targets for lignocellulose bioengineering
Source: Front Plant Sci. 2015 Nov 4;6:965. doi: 10.3389/fpls.2015.00965 (PMC4631826; doi:10.3389/fpls.2015.00965)
Supplement: Supplementary Table S5 — Details of various domains present in SiGsl proteins. [file Table5.DOC]

**Supplementary Table S5.** Details of various domains present in SiGsl proteins.

| **Protein** | **1,3-beta-glucan synthase component (PF02364)** | | **1,3-beta-glucan synthase subunit FKS1, domain-1 (PF14288)** | | **Vta1 like (PF04652)** | |
| --- | --- | --- | --- | --- | --- | --- |
|  | **Start** | **End** | **Start** | **End** | **Start** | **End** |
| **SiGsl1** | 258 | 346 | - | - | - | - |
|  | 339 | 1040 |  |  |  |  |
| **SiGsl2** | 867 | 1686 | 150 | 263 | - | - |
| **SiGsl3** | 886 | 1697 | 173 | 286 | - | - |
| **SiGsl4** | 1 | 678 | - | - | - | - |
| **SiGsl5** | 1030 | 1103 | 354 | 464 | - | - |
| **SiGsl6** | 807 | 903 | 78 | 191 | - | - |
|  | 892 | 1605 |  |  | - | - |
| **SiGsl7** | 967 | 1667 | 370 | 466 | - | - |
| **SiGsl8** | 1064 | 1861 | 335 | 448 | 58 | 209 |
| **SiGsl9** | 1 | 332 | - | - | - | - |
| **SiGsl10** | 1067 | 1765 | 352 | 465 | 69 | 223 |
| **SiGsl11** | 1065 | 1163 | 336 | 449 | 54 | 205 |
|  | 1154 | 1353 |  |  |  |  |
|  | 1354 | 1837 |  |  |  |  |
| **SiGsl12** | 472 | 556 | - | - | - | - |
|  | 544 | 1160 |  |  |  |  |

**-** Not present
