# Supplementary material for: Integrative analysis and expression profiling of secondary cell wall genes in C4 biofuel model Setaria italica reveals targets for lignocellulose bioengineering
Source: Front Plant Sci. 2015 Nov 4;6:965. doi: 10.3389/fpls.2015.00965 (PMC4631826; doi:10.3389/fpls.2015.00965)
Supplement: Supplementary Table S10 — Details of foxtail millet miRNAs identified to target the transcripts of lignocellulose pathway genes. [file Table10.DOC]

**Supplementary Table S10.** Details of foxtail millet miRNAs identified to target the transcripts of lignocellulose pathway genes.

| **miRNA** | **Target** | **Expectation** | **UPE** | **miRNA start** | **miRNA end** | **Target start** | **Target end** | **miRNA aligned fragment** | **Target aligned fragment** | **Inhibition** |
| --- | --- | --- | --- | --- | --- | --- | --- | --- | --- | --- |
| sit-miR156d-1 | *SiGsl3* | 3 | 20.432 | 1 | 20 | 980 | 999 | UUGACAGAAGAGAGUGAGCA | UGCUCAUUCUGUUCUUUCAA | Translation |
| sit-miR156d-2 | *SiGsl3* | 3 | 20.432 | 1 | 20 | 980 | 999 | UUGACAGAAGAGAGUGAGCA | UGCUCAUUCUGUUCUUUCAA | Translation |
| sit-miR395b | *SiGsl10* | 3 | 13.139 | 1 | 19 | 1676 | 1695 | UGAAGUG-UUUGGAGGAACU | AGUUCCUCCAAAGCAUUUUA | Cleavage |
| sit-miR114-npr | *SiF5H2* | 3 | 20.792 | 1 | 20 | 706 | 725 | CUGAAGUGUUUGGGGAACUC | GAGUUCUCCAAGCUCUUCGG | Cleavage |
| sit-miRn29 | *SiCslC2* | 3 | 23.989 | 1 | 24 | 783 | 806 | UCCAAAUUGCAGGUCGUUUUGAUU | AAUUGCAGCGGUCUGCAAUUUGGA | Cleavage |
