# Supplementary material for: Integrative analysis and expression profiling of secondary cell wall genes in C4 biofuel model Setaria italica reveals targets for lignocellulose bioengineering
Source: Front Plant Sci. 2015 Nov 4;6:965. doi: 10.3389/fpls.2015.00965 (PMC4631826; doi:10.3389/fpls.2015.00965)
Supplement: Supplementary Table S12 — The Ka/Ks ratios and estimated divergence time for homologous lignocellulose pathway proteins between Setaria italica and Panicum virgatum. [file Table12.DOC]

**Supplementary Table S12.** The Ka/Ks ratios and estimated divergence time for homologous lignocellulose pathway proteins between *Setaria italica* and *Panicum virgatum.*

| **Gene ID** | **Position on foxtail millet genome** | | | **Ortholog gene ID** | **Position on switchgrass genome** | | | **% identity** | **Ka** | **Ks** | **Ka/Ks** | **Time of divergence (MYA)** |
| --- | --- | --- | --- | --- | --- | --- | --- | --- | --- | --- | --- | --- |
|  | **Chr** | **Start** | **End** |  | **Chr** | **Start** | **End** |  |  |  |  |  |
| SiCslE3 | 2 | 34760317 | 34764865 | Pavir.Ab01773 | 01b | 34365472 | 34379745 | 100 | 0.01 | 0.09 | 0.10 | 6.8 |
| SiCslE3 | 2 | 34760317 | 34764865 | Pavir.Ab02265 | 01b | 42959735 | 42969495 | 97.37 | 0.04 | 0.08 | 0.45 | 6.5 |
| SiCslE3 | 2 | 34760317 | 34764865 | Pavir.Ba01326 | 02a | 16576022 | 16579016 | 97.22 | 0.01 | 0.04 | 0.20 | 3.2 |
| SiCslE4 | 2 | 34768795 | 34771790 | Pavir.Ab01773 | 01b | 34365472 | 34379745 | 100 | 0.06 | 0.04 | 1.47 | 3.3 |
| SiCslE4 | 2 | 34768795 | 34771790 | Pavir.Ab02265 | 01b | 42959735 | 42969495 | 97.37 | 0.01 | 0.05 | 0.19 | 4.1 |
| SiCslE4 | 2 | 34768795 | 34771790 | Pavir.Ba01326 | 02a | 16576022 | 16579016 | 97.22 | 0.05 | 0.06 | 0.89 | 4.4 |
| SiCslF6 | 6 | 4099554 | 4106215 | Pavir.Eb04036 | 05b | 75830604 | 75833382 | 100 | 0.05 | 0.04 | 1.22 | 3.2 |
| SiGsl11 | 9 | 32794970 | 32808573 | Pavir.Ba02323 | 02a | 33815850 | 33822680 | 95 | 0.04 | 0.06 | 0.61 | 4.8 |
| SiGsl2 | 1 | 42017703 | 42033495 | Pavir.J11684 | contig141350 | 119 | 2930 | 100 | 0.01 | 0.06 | 0.14 | 4.9 |
| SiGsl5 | 4 | 40020270 | 40033905 | Pavir.Eb03372 | 05b | 69159033 | 69162016 | 95 | 0.01 | 0.08 | 0.14 | 6.4 |
| SiGsl5 | 4 | 40020270 | 40033905 | Pavir.Ia04488 | 09a | 81711917 | 81719391 | 97.06 | 0.03 | 0.08 | 0.37 | 6.0 |
| SiGsl5 | 4 | 40020270 | 40033905 | Pavir.Ib03389 | 09b | 54509490 | 54515087 | 95.95 | 0.03 | 0.08 | 0.38 | 5.8 |
| SiGsl6 | 5 | 24116753 | 24126281 | Pavir.Db00045 | 04b | 682361 | 698386 | 95.56 | 0.03 | 0.09 | 0.33 | 6.8 |
| SiGsl7 | 5 | 24134551 | 24141849 | Pavir.Aa01654 | 01a | 21870540 | 21873706 | 97.5 | 0.02 | 0.04 | 0.40 | 3.3 |
| SiGsl7 | 5 | 24134551 | 24141849 | Pavir.Aa02339 | 01a | 43496821 | 43515960 | 96.49 | 0.02 | 0.05 | 0.35 | 3.8 |
| SiGsl7 | 5 | 24134551 | 24141849 | Pavir.Ab01748 | 01b | 33811356 | 33815226 | 95.92 | 0.03 | 0.05 | 0.54 | 4.0 |
| SiGsl7 | 5 | 24134551 | 24141849 | Pavir.Bb00770 | 02b | 12674894 | 12678374 | 95.92 | 0.02 | 0.05 | 0.38 | 3.5 |
| SiGsl7 | 5 | 24134551 | 24141849 | Pavir.Bb01517 | 02b | 35792006 | 35798360 | 98.08 | 0.02 | 0.06 | 0.26 | 5.0 |
| SiGsl7 | 5 | 24134551 | 24141849 | Pavir.Bb02500 | 02b | 58496788 | 58508820 | 96 | 0.02 | 0.06 | 0.26 | 4.7 |
| SiGsl7 | 5 | 24134551 | 24141849 | Pavir.Ca00351 | 03a | 4144612 | 4148815 | 97.56 | 0.02 | 0.07 | 0.22 | 5.5 |
| SiGsl7 | 5 | 24134551 | 24141849 | Pavir.Ca00758 | 03a | 8487517 | 8493210 | 96.15 | 0.01 | 0.04 | 0.35 | 3.2 |
| SiGsl7 | 5 | 24134551 | 24141849 | Pavir.Ca02451 | 03a | 41226986 | 41232016 | 97.06 | 0.02 | 0.04 | 0.39 | 3.4 |
| SiGsl7 | 5 | 24134551 | 24141849 | Pavir.Ca02635 | 03a | 43942008 | 43946232 | 96.97 | 0.02 | 0.05 | 0.35 | 3.8 |
| SiGsl7 | 5 | 24134551 | 24141849 | Pavir.Da00655 | 04a | 9782777 | 9790202 | 97.37 | 0.09 | 0.09 | 1.03 | 6.7 |
| SiGsl7 | 5 | 24134551 | 24141849 | Pavir.Db01909 | 04b | 42022822 | 42030967 | 97.92 | 0.13 | 0.08 | 1.60 | 6.3 |
| SiGsl7 | 5 | 24134551 | 24141849 | Pavir.Ga00922 | 07a | 11050528 | 11055266 | 95.83 | 0.10 | 0.08 | 1.31 | 5.8 |
| SiGsl7 | 5 | 24134551 | 24141849 | Pavir.Ga02221 | 07a | 33633050 | 33650116 | 95.56 | 0.07 | 0.07 | 0.94 | 5.4 |
| SiGsl7 | 5 | 24134551 | 24141849 | Pavir.Ga02616 | 07a | 50095554 | 50104324 | 97.87 | 0.09 | 0.07 | 1.33 | 5.0 |
| SiGsl7 | 5 | 24134551 | 24141849 | Pavir.Gb02513 | 07b | 47304533 | 47345515 | 100 | 0.09 | 0.05 | 1.67 | 4.0 |
| SiGsl7 | 5 | 24134551 | 24141849 | Pavir.Hb01249 | 08b | 34358639 | 34361924 | 95.65 | 0.09 | 0.07 | 1.29 | 5.2 |
| SiGsl7 | 5 | 24134551 | 24141849 | Pavir.Ia00898 | 09a | 9212407 | 9219786 | 98.08 | 0.09 | 0.07 | 1.21 | 5.5 |
| SiGsl7 | 5 | 24134551 | 24141849 | Pavir.J11078 | contig13661 | 794 | 9868 | 97.96 | 0.08 | 0.08 | 1.12 | 5.8 |
| SiGsl7 | 5 | 24134551 | 24141849 | Pavir.J14423 | contig164025 | 96 | 2116 | 95.74 | 0.08 | 0.05 | 1.76 | 3.7 |
| SiGsl7 | 5 | 24134551 | 24141849 | Pavir.J20269 | contig22023 | 2012 | 8822 | 97.92 | 0.08 | 0.04 | 1.89 | 3.4 |
| SiGsl7 | 5 | 24134551 | 24141849 | Pavir.J22381 | contig24389 | 947 | 5507 | 96.23 | 0.08 | 0.05 | 1.77 | 3.7 |
| SiGsl7 | 5 | 24134551 | 24141849 | Pavir.J33651 | contig42100 | 392 | 6004 | 96.43 | 0.13 | 0.04 | 3.03 | 3.2 |
| SiGsl7 | 5 | 24134551 | 24141849 | Pavir.J37914 | contig71757 | 8-4614 |  | 95.74 | 0.13 | 0.05 | 2.30 | 4.2 |
| SiGsl7 | 5 | 24134551 | 24141849 | Pavir.J40238 | contig91462 | 2201 | 3901 | 96 | 0.13 | 0.06 | 2.21 | 4.4 |
| SiGsl8 | 5 | 32798706 | 32805320 | Pavir.Aa02896 | 01a | 62299746 | 62304312 | 100 | 0.13 | 0.06 | 2.05 | 4.7 |
| SiGsl8 | 5 | 32798706 | 32805320 | Pavir.Ab02265 | 01b | 42959735 | 42969495 | 100 | 0.02 | 0.07 | 0.24 | 5.0 |
| SiGsl8 | 5 | 32798706 | 32805320 | Pavir.Db01100 | 04b | 20130135 | 20134390 | 100 | 0.05 | 0.07 | 0.81 | 5.2 |
| SiGsl8 | 5 | 32798706 | 32805320 | Pavir.Ga00647 | 07a | 8164315 | 8168253 | 96.97 | 0.05 | 0.07 | 0.81 | 5.2 |
| SiGsl8 | 5 | 32798706 | 32805320 | Pavir.Gb00583 | 07b | 6152859 | 6154346 | 100 | 0.05 | 0.07 | 0.77 | 5.4 |
| SiGsl8 | 5 | 32798706 | 32805320 | Pavir.J09889 | contig127089 | 3-2490 |  | 100 | 0.05 | 0.07 | 0.75 | 5.6 |
| SiGsl8 | 5 | 32798706 | 32805320 | Pavir.J18859 | contig20589 | 2-8618 |  | 97.44 | 0.02 | 0.07 | 0.28 | 5.7 |
| SiGsl8 | 5 | 32798706 | 32805320 | Pavir.J30348 | contig353778 | 464 | 1189 | 96.97 | 0.13 | 0.07 | 1.84 | 5.4 |
| SiGsl8 | 5 | 32798706 | 32805320 | Pavir.J31147 | contig367385 | 14 | 1049 | 100 | 0.04 | 0.07 | 0.63 | 5.0 |
| SiHCT2 | 7 | 24089783 | 24094559 | Pavir.Ab02689 | 01b | 48627505 | 48628167 | 95.35 | 0.09 | 0.06 | 1.53 | 4.7 |
| SiPAL1 | 1 | 31757351 | 31761757 | Pavir.Ab02345 | 01b | 44223869 | 44224923 | 95.09 | 0.06 | 0.06 | 0.99 | 4.4 |
| SiPAL4 | 1 | 31795010 | 31797372 | Pavir.Aa01127 | 01a | 14695280 | 14697646 | 95.4 | 0.03 | 0.05 | 0.50 | 4.1 |
| SiPAL5 | 1 | 31806380 | 31808854 | Pavir.Aa01127 | 01a | 14695280 | 14697646 | 95.4 | 0.01 | 0.05 | 0.21 | 4.1 |
| Si4CL17 | 9 | 56988663 | 56992528 | Pavir.Ab02367 | 01b | 44380581 | 44392275 | 100 | 0.10 | 0.06 | 1.56 | 4.9 |
| Si4CL17 | 9 | 56988663 | 56992528 | Pavir.Ba01589 | 02a | 20530346 | 20533844 | 96.88 | 0.06 | 0.05 | 1.19 | 3.7 |
| SiCAD10 | 7 | 8002006 | 8003815 | Pavir.Ba01618 | 02a | 21013080 | 21013731 | 95.12 | 0.02 | 0.05 | 0.49 | 3.7 |
| SiCCR07 | 5 | 3359417 | 3361681 | Pavir.Ba01694 | 02a | 21582691 | 21604357 | 97.09 | 0.05 | 0.05 | 0.99 | 4.1 |
| SiCCR07 | 5 | 3359417 | 3361681 | Pavir.Ba01697 | 02a | 21597342 | 21601054 | 97.09 | 0.01 | 0.06 | 0.15 | 4.7 |
| SiCCR19 | 5 | 3359417 | 3361681 | Pavir.Ab01931 | 01b | 37381915 | 37386680 | 97.22 | 0.02 | 0.07 | 0.37 | 5.0 |
| SiCCR2 | 1 | 41081688 | 41083214 | Pavir.Ab01121 | 01b | 16618708 | 16632767 | 97.06 | 0.02 | 0.05 | 0.44 | 4.2 |
| SiCCR25 | 9 | 24269116 | 24271741 | Pavir.Ba01694 | 02a | 21582691 | 21604357 | 97.09 | 0.09 | 0.06 | 1.44 | 5.0 |
| SiCCR25 | 9 | 24269116 | 24271741 | Pavir.Ba01697 | 02a | 21597342 | 21601054 | 97.09 | 0.13 | 0.07 | 1.88 | 5.2 |
| **Mean** | | | | | | | | | **0.05** | **0.06** | **0.91** | **4.7** |
