# Supplementary material for: Integrative analysis and expression profiling of secondary cell wall genes in C4 biofuel model Setaria italica reveals targets for lignocellulose bioengineering
Source: Front Plant Sci. 2015 Nov 4;6:965. doi: 10.3389/fpls.2015.00965 (PMC4631826; doi:10.3389/fpls.2015.00965)
Supplement: Supplementary Table S13 — The Ka/Ks ratios and estimated divergence time for homologous lignocellulose pathway proteins between Setaria italica and Sorghum bicolor. [file Table13.DOC]

**Supplementary Table S13.** The Ka/Ks ratios and estimated divergence time for homologous lignocellulose pathway proteins between *Setaria italica* and *Sorghum bicolor.*

| **Gene ID** | **Position on foxtail millet genome** | | | **Ortholog gene ID** | **Position on sorghum genome** | | | **% identity** | **Ka** | **Ks** | **Ka/Ks** | **Time of divergence (MYA)** |
| --- | --- | --- | --- | --- | --- | --- | --- | --- | --- | --- | --- | --- |
|  | **Chr** | **Start** | **End** |  | **Chr** | **Start** | **End** |  |  |  |  |  |
| SiCesA11 | 9 | 1087917 | 1093521 | Sobic.001G303200 | 1 | 51491518 | 51494018 | 100 | 0.05 | 0.35 | 0.13 | 26.7 |
| SiCesA11 | 9 | 1087917 | 1093521 | Sobic.010G177400 | 10 | 51287887 | 51291405 | 96.47 | 0.09 | 0.32 | 0.27 | 24.5 |
| SiCesA13 | 9 | 17170732 | 17174910 | Sobic.001G303200 | 1 | 51491518 | 51494018 | 100 | 0.03 | 0.19 | 0.15 | 14.3 |
| SiCesA13 | 9 | 17170732 | 17174910 | Sobic.010G177400 | 10 | 51287887 | 51291405 | 96.47 | 0.03 | 0.41 | 0.07 | 31.8 |
| SiCslA2 | 1 | 37889983 | 37894059 | Sobic.010G197300 | 10 | 53787386 | 53794028 | 100 | 0.03 | 0.27 | 0.10 | 20.7 |
| SiCslA3 | 2 | 16115171 | 16117707 | Sobic.004G111000 | 4 | 10950666 | 10957103 | 95.83 | 0.09 | 0.25 | 0.38 | 19.0 |
| SiCslA6 | 4 | 32756777 | 32758489 | Sobic.001G252700 | 1 | 27402757 | 27408552 | 95.65 | 0.06 | 0.44 | 0.14 | 33.8 |
| SiCslA7 | 6 | 28191242 | 28193384 | Sobic.010G072000 | 10 | 5774844 | 5779072 | 97.73 | 0.07 | 0.30 | 0.24 | 23.2 |
| SiCslA7 | 6 | 28191242 | 28193384 | Sobic.010G189000 | 10 | 52662269 | 52668307 | 97.62 | 0.05 | 0.29 | 0.18 | 22.0 |
| SiCslC2 | 2 | 1951845 | 1955090 | Sobic.004G111000 | 4 | 10950666 | 10957103 | 95.83 | 0.11 | 0.46 | 0.23 | 35.7 |
| SiGsl11 | 9 | 32794970 | 32808573 | Sobic.001G017700 | 1 | 1497940 | 1506192 | 97.78 | 0.04 | 0.47 | 0.08 | 36.1 |
| SiGsl11 | 9 | 32794970 | 32808573 | Sobic.001G423000 | 1 | 63176509 | 63183476 | 100 | 0.03 | 0.35 | 0.09 | 26.7 |
| SiGsl11 | 9 | 32794970 | 32808573 | Sobic.001G132900 | 1 | 10442769 | 10449160 | 100 | 0.05 | 0.54 | 0.10 | 41.3 |
| SiGsl11 | 9 | 32794970 | 32808573 | Sobic.002G141900 | 2 | 23086128 | 23138337 | 95.65 | 0.05 | 0.47 | 0.11 | 36.0 |
| SiGsl11 | 9 | 32794970 | 32808573 | Sobic.003G312100 | 3 | 64040052 | 64067029 | 95.83 | 0.07 | 0.61 | 0.12 | 47.2 |
| SiGsl11 | 9 | 32794970 | 32808573 | Sobic.005G165800 | 5 | 54633887 | 54642111 | 97.96 | 0.04 | 0.27 | 0.16 | 21.1 |
| SiGsl11 | 9 | 32794970 | 32808573 | Sobic.006G040500 | 6 | 25483476 | 25535468 | 95.83 | 0.05 | 0.31 | 0.17 | 24.1 |
| SiGsl11 | 9 | 32794970 | 32808573 | Sobic.006G205400 | 6 | 56452109 | 56459910 | 97.14 | 0.08 | 0.35 | 0.24 | 26.8 |
| SiGsl11 | 9 | 32794970 | 32808573 | Sobic.009G069000 | 9 | 7608224 | 7622520 | 97.92 | 0.02 | 0.33 | 0.07 | 25.4 |
| SiGsl11 | 9 | 32794970 | 32808573 | Sobic.009G045000 | 9 | 4333710 | 4342315 | 95.83 | 0.05 | 0.17 | 0.26 | 13.4 |
| SiGsl11 | 9 | 32794970 | 32808573 | Sobic.010G144900 | 10 | 40105138 | 40113856 | 97.92 | 0.05 | 0.35 | 0.15 | 26.7 |
| SiGsl2 | 1 | 42017703 | 42033495 | Sobic.009G259400 | 9 | 59287207 | 59290490 | 96.97 | 0.07 | 0.61 | 0.12 | 46.9 |
| SiGsl5 | 4 | 40020270 | 40033905 | Sobic.001G132600 | 1 | 10398096 | 10422641 | 95.24 | 0.05 | 0.27 | 0.16 | 21.1 |
| SiGsl5 | 4 | 40020270 | 40033905 | Sobic.002G072000 | 2 | 7325410 | 7337170 | 96.88 | 0.08 | 0.48 | 0.18 | 36.6 |
| SiGsl5 | 4 | 40020270 | 40033905 | Sobic.004G111600 | 4 | 11076069 | 11093920 | 95.24 | 0.08 | 0.28 | 0.28 | 21.3 |
| SiGsl7 | 5 | 24134551 | 24141849 | Sobic.001G270800 | 1 | 45064575 | 45070605 | 97.22 | 0.09 | 0.29 | 0.32 | 22.1 |
| SiGsl7 | 5 | 24134551 | 24141849 | Sobic.001G423000 | 1 | 63176509 | 63183476 | 97.14 | 0.06 | 0.35 | 0.16 | 26.7 |
| SiGsl7 | 5 | 24134551 | 24141849 | Sobic.002G118200 | 2 | 14736780 | 14753332 | 95.35 | 0.06 | 0.32 | 0.18 | 24.5 |
| SiGsl7 | 5 | 24134551 | 24141849 | Sobic.002G117900 | 2 | 14690724 | 14710543 | 94.87 | 0.09 | 0.23 | 0.39 | 18.0 |
| SiGsl7 | 5 | 24134551 | 24141849 | Sobic.003G252500 | 3 | 59117697 | 59124654 | 97.3 | 0.04 | 0.19 | 0.20 | 14.6 |
| SiGsl7 | 5 | 24134551 | 24141849 | Sobic.006G040500 | 6 | 25483476 | 25535468 | 96 | 0.09 | 0.19 | 0.44 | 15.0 |
| SiGsl7 | 5 | 24134551 | 24141849 | Sobic.007G074600 | 7 | 8264601 | 8290211 | 100 | 0.06 | 0.53 | 0.12 | 40.9 |
| Si4CL16 | 9 | 55993419 | 55997948 | Sobic.007G136500 | 7 | 55062479 | 55065007 | 97.14 | 0.05 | 0.21 | 0.22 | 16.0 |
| Si4CL3 | 2 | 20877834 | 20883712 | Sobic.003G005000 | 3 | 451687 | 454277 | 95.83 | 0.03 | 0.43 | 0.07 | 33.2 |
| SiCAD12 | 7 | 29708312 | 29711863 | Sobic.002G187500 | 2 | 57144156 | 57146730 | 100 | 0.07 | 0.71 | 0.10 | 54.6 |
| SiCCoAOMT2 | 6 | 4831123 | 4834209 | Sobic.007G043200 | 7 | 4287589 | 4291883 | 96.12 | 0.09 | 0.25 | 0.38 | 19.0 |
| SiCCoAOMT3 | 6 | 31909578 | 31910910 | Sobic.007G217100 | 7 | 63308318 | 63316703 | 95.12 | 0.04 | 0.51 | 0.07 | 39.1 |
| SiCCR12 | 4 | 3568762 | 3571563 | Sobic.007G157700 | 7 | 57921174 | 57924141 | 100 | 0.07 | 0.28 | 0.24 | 21.7 |
| SiCCR17 | 4 | 32958815 | 32961138 | Sobic.007G141200 | 7 | 55767684 | 55774919 | 95.28 | 0.09 | 0.33 | 0.27 | 25.5 |
| SiCCR17 | 4 | 32958815 | 32961138 | Sobic.010G195400 | 10 | 53593422 | 53595376 | 96.97 | 0.09 | 0.22 | 0.40 | 17.0 |
| SiCCR17 | 4 | 32958815 | 32961138 | Sobic.010G195300 | 10 | 53587707 | 53595376 | 96.97 | 0.08 | 0.43 | 0.17 | 33.2 |
| **Mean** | | | | | | | | | **0.06** | **0.36** | **0.19** | **27.4** |
