# Supplementary material for: Integrative analysis and expression profiling of secondary cell wall genes in C4 biofuel model Setaria italica reveals targets for lignocellulose bioengineering
Source: Front Plant Sci. 2015 Nov 4;6:965. doi: 10.3389/fpls.2015.00965 (PMC4631826; doi:10.3389/fpls.2015.00965)
Supplement: Supplementary Table S14 — The Ka/Ks ratios and estimated divergence time for homologous lignocellulose pathway proteins between Setaria italica and Zea mays. [file Table14.DOC]

**Supplementary Table S14.** The Ka/Ks ratios and estimated divergence time for homologous lignocellulose pathway proteins between *Setaria italica* and *Zea mays.*

| **Gene ID** | **Position on foxtail millet genome** | | | **Ortholog gene ID** | **Position on maize genome** | | | **% identity** | **Ka** | **Ks** | **Ka/Ks** | **Time of divergence (MYA)** |
| --- | --- | --- | --- | --- | --- | --- | --- | --- | --- | --- | --- | --- |
|  | **Chr** | **Start** | **End** |  | **Chr** | **Start** | **End** |  |  |  |  |  |
| SiCesA11 | 9 | 1087917 | 1093521 | GRMZM2G089121 | 1 | 89006906 | 89009728 | 98.7 | 0.05 | 0.36 | 0.14 | 27.4 |
| SiCesA11 | 9 | 1087917 | 1093521 | GRMZM2G095206 | 9 | 89593729 | 89597247 | 95.79 | 0.04 | 0.19 | 0.19 | 14.7 |
| SiCesA12 | 9 | 2474927 | 2480592 | GRMZM2G424832 | 7 | 18620330 | 18626341 | 100 | 0.05 | 0.51 | 0.10 | 39.3 |
| SiCesA12 | 9 | 2474927 | 2480592 | GRMZM2G125891 | 7 | 18620632 | 18622051 | 100 | 0.03 | 0.35 | 0.09 | 27.1 |
| SiCesA13 | 9 | 17170732 | 17174910 | GRMZM2G089121 | 1 | 89006906 | 89009728 | 98.7 | 0.11 | 0.26 | 0.43 | 20.3 |
| SiCesA13 | 9 | 17170732 | 17174910 | GRMZM2G095206 | 9 | 89593729 | 89597247 | 95.79 | 0.06 | 0.37 | 0.16 | 28.4 |
| SiCesA6 | 4 | 32901724 | 32907564 | GRMZM2G141693 | 2 | 214978223 | 214984356 | 95.83 | 0.07 | 0.28 | 0.26 | 21.2 |
| SiCslA3 | 2 | 16115171 | 16117707 | AC190716.3 | 5 | 8844621 | 8847253 | 100 | 0.05 | 0.37 | 0.15 | 28.3 |
| SiCslA7 | 6 | 28191242 | 28193384 | GRMZM2G119248 | 1 | 156545682 | 156558520 | 100 | 0.04 | 0.34 | 0.12 | 26.2 |
| SiCslA8 | 9 | 21337084 | 21343333 | GRMZM2G405567 | 5 | 206893583 | 206896941 | 97.73 | 0.04 | 0.50 | 0.07 | 38.2 |
| SiCslC2 | 2 | 1951845 | 1955090 | AC190716.3 | 5 | 8844621 | 8847253 | 100 | 0.07 | 0.37 | 0.18 | 28.4 |
| SiCslF7 | 9 | 24565440 | 24568994 | GRMZM2G424832 | 7 | 18620330 | 18626341 | 100 | 0.06 | 0.59 | 0.10 | 45.4 |
| SiCslF7 | 9 | 24565440 | 24568994 | GRMZM2G125891 | 7 | 18620632 | 18622051 | 100 | 0.07 | 0.51 | 0.15 | 38.9 |
| SiCslJ1 | 3 | 42498683 | 42501441 | GRMZM2G110145 | 10 | 77282591 | 77289441 | 96.97 | 0.05 | 0.33 | 0.17 | 25.6 |
| SiCslJ2 | 3 | 42528663 | 42531387 | GRMZM2G110145 | 10 | 77282591 | 77289441 | 96.97 | 0.03 | 0.22 | 0.15 | 17.1 |
| SiF5H2 | 9 | 13855140 | 13859537 | AC214524.3 | 1 | 77974931 | 77976981 | 95.12 | 0.08 | 0.32 | 0.25 | 25.0 |
| SiGsl11 | 9 | 32794970 | 32808573 | GRMZM5G843765 | 1 | 35971538 | 35989552 | 100 | 0.08 | 0.70 | 0.12 | 54.2 |
| SiGsl11 | 9 | 32794970 | 32808573 | GRMZM2G585025 | 3 | 138364036 | 138411598 | 95.65 | 0.03 | 0.35 | 0.07 | 26.7 |
| SiGsl11 | 9 | 32794970 | 32808573 | GRMZM5G878139 | 4 | 4398962 | 4410023 | 95.24 | 0.06 | 0.29 | 0.20 | 22.2 |
| SiGsl11 | 9 | 32794970 | 32808573 | GRMZM2G071071 | 5 | 3196192 | 3207509 | 97.87 | 0.03 | 0.34 | 0.08 | 26.1 |
| SiGsl11 | 9 | 32794970 | 32808573 | GRMZM2G075023 | 8 | 165780662 | 165790509 | 97.96 | 0.06 | 0.37 | 0.16 | 28.4 |
| SiGsl11 | 9 | 32794970 | 32808573 | GRMZM2G021864 | 10 | 81549136 | 81571387 | 95.83 | 0.06 | 0.32 | 0.18 | 24.3 |
| SiGsl11 | 9 | 32794970 | 32808573 | GRMZM2G052166 | 10 | 101748275 | 101751536 | 95.74 | 0.11 | 0.46 | 0.23 | 35.7 |
| SiGsl3 | 2 | 1512780 | 1516075 | GRMZM5G810484 | 3 | 196550950 | 196551315 | 95.22 | 0.11 | 0.48 | 0.22 | 36.6 |
| SiGsl5 | 4 | 40020270 | 40033905 | GRMZM2G300944 | 5 | 12398192 | 12403243 | 96.88 | 0.08 | 0.42 | 0.20 | 32.5 |
| SiGsl7 | 5 | 24134551 | 24141849 | GRMZM2G057031 | 4 | 159900689 | 159911939 | 95.56 | 0.09 | 0.37 | 0.25 | 28.4 |
| SiGsl7 | 5 | 24134551 | 24141849 | GRMZM2G178758 | 4 | 157223502 | 157238481 | 95 | 0.05 | 0.28 | 0.18 | 21.4 |
| SiGsl7 | 5 | 24134551 | 24141849 | GRMZM2G477694 | 5 | 26998422 | 27013206 | 97.22 | 0.06 | 0.29 | 0.20 | 22.7 |
| SiHCT2 | 7 | 24089783 | 24094559 | GRMZM2G314328 | 1 | 234788955 | 234793861 | 95.74 | 0.06 | 0.26 | 0.22 | 19.9 |
| SiPAL1 | 1 | 31757351 | 31761757 | GRMZM2G160541 | 4 | 143404330 | 143408521 | 95.82 | 0.07 | 0.24 | 0.28 | 18.3 |
| SiPAL8 | 7 | 25018087 | 25020976 | GRMZM2G160541 | 4 | 143404330 | 143408521 | 95.11 | 0.03 | 0.34 | 0.08 | 26.1 |
| Si4CL15 | 9 | 55988807 | 55992159 | GRMZM2G095887 | 6 | 126007413 | 126010452 | 100 | 0.05 | 0.42 | 0.11 | 32.5 |
| SiC4H1 | 1 | 21991841 | 21993582 | GRMZM2G027041 | 7 | 94672495 | 94674312 | 96.88 | 0.11 | 0.25 | 0.43 | 19.5 |
| SiCAD4 | 2 | 29826314 | 29828093 | GRMZM5G886863 | 8 | 150343314 | 150344824 | 100 | 0.06 | 0.37 | 0.16 | 28.4 |
| SiCCR1 | 1 | 5911546 | 5913992 | GRMZM2G092917 | 4 | 235644053 | 235644469 | 97.37 | 0.12 | 0.28 | 0.44 | 21.3 |
| SiCCR10 | 2 | 37919279 | 37921793 | GRMZM2G130332 | 1 | 150679023 | 150692114 | 100 | 0.07 | 0.30 | 0.24 | 22.8 |
| SiCCR16 | 4 | 32955075 | 32958532 | GRMZM2G092917 | 4 | 235644053 | 235644469 | 100 | 0.08 | 0.32 | 0.25 | 24.2 |
| SiCCR20 | 5 | 40408588 | 40410432 | GRMZM2G410865 | 5 | 2604766 | 2606348 | 95.73 | 0.08 | 0.47 | 0.18 | 36.1 |
| **Mean** | | | | | | | | | **0.06** | **0.36** | **0.19** | **27.9** |
