# Supplementary material for: Integrative analysis and expression profiling of secondary cell wall genes in C4 biofuel model Setaria italica reveals targets for lignocellulose bioengineering
Source: Front Plant Sci. 2015 Nov 4;6:965. doi: 10.3389/fpls.2015.00965 (PMC4631826; doi:10.3389/fpls.2015.00965)
Supplement: Supplementary Table S15 — The Ka/Ks ratios and estimated divergence time for tandemly duplicated lignocellulose pathway proteins. [file Table15.DOC]

**Supplementary Table S15.** The Ka/Ks ratios and estimated divergence time for tandemly duplicated lignocellulose pathway proteins*.*

| **Paralog details** | | | | | | | | | | **Distance (kb)** | **Ka** | **Ks** | **Ka/Ks** | **Time of divergence (MYA)** |
| --- | --- | --- | --- | --- | --- | --- | --- | --- | --- | --- | --- | --- | --- | --- |
| **Gene ID** | **Phytozome ID** | **Chr** | **Start** | **End** | **Gene ID** | **Phytozome ID** | **Chr** | **Start** | **End** |
| SiCslE3 | Si029066m.g | 2 | 34760317 | 34764865 | SiCslE4 | Si029057m.g | 2 | 34768795 | 34771790 | 3.9 | 0.05 | 0.35 | 0.14 | 26.9 |
| SiPAL4 | Si016478m.g | 1 | 31795010 | 31797372 | SiPAL5 | Si016475m.g | 1 | 31806380 | 31808854 | 9.0 | 0.06 | 0.34 | 0.18 | 26.2 |
| SiPAL8 | Si012256m.g | 7 | 25018087 | 25020976 | SiPAL9 | Si009509m.g | 7 | 25037649 | 25040471 | 16.7 | 0.03 | 0.32 | 0.09 | 24.6 |
| Si4CL11 | Si039889m.g | 9 | 12773750 | 12776763 | Si4CL12 | Si034944m.g | 9 | 12782712 | 12788175 | 5.9 | 0.04 | 0.33 | 0.12 | 25.4 |
| SiCCoAOMT3 | Si014344m.g | 6 | 31909578 | 31910910 | SiCCoAOMT4 | Si015292m.g | 6 | 31918639 | 31919497 | 7.7 | 0.05 | 0.34 | 0.15 | 26.2 |
| SiCAD2 | Si030293m.g | 2 | 29729962 | 29732080 | SiCAD3 | Si030289m.g | 2 | 29734467 | 29736353 | 2.4 | 0.05 | 0.35 | 0.14 | 26.9 |
| SiCAD8 | Si013626m.g | 6 | 1901210 | 1905419 | SiCAD9 | Si015115m.g | 6 | 1924647 | 1926123 | 19.2 | 0.03 | 0.32 | 0.09 | 24.6 |
| **Mean** | | | | | | | | | | | **0.04** | **0.34** | **0.13** | **25.8** |
